# C/EBPγ induces epithelial-mesenchymal transition and facilitates DNA double-strand break repair in lung adenocarcinoma cells

**DOI:** 10.1038/s41420-026-03181-0

**Published:** 2026-06-02

**Authors:** Minoru Terashima, Ryusuke Suzuki, Kusuma Suphakhong, Tatsunori Nishimura, Eerdunduleng, Takahisa Takino, Shin-Ichi Horike, Takumi Nishiuchi, Shoichiro Tange, Akihiko Ishimura, Dominic C. Voon, Goro Sashida, Takeshi Suzuki

**Affiliations:** 1 Division of Functional Genomics, Cancer Research Institute, Kanazawa University, Kanazawa, Ishikawa Japan; 2 Laboratory of Transcriptional Regulation in Leukemogenesis, International Research Center for Medical Sciences, Kumamoto University, Kumamoto, Japan; 3 Department of Neurodegenerative Disease, Beckman Research Institute, City of Hope, Duarte, CA USA; 4 Division of Cancer Biology, Nagoya University Graduate School of Medicine, Nagoya, Aichi Japan; 5 Division of Education for Global Standard, Institute of Liberal Arts and Science, Kanazawa University, Kanazawa, Ishikawa Japan; 6 Division of Integrated Omics research, Bioscience Core Facility Research Center for Experimental Modeling of Human Disease, Kanazawa University, Kanazawa, Ishikawa Japan; 7 Department of Medical Genome Sciences, Research Institute for Frontier Medicine, Sapporo Medical University School of Medicine, Sapporo, Japan; 8 Institute for Frontier Science Initiative, Kanazawa University, Kanazawa, Ishikawa Japan; 9 Present Address: Stem Cell Regulation Project, Tokyo Metropolitan Institute of Medical Science, Tokyo, Japan

**Keywords:** Oncogenes, Mechanisms of disease

## Abstract

Epithelial-mesenchymal transition (EMT) plays a crucial role in cancer progression and is driven by EMT–inducing transcription factors (EMT-TFs). Although core EMT-TFs are well characterized, transcription factors that couple EMT to malignant phenotypes remain incompletely defined. Here, we identified C/EBPγ as a novel inducer of EMT in lung adenocarcinoma cells by examining regions of extensive histone H3 lysine 4 trimethylation occupancy that are often a hallmark of cellular identity. Ectopic expression of C/EBPγ induced mesenchymal-like morphologies and EMT-associated gene-expression profiles, whereas its RNAi knockdown attenuated EMT. Interestingly, domain mapping showed that the leucine zipper domain, but not the DNA-binding domain, was essential for C/EBPγ-mediated EMT, indicating a novel DNA-binding-independent mechanism involving critical partner proteins. Co-immunoprecipitation and mass spectrometry identified C/EBPβ and the non-homologous end joining (NHEJ) factors XRCC5 and XRCC6 as C/EBPγ-interacting proteins. C/EBPγ promotes EMT, at least in part, by antagonizing the EMT-suppressive activity of C/EBPβ. In parallel, C/EBPγ promoted XRCC6 recruitment to etoposide-induced DNA double-strand break (DSB) sites, enhanced NHEJ activity, facilitated DSB repair, and increased survival under genotoxic stress induced by anticancer drug treatment. Collectively, these findings indicate that C/EBPγ may play a role in linking EMT-associated transcriptional changes with enhanced DNA repair capacity, which could have implications for chemoresistance in lung adenocarcinoma.

## Introduction

Epithelial–mesenchymal transition (EMT) is a dynamic and reversible cell-state transition that enables carcinoma cells to acquire enhanced motility, invasive capacity, and phenotypic plasticity [1–3]. This plasticity contributes to tumor heterogeneity and the ability of cancer cells to adapt to environmental and therapeutic pressures. EMT is orchestrated by EMT-inducing transcription factors (EMT-TFs), including members of the SNAIL, TWIST, and ZEB families, which coordinate broad transcriptional and epigenomic remodeling during cell-fate transitions. Although several EMT-TFs have been extensively characterized, accumulating evidence [4–6], suggests that additional transcriptional regulators remain to be identified, particularly those that may fine-tune EMT progression or integrate EMT programs with broader cellular responses relevant to malignant phenotypes during cancer progression.

Several approaches have been used to identify these transcription factors. One approach involves identifying TFs that repress E-cadherin expression [7]. Another focuses on TFs with elevated expression in mesenchymal cancer cell lines or high-malignancy cancer types [8, 9]. In addition, research using in vivo models has further highlighted the importance of discovering EMT-TFs, providing clinically valuable insights into understanding the mechanisms of malignancy [2, 4]. Nevertheless, due to the inherent limitations of each approach, it is highly probable that undiscovered EMT-TFs remain to be identified. To address these limitations, we explored an alternative approach based on epigenetic features that our research group has focused on. We previously showed that histone methyl-modifying enzymes play a significant role in TGF-β-induced EMT of cancer cells [10–12], suggesting that specific histone methylation patterns, such as the breadth of H3K4me3 domains, may mark genes critical for EMT regulation. Importantly, the breadth of H3K4me3 domains has been shown to correlate with genes defining cell identity [13, 14]. Based on this, we hypothesized that genes whose H3K4me3 domains broaden during EMT induction could include key EMT regulators. Through this approach, we identified C/EBPγ as a candidate EMT-associated transcription factor in A549 lung adenocarcinoma (LUAD) cells undergoing TGF-β-induced EMT. Previous reports have suggested that C/EBPγ can suppress cell senescence and inflammatory gene expression [15], but its role in EMT has not been reported. Our data indicate that C/EBPγ promotes EMT by opposing the inhibitory effects of its family member C/EBPβ. Additionally, C/EBPγ augmented non-homologous end joining (NHEJ) activity during etoposide-induced DNA double-strand breaks (DSBs) and increased cell survival under anticancer drug-induced DNA damage. Collectively, these data imply that C/EBPγ may contribute to the malignancy of pulmonary epithelial cancer cells by augmenting their DNA repair capacity during EMT.

## Results

### Novel EMT-TF candidates were identified through the broadest H3K4me3 domains during TGF-β-induced EMT

**Fig. 1:**
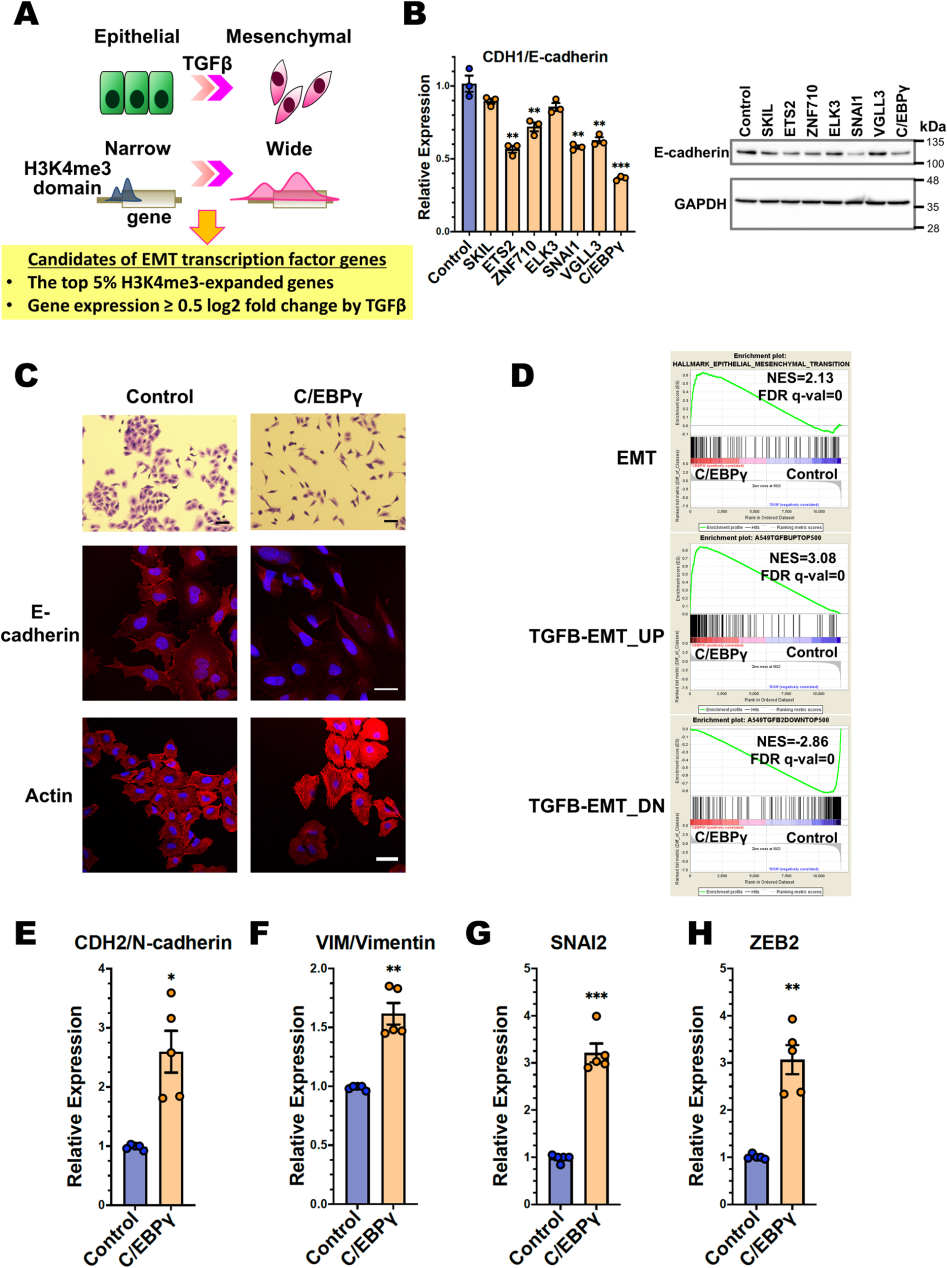
TGF-β-induced expansion of H3K4me3 domains identifies C/EBPγ as a candidate EMT-inducing transcription factor. **A** Schematic overview of the experimental workflow. H3K4me3 ChIP-seq and RNA-seq analyses were performed using A549 cells with or without TGF-β-treatment. Candidate EMT-inducing transcription factors (EMT-TFs) were selected from genes within the top 5% of TGF-β-expanded H3K4me3 domains and those showing 0.5 log2-fold induction in RNA-seq. **B** qRT-PCR (left) and immunoblotting (right) analyses of CDH1/E-cadherin expression in A549 cells expressing an empty vector or each of the top seven TF candidates. DDX5 (qRT-PCR) and GAPDH (immunoblotting) were used as internal controls. **C** Phenotypic characterization of A549 cells expressing C/EBPγ. (Upper panels) Bright-field images showing morphological changes. Scale bar, 200 μm. (Middle panels) Immunofluorescence images of E-cadherin localization. Scale bar, 50 μm. (Lower panels) Actin cytoskeleton visualized by TRITC-phalloidin staining. Scale bar, 50 μm. **D** Gene set enrichment analysis (GSEA) showing enrichment of the hallmark EMT gene set (upper), TGF-β-induced EMT upregulated genes (middle), and TGF-β-induced EMT downregulated genes (lower) in C/EBPγ-overexpressing cells. Enrichment statistics: EMT (ES = 0.63, NES = 2.13, *P* = 0), TGF-β-EMT-up (ES = 0.84, NES = 3.08, *P* = 0), TGF-β-EMT-down (ES = −0.83, NES = −2.86, *P* = 0). Adjusted *P*-values were calculated using the Benjamini–Hochberg procedure. **E**–**H** qRT-PCR analysis of CDH2/N-cadherin, VIM/Vimentin, SNAI2, and ZEB2 expression in A549 cells expressing an empty vector or C/EBPγ. *P*-values were calculated by Student’s *t*-test using normalized values before scaling to 1 (*P < 0.05; **P < 0.01; ***P < 0.001 versus control).

To uncover previously unrecognized transcription factors contributing to EMT, we performed an epigenomic analysis of histone H3K4me3 distribution during TGF-β–induced EMT in LUAD cells (Schema in Fig. 1A). Because broad H3K4me3 domains often mark key regulators of cell identity [13, 14], we searched for transcription factors whose H3K4me3 domains expanded upon EMT induction, focusing on the genes in which H3K4me3 domains expanded from transcription start sites toward gene bodies as reported [16, 17]. Among the top 5% of genes showing the broader H3K4me3 domain expansion after TGF-β treatment, we identified multiple classical EMT markers (e.g., VIM) and core EMT-TFs (e.g., SNAI1, SNAI2) (Supplementary Table S1). Consistently, enrichment analysis demonstrated that the hallmark EMT gene set was significantly overrepresented within this group (Supplementary Fig. 1A). To prioritize candidate transcription factors within the top 5% of H3K4me3-expanded genes, we intersected the top 5% H3K4me3-expanded genes with TGF-β–induced transcription factors in our RNA-seq dataset (Supplementary Table S2) and excluded minimally induced candidates (log₂ fold change <0.5) (Supplementary Fig. 1B). This filtering strategy enabled us to narrow the list to transcription factors that exhibit both substantial H3K4me3 domain expansion and appreciable TGF-β–induced expression. These top 7 transcription factor candidates are *SKIL*, *ETS2*, *ZNF710*, *ELK3*, *SNAI1*, *VGLL3* and *C/EBPγ*, of which *SKIL*, *ETS2*, *ELK3*, *SNAI1* and *VGLL3* are reported to be associated with EMT [18–23]. To evaluate the EMT-inducing potential of these seven factors, we then overexpressed each in A549 cells and examined the expression of *CDH1*/E-cadherin, a hallmark of epithelial identity, as a primary EMT readout. As shown in Fig. 1B, overexpression of *ETS2*, *ZNF710*, *SNAI1*, *VGLL3* and *C/EBPγ* reduced E-cadherin mRNA and protein levels.

### Ectopic *C/EBPγ* induced EMT phenotypes and gene expression signature in LUAD cells

Since C/EBPγ had not previously been implicated in EMT, yet its overexpression most prominently reduced E-cadherin mRNA among the seven transcription factors examined (Fig. 1B), we investigated whether ectopic C/EBPγ is sufficient to induce EMT-related phenotypes. A549 cells overexpressing C/EBPγ displayed a scattered, elongated morphology, loss of membrane-localized E-cadherin, and prominent actin stress fibers, none of which were observed in control cells (Fig. 1C), indicating EMT-like morphological conversion.

To define the accompanying transcriptional changes, we performed microarray analyses to compare C/EBPγ-overexpressing and control A549 cells. Gene set enrichment analysis (GSEA) revealed significant enrichment of EMT-associated gene sets in C/EBPγ-overexpressing cells (Fig. 1D). Notably, differentially expressed genes substantially overlapped with both the top 500 TGF-β–induced and bottom 500 TGF-β–repressed genes identified in our RNA-seq dataset (TGFB-EMT_UP and TGFB-EMT_DOWN, respectively; Fig. 1D). We next validated representative EMT genes by qRT-PCR in A549 cells, confirming the upregulation of CDH2/N-cadherin, VIM/Vimentin, SNAI2, and ZEB2 upon C/EBPγ overexpression (Fig. 1E–H). Similar induction of these EMT markers was observed in NCI-H358 cells (Supplementary Fig. 1C–F), supporting the generality of this response across LUAD cell lines. Together, these results indicate that C/EBPγ activates an EMT-associated transcriptional program that recapitulates a subset of the TGF-β–induced gene expression profile in LUAD cells.

To investigate whether H3K4me3 enrichment contributes to C/EBPγ induction and EMT phenotype, we reduced global H3K4me3 levels using the H3K4 methyltransferase inhibitors MM-102 and BCI-121 (Supplementary Fig. 1G). Both inhibitors attenuated the TGF-β-induced C/EBPγ expression (Supplementary Fig. 1H), while also preventing E-cadherin suppression and actin fiber formation (Supplementary Fig. 1I). These findings indicate that H3K4me3 enrichment is required for efficient activation of both C/EBPγ and EMT in response to TGF-β.

### *C/EBPγ* knockdown impairs EMT progression during TGF-β treatment

**Fig. 2:**
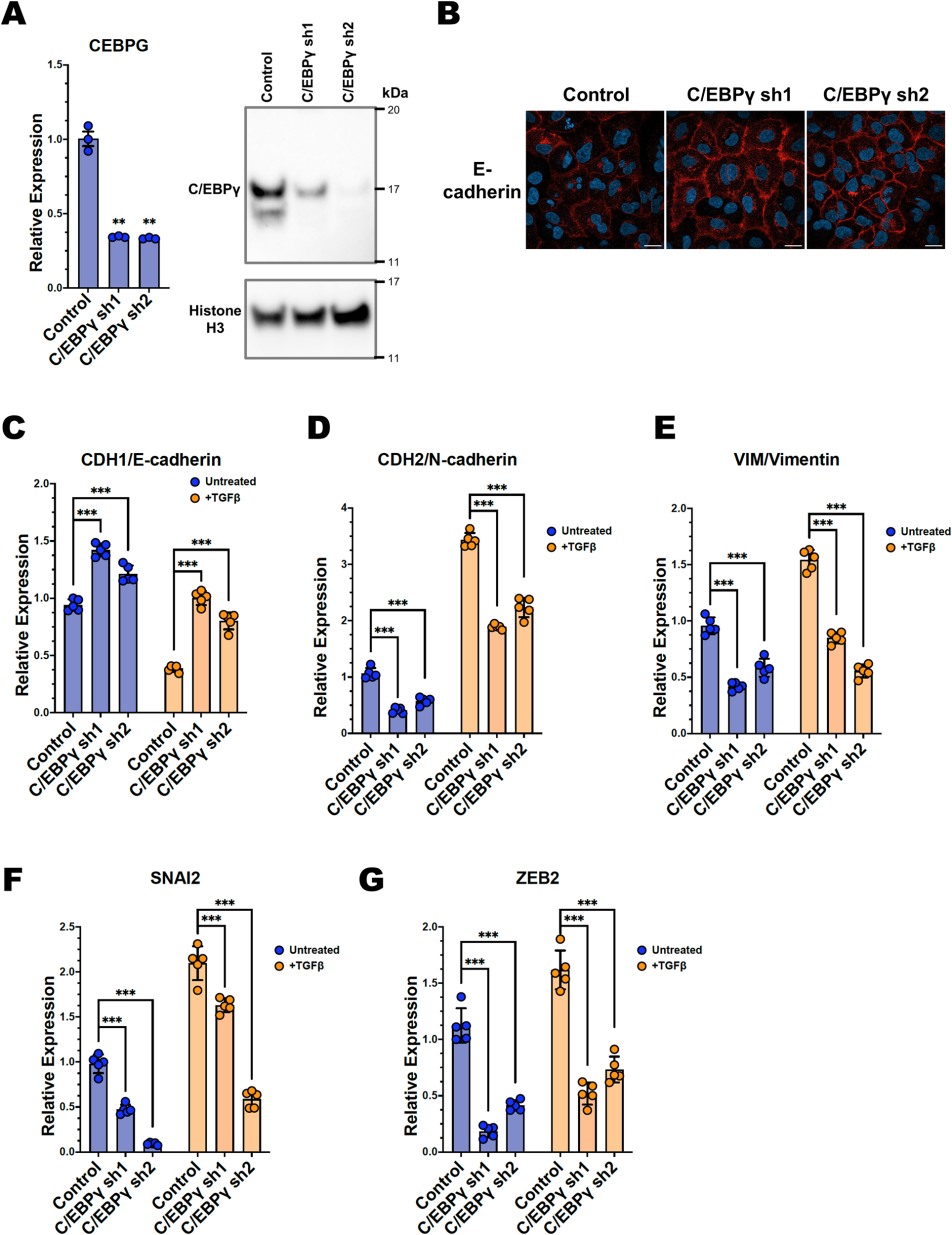
Knockdown of C/EBPγ suppresses EMT marker expression in both TGF-β–treated and untreated LUAD cells. **A** Downregulation of C/EBPγ using C/EBPγ shRNAs was evaluated in both mRNA and protein levels. Histone H3 was used as an internal control of immunoblot analysis. **B** Fluorescence images showing increased E-cadherin immunostaining in C/EBPγ-knockdown A549 cells. Scale bar represents 50 μm. qRT-PCR analysis was performed to measure the expression of CDH1/E-cadherin (**C**), CDH2/N-cadherin (**D**), VIM/Vimentin (**E**), SNAI2 (**F**), and ZEB2 (**G**) in A549 cells infected with lentiviruses expressing control shRNA or C/EBPγ shRNAs, with or without treatment with 1 ng/ml TGF-β for 24 h. DDX5 was used as internal controls. Each sample was compared to its corresponding control by Student’s *t*-test using normalized values before scaling to 1 (*P < 0.05; **P < 0.01; ***P < 0.001 versus control).

To define the functional contribution of endogenous C/EBPγ to EMT, we downregulated C/EBPγ expression by shRNA in A549 and NCI-H358 LUAD cells (Fig. 2A, and Supplementary Fig. 2A) and examined its impact on EMT-associated gene expression. In A549 cells, C/EBPγ knockdown enhanced E-cadherin staining (Fig. 2B). Consistently, CDH1/E-cadherin expression was increased, whereas mesenchymal markers, including CDH2/N-cadherin, VIM/Vimentin and SNAI2 were reduced in both cell lines. ZEB2 was reduced in A549 cells and was barely detectable in NCI-H358 cells (Fig. 2C–G, and Supplementary Fig. 2B–E). These transcriptional alterations were accompanied by corresponding protein-level changes in A549 cells, with elevated E-cadherin and decreased Vimentin (Supplementary Fig. 2F).

We next assessed whether endogenous C/EBPγ is required for the EMT-associated cell motility induced by TGF-β. Notably, C/EBPγ knockdown attenuated TGF-β–mediated CDH1/E-cadherin repression and induction of mesenchymal markers in both cell lines (Fig. 2C–G, and Supplementary Fig. 2B–E). Consistent with the diminished EMT gene response, C/EBPγ knockdown significantly impaired TGF-β–induced migratory capacity in A549 cells (Supplementary Fig. 2G).

Collectively, these findings demonstrate that endogenous C/EBPγ is required for efficient EMT-associated marker switching and acquisition of motile properties during TGF-β–induced EMT.

### C/EBPγ induced EMT phenotypes via its leucine zipper domain

**Fig. 3:**
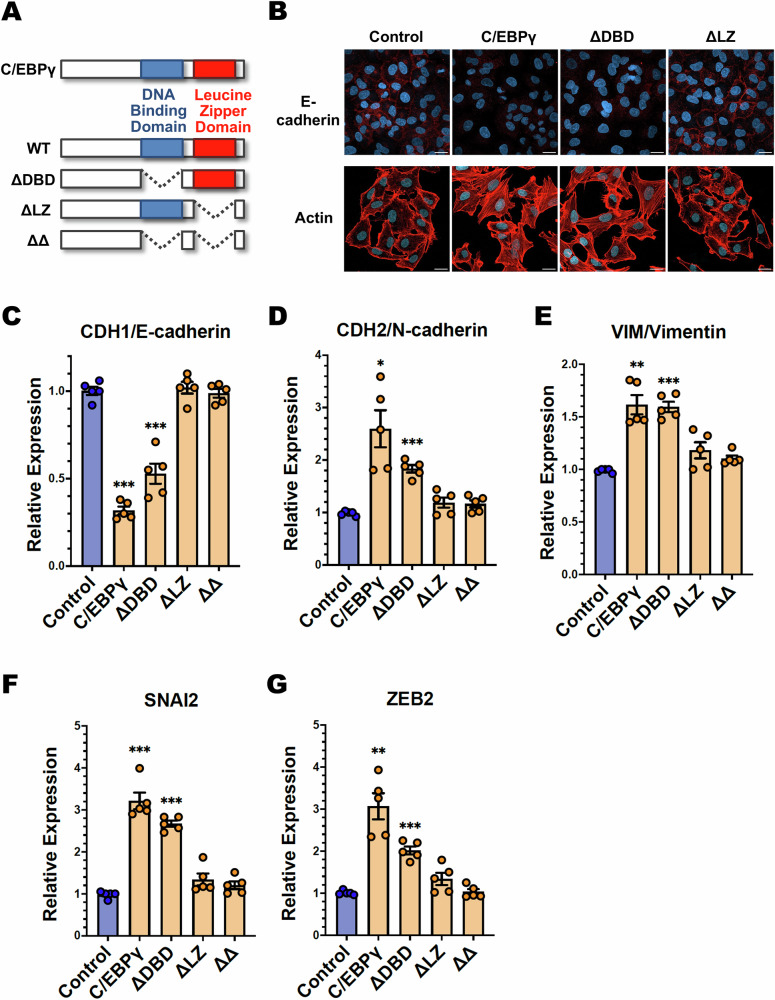
C/EBPγ regulates EMT marker gene expression through its LZ domain rather than its DNA-binding domain. **A** Schematic representation of C/EBPγ deletion mutants. **B** Fluorescence images showing reduced E-cadherin immunostaining signals and prominent actin stress fiber formation in A549 cells expressing wild-type C/EBPγ or the ΔDBD mutant, whereas these mesenchymal features were not observed in cells expressing the ΔLZ mutant. Scale bar, 50 μm. qRT-PCR analysis of CDH1/E-cadherin (**C**), CDH2/N-cadherin (**D**), VIM/Vimentin (**E**), SNAI2 (**F**), and ZEB2 (**G**) in A549 cells infected with control retrovirus or retroviruses expressing wild-type C/EBPγ, C/EBPγ ΔDBD, C/EBPγ ΔLZ, or C/EBPγ ΔΔ. For the C/EBPγ-overexpressing conditions in **D**–**G**, the same qPCR data as those shown in Fig. 1E–H were used. DDX5 was used as an internal control. Each sample was compared to its corresponding control by Student’s *t*-test using normalized values before scaling to 1 (*P < 0.05; **P < 0.01; ***P < 0.001 versus control).

To map the C/EBPγ domain required for EMT induction, we generated deletion mutants lacking the DNA-binding domain (ΔDBD), the leucine zipper domain (ΔLZ), or both (ΔΔ), and expressed them in A549 cells (Fig. 3A). All constructs were expressed at comparable levels, indicating preserved protein stability (Supplementary Fig. 3A).

Expression of wild-type C/EBPγ induced characteristic features of mesenchymal transition, including diminished E-cadherin immunofluorescence and enhanced actin stress fiber formation (Fig. 3B). Notably, a similar phenotype was observed with the ΔDBD mutant, whereas neither change was detected with the ΔLZ mutant, indicating a critical role for the LZ domain in EMT-associated morphology. Accordingly, ectopic expression of full-length C/EBPγ suppressed CDH1/E-cadherin expression, an effect retained by the ΔDBD mutant but abolished by deletion of the LZ in either the ΔLZ or ΔΔ construct (Fig. 3C). Similarly, full-length C/EBPγ robustly induced mesenchymal markers, including CDH2/N-cadherin, VIM/Vimentin, SNAI2, and ZEB2; this activity was completely lost upon LZ deletion but remained intact in the ΔDBD mutant (Fig. 3D–G). These transcriptional outcomes were recapitulated at the protein level for CDH1 and Vimentin (Supplementary Fig. 3B). In migration assays, wild-type and ΔDBD increased motility, whereas ΔLZ failed to do (Supplementary Fig. 3C).

Together, these data demonstrate that the LZ domain, but not the DNA-binding domain, is essential for C/EBPγ-mediated induction of EMT markers, mesenchymal morphology, and motility, implicating an LZ-dependent, partner-mediated mechanism of EMT activation.

### C/EBPγ interacts with C/EBPβ and the NHEJ factors XRCC5 and XRCC6

**Fig. 4:**
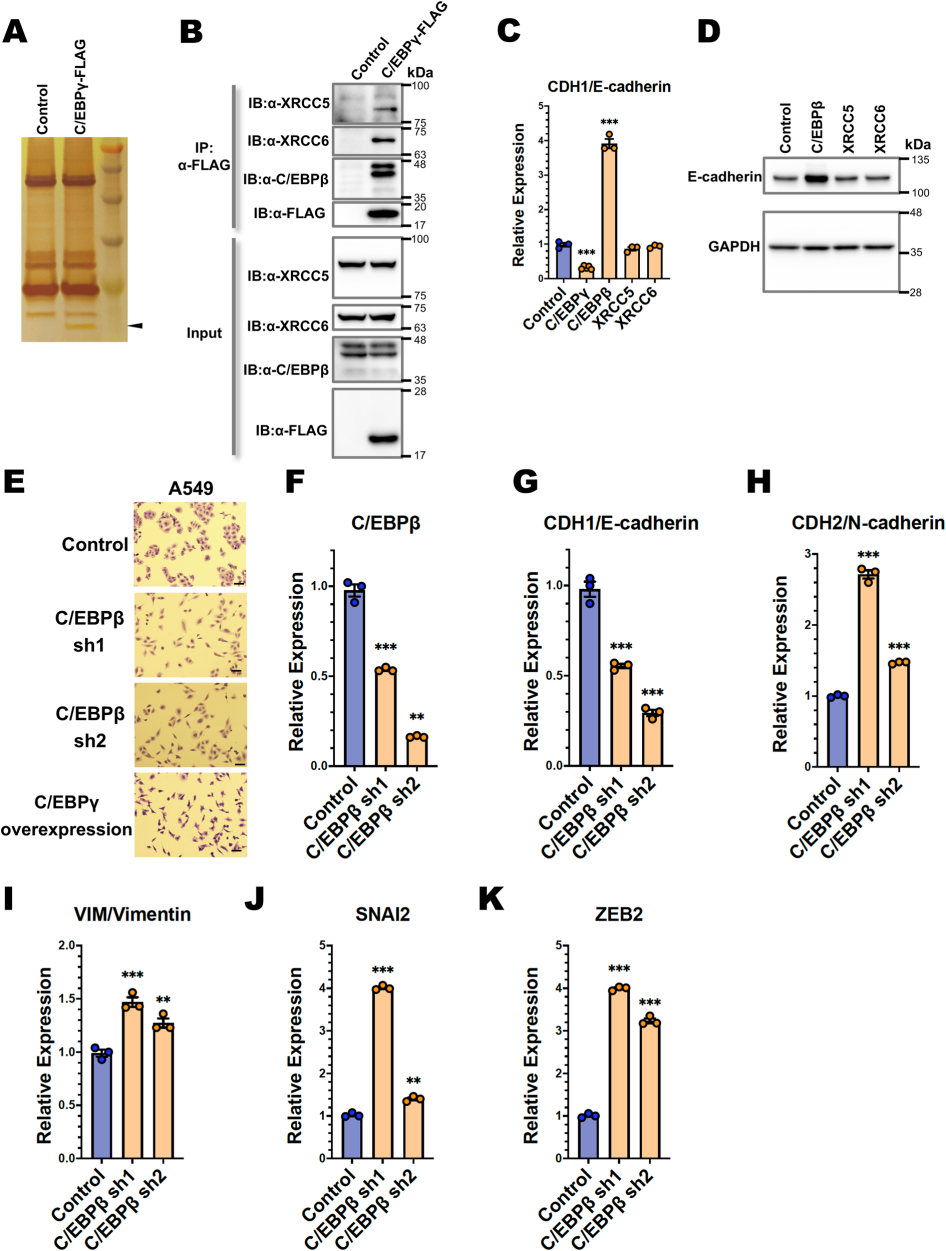
C/EBPγ interacts with C/EBPβ and the NHEJ factors XRCC5/6, and C/EBPβ negatively regulates EMT. **A** Silver staining of FLAG-immunoprecipitated samples. The position of C/EBPγ-FLAG is indicated by an arrow. **B** Co-immunoprecipitation of endogenous XRCC5, XRCC6, and C/EBPβ with C/EBPγ-FLAG, confirmed by immunoblotting using specific antibodies. **C**, **D** qRT-PCR and immunoblot analyses of CDH1 (E-cadherin) expression in A549 cells infected with control retrovirus or retrovirus expressing C/EBPβ, XRCC5, or XRCC6. DDX5 (qRT-PCR) and GAPDH (immunoblot) were used as internal control genes. **E** Representative morphological changes in A549 cells infected with lentivirus expressing control shRNA or C/EBPβ shRNAs, or with a retrovirus expressing C/EBPγ. C/EBPγ-overexpressing cells were used as a positive control for mesenchymal-type cell morphology in A549 cells. Scale bar, 200 μm. **F**–**K** qRT-PCR analysis of CDH2 (N-cadherin), VIM (Vimentin), SNAI2, and ZEB2 expression in A549 cells infected with control shRNA or C/EBPβ shRNAs. DDX5 was used as an internal control gene. Each sample was compared to its corresponding control by Student’s *t*-test using normalized values before scaling to 1 (*P < 0.05; **P < 0.01; ***P < 0.001 versus control).

Because the LZ domain of C/EBPγ mediates interactions with partner proteins, we sought to identify C/EBPγ-associated proteins in A549 cells using a proteomics-based approach. Lysates from A549 cells expressing FLAG-tagged wild-type C/EBPγ (C/EBPγ-FLAG) were subjected to anti-FLAG immunoprecipitation (Fig. 4A), and co-immunoprecipitated endogenous proteins were analyzed by mass spectrometry. Proteins enriched in the C/EBPγ–FLAG immunoprecipitate are listed in Table 1 (Number of unique detected peptides ≥3; C/EBPγ–FLAG/Control protein quantity ratio ≥20), with an expanded dataset provided in Supplementary Table S3 (Number of unique detected peptides ≥2; C/EBPγ–FLAG/Control protein quantity ratio ≥5). C/EBPγ itself exhibited the highest peptide coverage, validating the specificity and robustness of the pulldown.

Given that C/EBPβ is a known C/EBPγ dimerization partner, we prioritized this interaction, and we also focused on XRCC5 and XRCC6, which ranked among the most abundant interactors and function as core components of the non-homologous end-joining (NHEJ) machinery. Notably, both XRCC5 and XRCC6 were recovered at higher levels than C/EBPβ in the proteomic dataset.

To confirm these interactions, we performed co-immunoprecipitation followed by immunoblotting using antibodies against C/EBPβ, XRCC5, and XRCC6. All three proteins co-immunoprecipitated with C/EBPγ–FLAG (Fig. 4B), substantiating the mass spectrometry findings.

These results demonstrate that C/EBPγ forms complexes with C/EBPβ and the NHEJ factors XRCC5 and XRCC6 in A549 cells, implicating these interactions as potential mediators of C/EBPγ function.

### C/EBPγ functioned to suppress the EMT-inhibiting effects of C/EBPβ

To identify which C/EBPγ-interacting proteins involved in EMT regulation, we overexpressed C/EBPβ, XRCC5, and XRCC6 in A549 cells and evaluated E-cadherin expression (Supplementary Fig. 4A). C/EBPβ overexpression increased CDH1/E-cadherin, whereas XRCC5 and XRCC6 had no detectable effect (Fig. 4C, D). Consistently, the expression of mesenchymal markers (CDH2, VIM, SNAI2, and ZEB2) was decreased, supporting the possibility that C/EBPβ negatively regulates EMT (Supplementary Fig. 4B–E).

To assess whether endogenous C/EBPβ restrains EMT, we knocked down C/EBPβ expression in A549 cells. C/EBPβ knockdown cells exhibited scattered, elongated morphology similar to that observed in C/EBPγ-overexpressing cells (Fig. 4E) and showed decreased CDH1 alongside increased CDH2, VIM, SNAI2, and ZEB2 expression (Fig. 4F–K). Similar transcriptional changes occurred in NCI-H358 cells (Supplementary Fig. 4F–J). These data demonstrate that C/EBPβ functions as an EMT suppressor in LUAD cells and suggest that C/EBPγ promotes EMT by antagonizing C/EBPβ.

**Fig. 5:**
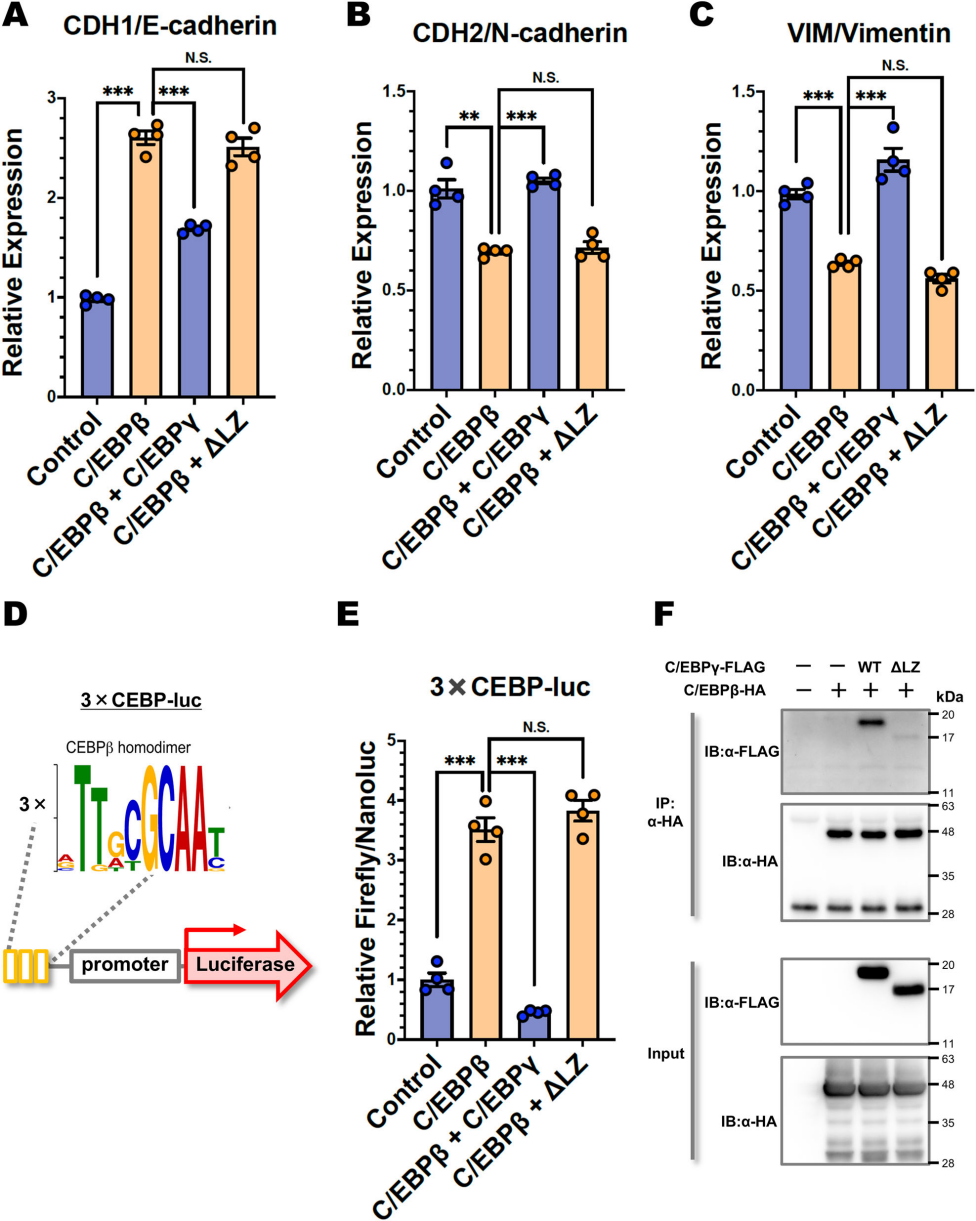
C/EBPγ counteracts the inhibitory effects of C/EBPβ on C/EBPγ-regulated EMT marker genes through its LZ domain. **A**–**C** qRT-PCR analysis of CDH1 (E-cadherin), CDH2 (N-cadherin), and VIM (Vimentin) expression in A549 cells infected with control retrovirus or a retrovirus expressing C/EBPβ, or co-infected with retroviruses expressing C/EBPβ and either C/EBPγ or its ΔLZ mutant. DDX5 was used as an internal control gene. **D** Schematic representation of the 3×C/EBP-luc reporter construct. **E** Luciferase reporter activity of the 3×C/EBP-luc reporter in HEK293T cells transfected with the indicated plasmids. **F** Co-immunoprecipitation analysis of C/EBPγ–C/EBPβ interaction. HEK293T cells were transfected with the indicated plasmids. Top and middle panels: immunoprecipitation of C/EBPβ-HA using anti-HA antibody followed by immunoblotting with the indicated antibodies. Bottom panels: input levels in whole-cell lysates. For **A**–**C** and **E**, statistical significance was determined by one-way ANOVA followed by Dunnett’s post-hoc test for comparisons against C/EBPβ-expressing cells (*P < 0.05, **P < 0.01, ***P < 0.001; N.S., not significant).

We next tested whether C/EBPγ suppresses C/EBPβ activity in an LZ-dependent manner. Full-length C/EBPγ, but not the ΔLZ mutant, inhibited C/EBPβ-induced CDH1 expression (Fig. 5A) and attenuated the expression of additional EMT markers modulated by C/EBPβ (Fig. 5B, C). To determine whether C/EBPγ antagonizes C/EBPβ at the transcriptional level, we performed luciferase assays using a 3×C/EBP reporter in HEK293T cells [24] (Fig. 5D). C/EBPβ transactivation was suppressed by C/EBPγ in an LZ domain-dependent manner (Fig. 5E, and Supplementary Fig. 5). Furthermore, co-immunoprecipitation experiments showed that C/EBPβ-HA co-precipitated with C/EBPγ–FLAG but not with C/EBPγ ΔLZ–FLAG (Fig. 5F), confirming that the LZ domain is required for their interaction.

We further investigated whether C/EBPβ could counteract the EMT phenotypes induced by C/EBPγ overexpression. Co-expression of C/EBPβ with C/EBPγ abrogated the C/EBPγ-mediated suppression of CDH1 expression and the upregulation of mesenchymal markers (Supplementary Fig. 6A–C). Consistently, C/EBPβ expression reversed the C/EBPγ-induced changes in CDH1 and VIM protein levels (Supplementary Fig. 6D). In line with these molecular findings, co-expression of C/EBPβ attenuated the mesenchymal-like morphology induced by C/EBPγ, restored E-cadherin signal at the cell membrane, and reduced actin stress fiber formation (Supplementary Fig. 6E).

Collectively, these findings demonstrate that C/EBPγ physically interacts with and antagonizes C/EBPβ in an LZ domain-dependent manner, thereby relieving the EMT-suppressive effects of C/EBPβ and promoting EMT progression.

### C/EBPγ interacts with XRCC5 and XRCC6 through the LZ domain of C/EBPγ

**Fig. 6:**
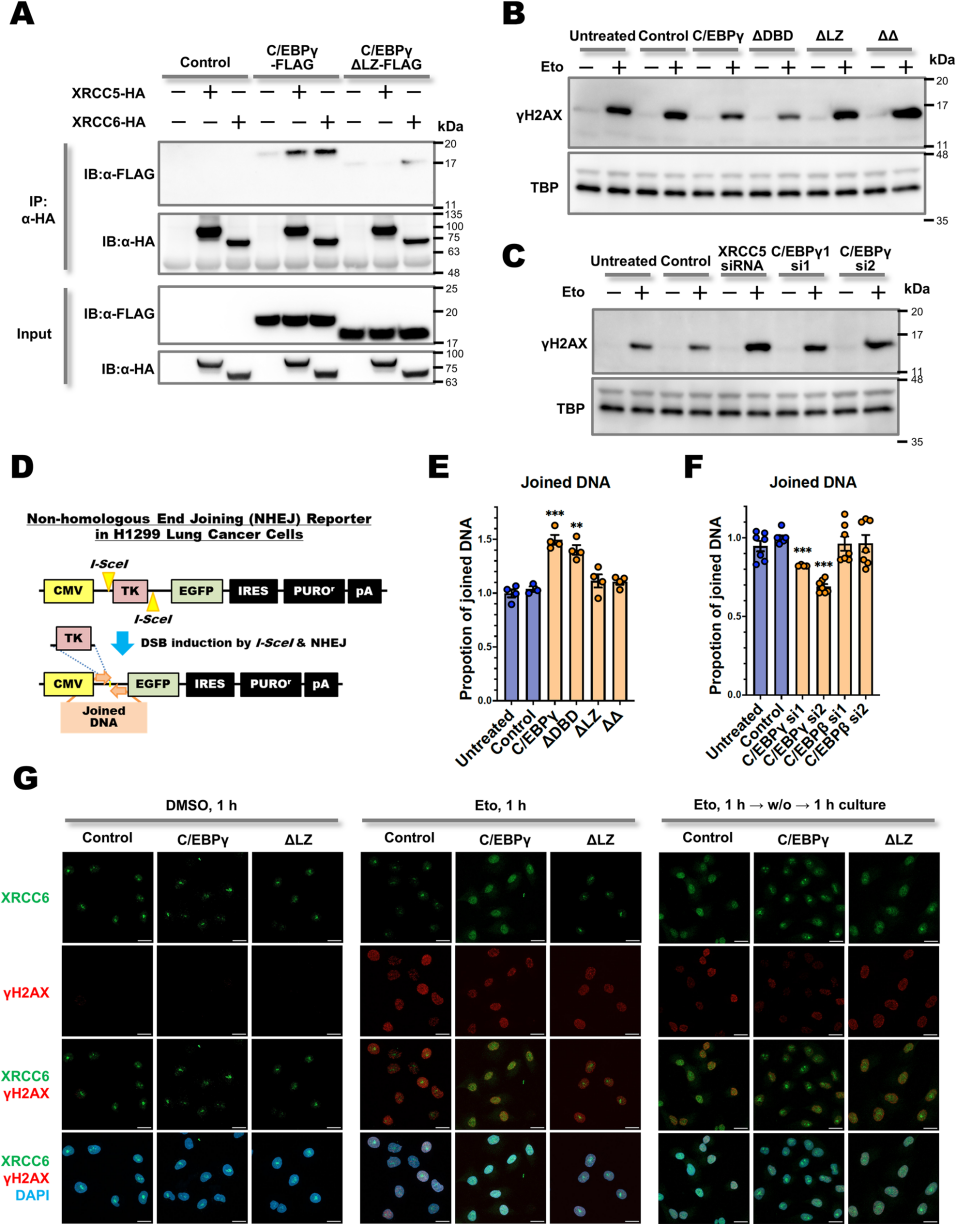
C/EBPγ enhances DNA double-strand break repair and NHEJ activity through its LZ domain. **A** Co-immunoprecipitation analysis of XRCC5-HA or XRCC6-HA with C/EBPγ-FLAG or C/EBPγ-ΔLZ-FLAG in HEK293T cells. Top two panels: immunoprecipitation (IP) with anti-HA followed by immunoblotting (IB) with the indicated antibodies. Bottom two panels: input from whole-cell lysates. **B** Western blot analysis of γ-H2AX in A549 cells infected with control retrovirus or retroviruses expressing C/EBPγ, C/EBPγ-ΔDBD, C/EBPγ-ΔLZ, or C/EBPγ-ΔΔ. Cells were treated with 25 μM etoposide and harvested 48 h later. TBP was used as an internal control. **C** Western blot analysis of γ-H2AX in A549 cells transfected with control siRNA, XRCC5 siRNA, or C/EBPγ siRNAs. Cells were treated with 25 μM etoposide for 48 h. TBP was used as an internal control. **D** Schematic of the NHEJ reporter assay. Two I-SceI recognition sites (yellow arrowheads) were engineered to generate a defined double-strand breaks (DSBs) upon I-SceI expression. Orange arrows indicate primer locations used to quantify re-joined DNA by qPCR. CMV, cytomegalovirus promoter/enhancer; IRES, internal ribosome entry site; pA, polyadenylation signal. **E** qPCR analysis of joined DNA in H1299dA3-1 reporter cells infected with control retrovirus or retroviruses expressing C/EBPγ, C/EBPγ-ΔDBD, C/EBPγ-ΔLZ, or C/EBPγ-ΔΔ. GAPDH DNA was quantified as an internal control. **F** qPCR analysis of joined DNA in H1299dA3-1 cells transfected with control siRNA, C/EBPγ siRNAs, or C/EBPβ siRNAs. GAPDH DNA was quantified as an internal control. **G** Immunofluorescence images of XRCC6 and γH2AX in A549 cells infected with control retrovirus or retrovirus expressing C/EBPγ or C/EBPγ-ΔLZ. Cells were treated with DMSO for 1 h (left), 25 μM etoposide (Eto) for 1 h (middle), or 25 μM etoposide for 1 h followed by washout and an additional 1 h culture period (right). Scale bar, 15 μm. For **E**, **F**, each sample was compared to its concurrent control by Student’s *t*-test using normalized values before scaling to 1 (*P < 0.05; **P < 0.01; ***P < 0.001 versus control).

Since XRCC5 and XRCC6 were among the proteins co-immunoprecipitated with C/EBPγ and are key components of NHEJ, we tested whether their association requires the C/EBPγ LZ domain. HEK293T cells were co-transfected with C/EBPγ–FLAG or C/EBPγ ΔLZ–FLAG together with XRCC5–HA or XRCC6–HA, followed by co-immunoprecipitation (Fig. 6A). XRCC5–HA and XRCC6–HA pulled down full-length C/EBPγ–FLAG but not the ΔLZ mutant, demonstrating LZ-dependent binding and suggesting a link between C/EBPγ and NHEJ-mediated DSB repair.

### C/EBPγ improves DSB repair efficiency through its LZ domain

Recent studies have suggested that EMT-TFs often contribute to cancer malignancy not only through EMT regulation but also by engaging additional pathways involved in genome maintenance and stress adaptation [25]. In light of these emerging concepts, we next examined whether C/EBPγ plays a functional role in NHEJ-mediated DSB repair. To evaluate this, we treated A549 cells overexpressing C/EBPγ with etoposide, a topoisomerase II inhibitor that induces DNA DSBs, and assessed γ-H2AX levels, a marker of DSBs [26]. Etoposide-induced γ-H2AX accumulation was markedly reduced in cells expressing full-length C/EBPγ compared with controls, indicating enhanced repair efficiency (Fig. 6B). A similar reduction was observed in cells expressing the ΔDBD mutant. In contrast, γ-H2AX levels remained unchanged relative to controls in ΔLZ- or ΔΔ-expressing cells, demonstrating that the LZ domain is essential for C/EBPγ-mediated enhancement of repair.

Conversely, C/EBPγ knockdown by siRNAs or shRNAs increased etoposide-induced γ-H2AX (Fig. 6C, and Supplementary Fig. 7A), consistent with impaired DSB repair. XRCC5 knockdown, which disrupts NHEJ, served as a positive control for DSB accumulation.

### C/EBPγ contributes to activating NHEJ in LUAD cells

The identification of XRCC5 and XRCC6 as C/EBPγ-interacting partners suggested that C/EBPγ may participate in NHEJ, the principal pathway for DSB repair. We therefore quantified NHEJ activity using the H1299dA3 LUAD reporter line, which carries a stably integrated NHEJ reporter containing two I-SceI recognition sites (ref. [27] and Fig. 6D). Overexpression of full-length C/EBPγ significantly increased the proportion of joined DNA, and this enhancement persisted in cells expressing the ΔDBD mutant. In contrast, neither the ΔLZ nor ΔΔ mutants increased NHEJ activity (Fig. 6E). Conversely, C/EBPγ knockdown by siRNAs or shRNAs reduced NHEJ-mediated joining, while C/EBPβ knockdown had no measurable effect (Fig. 6F, and Supplementary Fig. 7B). This divergence indicates that C/EBPγ uniquely enhances NHEJ activity in LUAD cells through its LZ domain and that this function may not rely on heterodimeric interaction with C/EBPβ.

### C/EBPγ facilitates timely XRCC6/Ku recruitment to DSB sites in an LZ domain-dependent manner

Since C/EBPγ physically interacts with the Ku complex (XRCC5/6) through its LZ domain, we hypothesized that this association might facilitate the early recruitment of Ku complex to DNA damage sites, thereby promoting NHEJ. To test this possibility, we performed immunocytochemical analysis of XRCC6, which serves as a representative indicator of Ku complex recruitment [28], to evaluate its localization to DSB sites in A549 cells following etoposide treatment. Previous studies have shown that XRCC6 accumulates in nucleoli under basal conditions and re-localizes to DSB sites following DNA damage induction [29, 30]. Consistent with these reports, XRCC6 displayed a punctate nuclear localization pattern in control, wild-type C/EBPγ-overexpressing, and ΔLZ-expressing cells, with no apparent differences under basal conditions (Fig. 6G, left panel). After 1 h of etoposide treatment, however, wild-type C/EBPγ expression significantly increased the colocalization of XRCC6 with γH2AX-positive DSB foci, accompanied by a clear redistribution of XRCC6 from its basal punctate nuclear pattern to DNA damage-associated foci (Fig. 6G, middle panel). In contrast, this change was much less evident in control cells and in cells expressing the ΔLZ mutant (Fig. 6G, middle panel; Supplementary Fig. 8A). These findings indicate that wild-type C/EBPγ accelerates the early recruitment of XRCC6, and thus presumably the Ku complex, to etoposide-induced DSB sites in an LZ-dependent manner. Notably, despite this microscopy-based difference at the early time point, immunoblot analysis showed no appreciable difference in total γH2AX levels among the three groups after 1 h of etoposide exposure (Supplementary Fig. 8B).

When etoposide was washed out, and cells were cultured for an additional 1 h, γH2AX-positive foci were already less abundant in wild-type C/EBPγ-expressing cells than in control cells or ΔLZ-expressing cells (Fig. 6G, right panel). Consistent with this finding, XRCC6 returned toward its basal punctate nuclear localization pattern in wild-type C/EBPγ-expressing cells. By contrast, in control and ΔLZ-expressing cells, prominent XRCC6 colocalization with γH2AX-positive foci became clearly detectable at this stage, indicating that recruitment of XRCC6 to DSB sites was delayed relative to wild-type C/EBPγ-expressing cells (Fig. 6G, right panel). In agreement with the immunofluorescence results, γH2AX levels were reduced after washout in wild-type C/EBPγ-expressing cells, whereas total XRCC5 and XRCC6 protein levels remained largely unchanged (Supplementary Fig. 8B). These observations suggest that wild-type C/EBPγ-expressing cells appear to complete DSB repair earlier than control cells or cells expressing the ΔLZ mutant.

Together, these findings demonstrate that C/EBPγ may promote DSB repair in an LZ domain-dependent manner, at least in part by facilitating the timely recruitment of XRCC6/Ku to DNA damage sites.

### C/EBPγ increases the chemoresistance of A549 cells under etoposide treatment

**Fig. 7:**
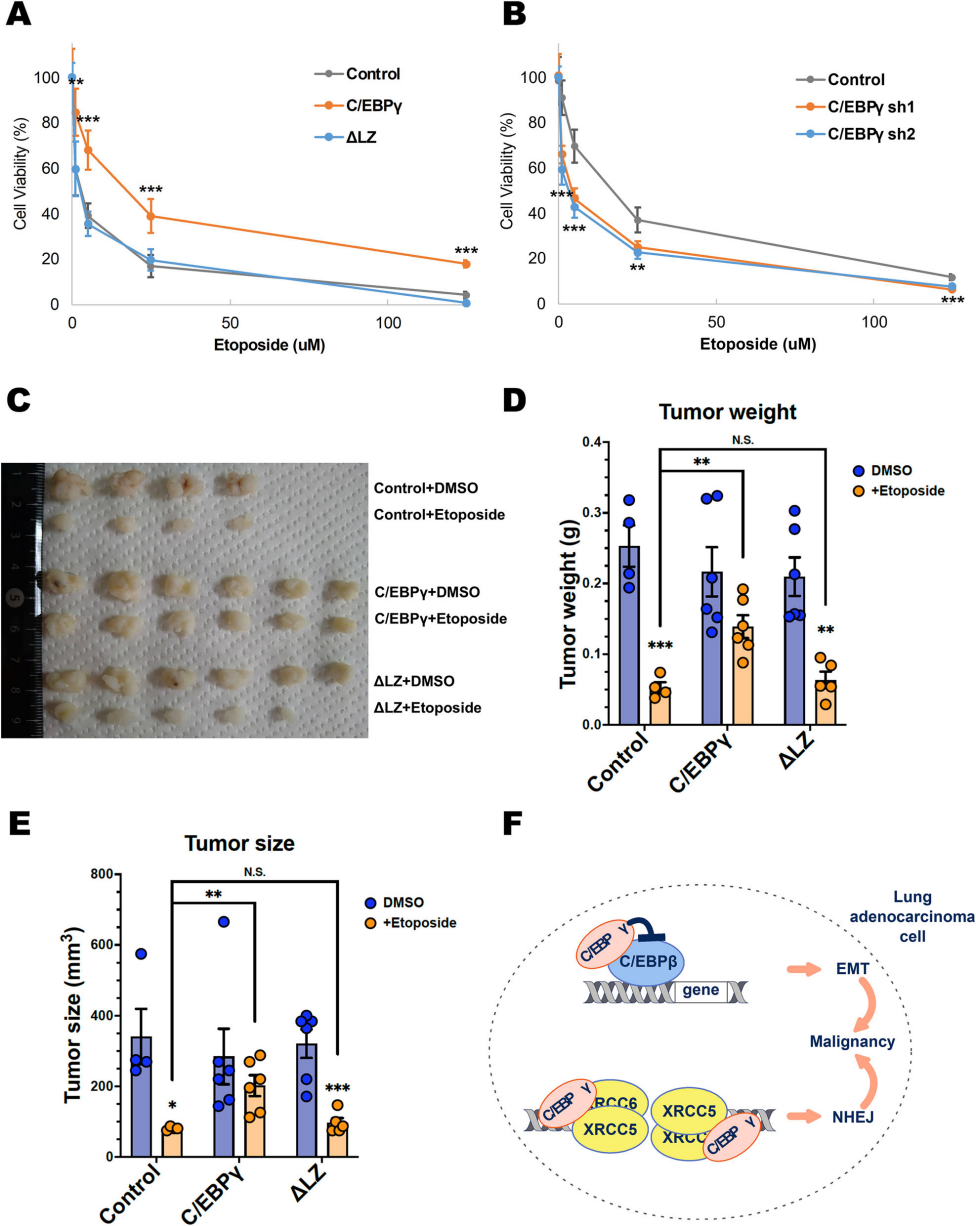
C/EBPγ contributes to cell viability under etoposide treatment. **A** CCK-8 assays of A549 cells infected with control retrovirus or retrovirus expressing C/EBPγ or C/EBPγ ΔLZ. Cells were treated with the indicated concentrations of etoposide for 72 h. **B** CCK-8 assays of A549 cells infected with lentiviruses expressing control shRNA or C/EBPγ shRNAs. Cells were treated with the indicated concentrations of etoposide for 72 h. **C**–**E** In vivo xenograft assays evaluating the effect of C/EBPγ on tumor response to etoposide. A total of 5 × 10⁶ A549 cells expressing control vector, C/EBPγ, or C/EBPγ ΔLZ were subcutaneously injected into female BALB/c-nu/nu mice (*n* ≥ 4 per group). Once tumors became palpable, mice were treated with etoposide (10 mg/kg) or DMSO by intraperitoneal injection for five consecutive days. Tumors were excised ten days after the final injection. **C** Representative images of tumors from each group. **D** Tumor weights and **E** tumor volumes calculated using the formula *V* = (*L* × W²)/2. C/EBPγ overexpression, but not C/EBPγ ΔLZ, increased tumor growth under etoposide treatment. **F** Schematic model illustrating how C/EBPγ promotes the EMT phenotype and enhances DNA double-strand break repair, thereby contributing to increased malignancy in LUAD cells. For **A**, **B**, each sample was compared with its concurrent control by Student’s *t*-test on normalized values before scaling to 1. For **D**, **E**, statistical significance was determined by Student’s *t*-test for pairwise comparisons and by one-way ANOVA followed by Dunnett’s post-hoc test for comparisons against the etoposide-treated control (*P < 0.05, **P < 0.01, ***P < 0.001 vs. control).

Given that etoposide exerts antitumor activity through the induction of DSBs, we next examined whether C/EBPγ influences LUAD cell survival under etoposide exposure. CCK-8 assays revealed that C/EBPγ overexpression increased A549 cell viability following etoposide treatment, whereas the ΔLZ mutant failed to do so (Fig. 7A), indicating an LZ-dependent protective effect. Consistently, C/EBPγ knockdown reduced the viability of etoposide-treated cells (Fig. 7B).

To assess whether the LZ-dependent survival advantage conferred by C/EBPγ translates into therapeutic resistance in vivo, in the context of enhanced DSB repair through interaction with XRCC5 and XRCC6, we performed xenograft assays using A549 cells with or without C/EBPγ overexpression. Following etoposide treatment, tumors derived from cells expressing full-length C/EBPγ displayed significantly greater tumor weight and volume than controls, whereas ΔLZ-expressing tumors did not (Fig. 7C–E), demonstrating that C/EBPγ promotes etoposide resistance in vivo in an LZ-dependent manner.

Collectively, these results indicate that C/EBPγ enhances LUAD cell survival under DSB-inducing chemotherapy by activating NHEJ and conferring LZ-dependent resistance to etoposide.

## Discussion

In this study, we reveal a novel function of C/EBPγ in linking epithelial–mesenchymal plasticity to enhanced DNA DSB repair capacity in LUAD cells. Mechanistically, C/EBPγ promotes EMT by functionally antagonizing the EMT-suppressive activity of C/EBPβ, while independently enhancing NHEJ through interaction with the Ku complex components XRCC5 and XRCC6. Together with the observed increase in cellular fitness under etoposide-induced genotoxic stress, these findings support a model in which C/EBPγ coordinates EMT-associated transcriptional reprogramming with DNA repair proficiency (Fig. 7F).

Clinically, elevated C/EBPγ expression is associated with poor outcome in LUAD (Supplementary Fig. 9), and C/EBPγ has been reported to be upregulated in AT2-like cell populations emerging during atypical adenomatous hyperplasia and progression toward stem-like transcriptional states [31]. These observations are consistent with a role for C/EBPγ in malignant progression, although they remain correlative and will require functional validation in patient-derived and in vivo settings.

A unique methodological aspect of our study is the use of H3K4me3 domain broadening as an epigenomic feature to select candidate EMT regulators. Our epigenomic analysis revealed that C/EBPγ is marked by broad H3K4me3 domains during TGF-β–induced EMT. H3K4me3 broad domains often highlight transcription factors that are central to cell identity transitions, suggesting that broadened domains may signify regulatory importance rather than simply transcriptional upregulation. Previous approaches to identifying EMT-TFs have largely relied on screening for E-cadherin repressors or factors highly expressed in mesenchymal states. In contrast, our strategy captures transcriptional regulators whose epigenomic features change during EMT, enabling the discovery of candidates that might be overlooked by expression-based approaches alone. The identification of C/EBPγ underscores the utility of this framework and may encourage broader applications of epigenomic signatures in defining lineage plasticity regulators.

Next, functionally, we show that C/EBPγ is sufficient to induce EMT-like morphological and molecular changes in LUAD cells and is required for complete EMT progression during TGF-β stimulation. Importantly, domain mapping revealed that the LZ domain, but not the canonical DNA-binding domain, is indispensable for EMT marker modulation. This requirement places protein–protein interaction at the center of C/EBPγ function to drive EMT. Given that C/EBPγ preferentially engages in heterodimeric interactions through its LZ domain [32, 33], defining the composition and context-dependence of C/EBPγ-containing complexes will be critical for understanding how it rewires EMT programs in LUAD.

One key consequence of this interaction-based mode of action is functional antagonism between C/EBPγ and C/EBPβ. C/EBPβ overexpression increased CDH1/E-cadherin and attenuated mesenchymal marker induction, whereas C/EBPβ knockdown elicited EMT-like phenotypes and an EMT gene signature, consistent with a tumor-suppressive role for C/EBPβ in this context. C/EBPγ suppressed C/EBPβ-driven transcriptional activity in an LZ domain-dependent manner, supporting a model in which C/EBPγ restrains C/EBPβ activity via heteromeric complex formation and thereby relieves a brake on EMT. The rescue experiments (Supplementary Fig. 6), in which enforced C/EBPβ expression reversed the molecular and morphological EMT phenotypes induced by C/EBPγ, further strengthen this antagonistic model. This mechanism is consistent with prior observations that loss of C/EBPβ can bias TGF-β responses toward EMT [34], and it extends the C/EBPβ–C/EBPγ axis to LUAD as a regulatory node controlling epithelial–mesenchymal state transitions.

In parallel with its EMT-promoting function, we provide evidence that C/EBPγ enhances DSB repair through NHEJ. C/EBPγ interacts with XRCC5 and XRCC6 in an LZ domain-dependent manner and increases NHEJ reporter-based joining efficiency, accompanied by reduced γ-H2AX accumulation following etoposide treatment. Conversely, C/EBPγ knockdown compromises NHEJ-mediated joining. Notably, C/EBPβ knockdown had no detectable effect on NHEJ in the same assay system, arguing that the NHEJ-promoting activity of C/EBPγ is unlikely to be mediated indirectly through C/EBPβ-containing complexes and instead reflects a separable C/EBPγ–Ku axis. While previous studies have shown that certain EMT-TFs can influence DNA repair pathways [35, 36], their mechanisms differ considerably: ZEB1 promotes NHEJ via interaction with 53BP1 [37], whereas SNAI1 suppresses NHEJ by inhibiting DNA-PKcs activity [38], and SNAI2 has been implicated in homologous recombination [39]. Our findings add C/EBPγ to this emerging group but highlight a mechanistically distinct mode of action involving its bZIP domain. Our data (Fig. 6G) support a model in which C/EBPγ potentiates NHEJ, at least in part, by facilitating the early recruitment of the Ku complex to DSB sites in an LZ-dependent manner; however, whether C/EBPγ also promotes subsequent steps in NHEJ, such as Ku retention at DNA ends, DNA-PK activation, or the establishment of a repair-competent chromatin environment, remains an important question for future work.

The integration of EMT and DNA repair regulation may have important implications for tumor evolution. EMT is increasingly recognized not only as a driver of metastasis but also as a modulator of therapy resistance [36, 40–42]. By linking EMT-promoting activity with enhanced DNA repair capacity, C/EBPγ may enable carcinoma cells to better withstand genotoxic therapies. In agreement with this possibility, C/EBPγ increased cell survival under etoposide treatment in vitro and promoted resistance to etoposide in xenograft models in an LZ domain-dependent manner. Although high C/EBPγ expression correlated with poor prognosis in LUAD patients, further investigation will be required to establish causality and to delineate the contexts in which C/EBPγ contributes to therapeutic resistance in vivo.

In summary, our study identifies C/EBPγ as a novel EMT-inducing transcription factor whose activities are mediated through its LZ domain and extend beyond EMT regulation to include the promotion of NHEJ-mediated DNA repair. These findings broaden the functional repertoire of C/EBPγ and reveal a mechanistic link between EMT-associated transcriptional reprogramming and DNA damage repair. Future work will be needed to define the broader network of LZ-dependent interactions, to clarify how C/EBPγ modulates NHEJ machinery at the molecular level, and to determine whether targeting C/EBPγ or its interaction interfaces may enhance responses to DNA-damaging therapies. In this light, a deeper mechanistic dissection of C/EBPγ may help define regulatory principles by which epithelial–mesenchymal plasticity is coordinated with DNA repair capacity, with potential implications for cancer therapy.

## Materials and methods

### Cell culture and transfections

The A549 (ATCC) and the NCI-H358 (ATCC) human LUAD cell lines were used as previously described [10, 43, 44]. EMT was induced in A549 cells by exposure to 1 ng/ml TGF-β (R&D Systems, Minneapolis, MN, USA) for 24 h. In NCI-H358 cells, EMT induction was achieved by serum starvation (0.5% FBS) and treatment with 1 ng/ml TGF-β for 3 weeks. To assess the contribution of H3K4me3 enrichment to TGF-β–induced EMT, A549 cells were treated with the H3K4 methyltransferase inhibitors MM-102 and BCI-121 (Selleck Chemicals, Houston, TX, USA). Cells were pretreated with either MM-102 (10 μM) or BCI-121 (10 μM) for 48 h to inhibit global H3K4me3 enrichment, followed by stimulation with 1 ng/ml TGF-β for 24 h. After treatment, global H3K4me3 levels, C/EBPγ expression, E-cadherin levels at the cell membrane and actin stress fiber formation were examined. For the induction of DNA DSBs, A549 cells were treated with 25 μM etoposide (Fujifilm Wako Pure Chemical, Osaka, Japan). The H1299dA3-1 cell line, which is used to monitor NHEJ activity in LUAD cells [27], was kindly provided by Dr. A. Ui (Tohoku University, Japan) and Dr. T. Kohno (National Cancer Center Research Institute, Japan). The methods used for generating and transducing retroviral vectors expressing shRNA or cDNA were performed as previously described [44–46]. All cell lines were routinely tested and verified to be free of mycoplasma contamination.

### siRNA knockdown

Cells were transfected with predesigned Silencer® Select siRNAs (Ambion, Waltham, MA, USA) targeting C/EBPβ (siC/EBPβ1, ID s2891; siC/EBPβ2, ID s2893), C/EBPγ (siC/EBPγ1, ID s2900; siC/EBPγ2, ID s2901), and XRCC5 (siXRCC5, ID s14954) using Lipofectamine RNAiMAX (Invitrogen, Waltham, MA, USA) in accordance with the manufacturer’s instructions. Silencer® Select Negative Control #1 siRNA was used as a negative control in all experiments.

### Plasmids

Lentiviral vectors expressing small hairpin RNAs (shRNAs) were generated following previously described methods [10]. The oligonucleotide sequences used for shRNA synthesis are listed in Supplementary Table S4. For cloning human cDNAs of *SKIL*, *ETS2*, *ZNF710*, *ELK3*, *SNAI1*, *VGLL3*, *C/EBPγ*, *XRCC5*, *XRCC6*, and *C/EBPβ*, primer sets based on a prior report [46] and listed separately in Supplementary Table S4 were utilized. The amplified FLAG-tagged cDNAs of *SKIL*, *ETS2*, *ZNF710*, *ELK3*, *SNAI1*, *VGLL3*, and *C/EBPγ* were inserted into the pDON-5 Neo plasmid (Takara, Ohtsu, Japan) to create retroviral vectors expressing these proteins. The pMXs-puro plasmid, a generous gift from Dr. C. Takahashi (Kanazawa University, Japan), was used to construct retroviral vectors expressing HA-tagged *XRCC5*, *XRCC6*, and *C/EBPβ* according to a previously published protocol [44]. The C/EBP-luc reporter was generated by inserting annealed 3×CEBP-luc oligonucleotides into the pGL4.25 plasmid (Promega, Madison, WI, USA), following the methodology outlined in the referenced work [24]. The *I-Sce* I endonuclease expression plasmid, used for NHEJ reporter assays, was kindly provided by Dr. A. Ui and Dr. T. Kohno.

### Quantitative PCR

Total RNA extraction and quantitative reverse transcription PCR (RT-qPCR) were carried out following previously established methods [44]. Quantitative PCR results were normalized to the expression levels of an internal control gene (*GAPDH* or *DDX5*). Data represent the mean ± standard deviation from at least three independent experiments. Statistical significance between the control and experimental groups was evaluated using Student’s *t*-test. The primers used for RT-qPCR are listed in Supplementary Table S4 and were based on sequences reported previously [10, 12, 44].

### Cell staining, immunofluorescence, and immunoblotting

To examine changes in cell morphology, A549 cells were stained with 0.4% crystal violet. For visualization of the actin cytoskeleton, cells were treated with 0.25 μM TRITC-conjugated phalloidin (Sigma, Burlington, MA, USA). Indirect immunofluorescence was performed using anti-E-cadherin antibody (610181, BD, San Jose, CA, USA) followed by incubation with Alexa Fluor 546-conjugated secondary anti-mouse IgG antibody (Invitrogen, Waltham, MA, USA). Nuclei were counterstained with 4′,6-diamidino-2-phenylindole (DAPI). To evaluate XRCC6 recruitment to DNA DSB sites, cells were permeabilized and fixed as previously described [47]. XRCC6 and the DSB marker γH2AX were detected using anti-XRCC6 (ab83501, Abcam, Cambridge, MA, USA) and anti-γH2AX (05-636, Millipore, MA, USA) antibodies, respectively. Colocalization of XRCC6 with γH2AX foci was quantified using the Coloc 2 plugin in Fiji software. Immunoblotting was carried out following previously described protocols [10]. The antibodies used included anti-E-cadherin, anti-histone H3K4me3 (39159, Active Motif, Carlsbad, CA, USA), anti-C/EBPγ (GTX32514, GeneTex, CA, USA), anti-XRCC5 (sc-5280, Santa Cruz, CA, USA), anti-XRCC6 (sc-5309, Santa Cruz, CA, USA), anti-C/EBPβ (sc-7962, Santa Cruz, CA, USA), anti-Vimentin (ab8069, Abcam, Cambridge, MA, USA), anti-γH2AX (ab81299, Abcam, Cambridge, MA, USA), anti-GAPDH (6C5, Millipore, MA, USA), anti-histone H3 (MABI0301, MBL, Nagoya, Japan), and anti-TBP (22006-1-AP, Proteintech, Rosemont, IL, USA). To detect endogenous C/EBPγ in LUAD cells, nuclear extracts were prepared as outlined in a previous study [48], and samples were subjected to SDS-PAGE on 15−20% tricine gels (Fujifilm Wako Pure Chemical, Osaka, Japan).

### Microarray

Total RNA was extracted using RNAiso Plus reagent (Takara, Kyoto, Japan), and its quality was assessed using an Agilent 4200 TapeStation (Agilent Technologies, Santa Clara, CA, USA). All samples exhibited RNA Integrity Numbers (RIN) greater than 8.9 and were subjected to microarray analysis following the manufacturer’s instructions. In brief, 200 ng of total RNA was labeled with cyanine 3-CTP using the Low Input Quick Amp Labeling Kit (Agilent Technologies, Santa Clara, CA, USA). Hybridization was performed with the Gene Expression Hybridization Kit (Agilent Technologies, Santa Clara, CA, USA). A total of 1.65 µg of complementary RNA (cRNA) per sample was fragmented at 60 °C for 30 min and hybridized to the Whole Human Genome Microarray 4x44K Ver. 2.0 (G4845A, Agilent Technologies, Santa Clara, CA, USA) in a rotary oven at 65 °C for 17 h. After hybridization, the slides were washed using Agilent Gene Expression Wash Buffers 1 and 2 (Agilent Technologies, Santa Clara, CA, USA) and immediately scanned using the Agilent DNA Microarray Scanner (G2539A) with the one-color scan setting for 1 × 44k array slides. The scanned images were processed using Feature Extraction Software 11.0.1.1 (Agilent Technologies, Santa Clara, CA, USA) to obtain background-subtracted and spatially detrended processed signal intensities. To ensure consistency across arrays, gene expression values were normalized using GeneSpring software 14 (Agilent Technologies, Santa Clara, CA, USA). The microarray data have been deposited in the Gene Expression Omnibus (GEO) database of the NCBI under the accession number GSE288216.

### ChIP-seq

ChIP assays were performed following protocols described in earlier reports [10, 12, 44]. Cross-linked chromatin was immunoprecipitated using an anti-histone H3K4me3 antibody (39159, Active Motif, Carlsbad, CA, USA). For ChIP-seq library preparation, the NEBNext ChIP-seq Library Prep Master Mix Set for Illumina (New England Bio Labs, Ipswich, USA) was used according to the manufacturer’s guidelines, and libraries were subsequently sequenced on an Illumina HiSeq 2500 platform. The sequencing reads were aligned to the human reference genome (hg19) using Bowtie2, and peak calling was performed with MACS2 applying the broad option. The ChIP-seq data have been deposited in the NCBI Gene Expression Omnibus (GEO) under accession number GSE285671.

### RNA-seq

Total RNA was isolated using RNA iso Plus reagent (Takara, Kyoto, Japan), and its integrity was evaluated with the BioAnalyzer 2100 (Agilent Technologies, Santa Clara, CA, USA). RNA-seq libraries were prepared using the TruSeq Stranded mRNA LT Sample Prep Kit (Illumina, California, CA, USA) and 150 bp single-end reads were generated. The resulting data were uniformly analyzed following previously established protocols [49]. Differential gene expression analysis was performed using the DESeq2 package with an lfcThreshold of 0.1. Gene expression levels were quantified as FPKM values using commonly accepted methods. The RNA-seq data have been submitted to the NCBI Gene Expression Omnibus (GEO) under the accession number GSE285672.

### IP-MS and LAIP-mass spectrometry (MS)

Pellets were resuspended in 6M urea and 50 mM TEAB (pH 8.5). Protein samples were adjusted to a final volume of 10 μl, followed by reduction with 5 mM TCEP at 37 °C for 30 min in the dark and alkylation with 24 mM iodoacetamide for 30 min at room temperature. Proteins were digested with trypsin (Mass spectrometry grade, Promega, Madison, WI, USA) at a 1:10 enzyme-to-protein ratio for 16 h at 37 °C. Peptides were desalted using StageTips (#84850, Thermo Pierce, Rockford, IL, USA) according to the manufacturer’s instructions and eluted with 70% acetonitrile. The eluted peptides were vacuum-dried and resuspended in 5% ACN with 0.1% trifluoroacetic acid. The resulting peptides were analyzed using an Orbitrap QE Plus (Thermo Fisher Scientific, Waltham, MA, USA) coupled to a nano-liquid chromatography system (EASY-nLC 1200; Thermo Fisher Scientific, Waltham, MA, USA). Peptide separation was performed using a 25 cm × 75 μm ID, 1.6 μm C18 column (IonOpticks) with a linear acetonitrile gradient (0–35%) in 0.1% formic acid at 300 nL/min. Mass spectra were acquired using Orbitrap QE Plus in data-dependent acquisition mode, controlled by Xcalibur software (Thermo Fisher Scientific, Waltham, MA, USA). Full-scan MS spectra were obtained over an *m*/*z* range of 375–1500 at a resolution of 70,000. The raw MS data have been deposited in the Japan ProteOme Standard Repository (https://repository.jpostdb.org/) with accession numbers PXD061625 (ProteomeXchange) and JPST003685 (jPOST).

### MS data analysis

The MS/MS searches were carried out using SEQUEST HT search algorithms against the Homo sapiens (Swiss prot. Tax ID 9609) protein database (2017-9-14) using Proteome Discoverer (PD) 2.2 (Version 2.2.0.388; Thermo Fisher Scientific, Waltham, MA, USA). Label-free quantification was also performed with PD 2.2 using precursor ions quantifier nodes. The processing workflow included spectrum files RC, spectrum selector, SEQUEST HT search nodes, percolator, ptmRS, and minor feature detector nodes. Oxidation of methionine was set as a variable modification and carbamidomethylation of cysteine was set as a fixed modification. Mass tolerances in MS and MS/MS were set at 10 ppm and 0.6 Da, respectively. Trypsin was specified as protease and a maximum of two missed cleavages was allowed. Target-decoy database searches were used to calculate the false discovery rate (FDR), which was controlled at 1% at the peptide level. The consensus workflow included MSF files, Feature Mapper, precursor ion quantifier, PSM grouper, peptide validator, peptide and protein filter, protein scorer, protein marker, protein FDR validator, protein grouping, peptide in protein. Normalization of the abundances was performed using total peptide amount mode. Protein mass spectrometry data was deposited to Japan ProteOme Standard Repository. Identified proteins are listed in Table 1 and Supplementary Table S3.

**Table 1:** C/EBPγ-associated proteins identified by FLAG immunoprecipitation followed by mass spectrometry.

Protein Identification Confidence Based on *q*-value (Column D) high<0.01, 0.01<medium<0.05, 0.05<low			*q*-value for Protein Identification	Cumulative Score of Each Peptide	Coverage Rate	Number of Detected Peptides	Total Number of Detected Peptides (Including Redundant and Distinct Charge States)	Number of Unique Detected Peptides								Protein Quantity Ratio		Protein Abundance (F1) - Normalized	Protein Abundance (F2) - Normalized	Raw Protein Abundance Data (F1)	Raw Protein Abundance Data (F2)	Protein Detection (F1)	Protein Detection (F2)
Protein FDR Confidence: Combined	Accession	Description	Exp. *q*-value: Combined	Sum PEP Score	Coverage [%]	# Peptides	# PSMs	# Unique Peptides	# Protein Groups	# AAs	MW [kDa]	calc. pI	Score Sequest HT: Sequest HT	# Peptides (by Search Engine): Sequest HT	# Razor Peptides	Abundance Ratio: (Sample) / (Control)	Abundance Ratio Variability [%]: (Sample) / (Control)	Abundances (Grouped): Control	Abundances (Grouped): Sample	Abundance: F1: Control	Abundance: F2: Sample	Found in Sample: [S1] F1: Control	Found in Sample: [S2] F2: Sample
High	P53567	CCAAT/enhancer-binding protein gamma [CEBPγ]	0	42.409	59	10	39	**10**	1	150	16.4	9.77	101.01	10	0	**100**	157.55	1.2	198.8	36203324.7	3999528147	Peak Found	High
High	P13010	X-ray repair cross-complementing protein 5 [XRCC5]	0	26.328	19	10	10	**10**	1	732	82.7	5.81	24.17	10	0	**100**			200		4497288.45	Not Found	High
High	P12956	X-ray repair cross-complementing protein 6 [XRCC6]	0	15.577	12	7	7	**7**	1	609	69.8	6.64	13.57	7	0	**21.81**	0	0.8	199.2	26017.7266	4380285.63	Peak Found	High
High	P17676	CCAAT/enhancer-binding protein beta [CEBPβ]	0	6.591	13	3	3	**3**	1	345	36.1	8.31	6.19	3	0	**100**			200		2076531.26	Not Found	High
High	O75531	Barrier-to-autointegration factor [BANF1]	0	10.124	51	3	3	**3**	1	89	10.1	6.09	11.91	3	0	**100**			200		6353837	Not Found	High

Proteins co-immunoprecipitated with FLAG-tagged C/EBPγ in A549 cells and identified by mass spectrometry are shown. Proteins were filtered based on high-confidence identification and enrichment criteria, specifically a number of unique detected peptides ≥ 3 and a C/EBPγ–FLAG/Control protein quantity ratio ≥ 20; these two parameters are written in bold in the table. Protein identification confidence was assessed using the combined *q*-value, classified as high (<0.01), medium (0.01–0.05), or low (>0.05). The table lists protein accession numbers, descriptions, peptide-based identification metrics (coverage, total and unique peptide counts), molecular properties, and relative protein abundance ratios between C/EBPγ–FLAG immunoprecipitates and control samples. C/EBPγ exhibited the highest peptide coverage, confirming the specificity of the immunoprecipitation.

### Immunoprecipitation analysis

Cells (either infected or transfected) were lysed in RIPA buffer (50 mM Tris-HCl, pH 7.5; 150 mM NaCl; 1 mM EDTA; 1% Nonidet P-40; 0.5% sodium deoxycholate; 0.1% SDS) containing protease inhibitors (Nacalai Tesque, Kyoto, Japan). Lysates were immunoprecipitated with anti-FLAG (DYKDDDDK) (1E6, Fujifilm Wako Pure Chemical, Osaka, Japan) or anti-HA (4B2, Fujifilm Wako Pure Chemical, Osaka, Japan) antibodies, followed by incubation with protein G-Sepharose 4 Fast Flow (GE Healthcare, South East England, UK). The precipitates were analyzed by immunoblotting using anti-HA (3F10, Roche, Basel, Switzerland) and anti-FLAG (M2, Sigma, Burlington, MA, USA) antibodies.

### NHEJ assay

As described previously [27], the *I-Sce*I expression plasmid, pCBASce, was transfected into H1299dA3-1 cells, which harbor two *I-Sce*I sites located 1.3 kb apart between the promoter and GFP, using Polyethylenimine (PEI). Cells were transfected with Silencer Select siRNAs (Ambion, Austin, TX, USA) 24 h prior to *I-Sce*I plasmid transfection using Lipofectamine RNAiMAX (Invitrogen, Waltham, MA, USA) in 6-well plates. After 48 h, cells were harvested in DNA extraction buffer, washed with PBS, and genomic DNA was purified. For quantitative PCR, 20 ng of DNA was analyzed to quantify repaired DNA products using the ViiA7 real-time system (Applied Biosystems, Foster City, CA, USA). *GAPDH* DNA was co-amplified as an internal control. The primers used are listed in Supplementary Table S4.

### Transwell migration assay

The ability of cell migration was quantified by using modified Boyden chambers consisting of Transwell membrane filter inserts (Corning Costar). Indicated number of serum-starved A549 cells suspended in 100 μl of DMEM containing 0.5% FBS were cultured in each Transwell chamber and allowed to migrate toward the underside of the membrane. The lower chamber contained DMEM with 10% FBS. At 24 h after cell seeding, non-migrated cells in Transwell chambers were wiped out, and cells attached to the lower surface of the filter were fixed and stained with 0.4% crystal violet.

### Cell viability assay

A549 cell proliferation was assessed using the Cell Counting Kit-8 (CCK-8, Dojindo, Kumamoto, Japan). Approximately 2.0 × 10³ cells in 100 μl were seeded in 96-well plates in octuplicate and incubated for 24 h. Cells were then treated according to the experimental conditions described above or in figure legends. After 72 h, 10 μl of CCK-8 reagent was added to each well and incubated at 37 °C for 1.5 h. Optical density at 450 nm was measured using a microplate reader (SH-1000, Hitachi, Japan) according to the manufacturer’s instructions.

### In vivo xenograft study

All animal experiments were approved by the institutional animal care and use committee and conducted in accordance with institutional guidelines. Female BALB/c-nu/nu nude mice (8 weeks old; *n* ≥ 4 per group) were purchased from Japan SLC, Inc. (Hamamatsu, Shizuoka, Japan) and housed under specific pathogen-free conditions. A total of 5 × 10⁶ A549 cells suspended in 100 μl of 1× PBS were subcutaneously injected into the right flank of each mouse. When tumors became palpable (approximately 3–5 mm in diameter), mice were randomly assigned to treatment groups and administered either etoposide (10 mg/kg; VP-16, Selleck) or DMSO (vehicle control) by intraperitoneal injection once daily for five consecutive days. Ten days after the final injection, mice were euthanized by isoflurane inhalation, and tumors were excised, weighed, and measured. Tumor volume was calculated using the formula *V* = (*L* × *W*²) / 2, where *L* and *W* represent the longest and shortest tumor diameters, respectively.

### Statistical analysis

Data are presented as mean ± SD from at least three independent experiments. Homogeneity of variance between groups was assessed using *F*-test or Levene’s test prior to statistical comparisons. Statistical significance was determined using Student’s *t*-test or one-way ANOVA followed by Dunnett’s post-hoc test, as appropriate, as previously described [10, 12, 44]. All samples were included in the analysis without exclusion. Sample size was not predetermined, and no randomization or blinding procedures were implemented during the experiments.

## Supplementary information

Supplementary Figures

Supplementary Table S1

Supplementary Table S2

Supplementary Table S3

Supplementary Table S4

Full and uncropped western blots data

## Data Availability

All data generated or analyzed during this study including the original uncropped western blots are available in this published article and its supplementary information files.
