# Supplementary material for: C/EBPγ induces epithelial-mesenchymal transition and facilitates DNA double-strand break repair in lung adenocarcinoma cells
Source: Cell Death Discov. 2026 Jun 2;12:324. doi: 10.1038/s41420-026-03181-0 (PMC13443842; doi:10.1038/s41420-026-03181-0)
Supplement: Supplementary file 1 [file 41420_2026_3181_MOESM1_ESM.pdf]

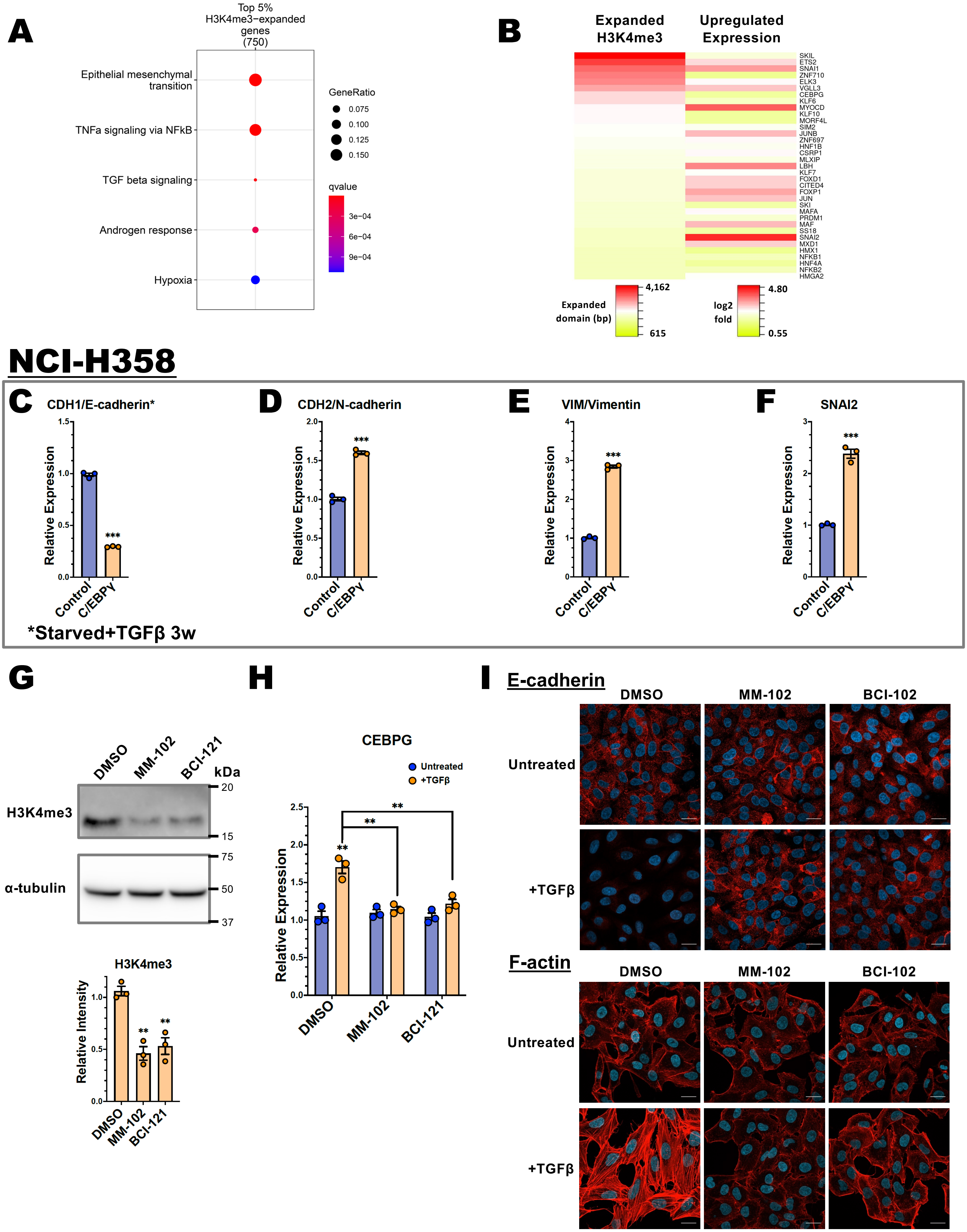

A
Top 5% H3K4me3-expanded genes (750)
Epithelial mesenchymal transition
TNFa signaling via NFkB
TGF beta signaling
Androgen response
Hypoxia
GeneRatio
0.075
0.100
0.125
0.150
qvalue
3e−04
6e−04
9e−04
B
Expanded H3K4me3
Upregulated Expression
SKIL
ETS2
SNAI1
ZNF710
ELK3
VGLL3
CEBPG
KLF6
MYOCD
KLF10
MORF4L
SIM2
JUNB
ZNF697
HNF1B
CSRP1
MLXIP
LBH
KLF7
FOXD1
CITED4
FOXP1
JUN
SKI
MAFA
PRDM1
MAF
SS18
SNAI2
MXD1
HMX1
NFKB1
HNF4A
NFKB2
HMGA2
Expanded domain (bp)
4,162
615
log2 fold
4.80
0.55
NCI-H358
C
CDH1/E-cadherin*
D
CDH2/N-cadherin
E
VIM/Vimentin
F
SNAI2
Relative Expression
Control
C/EBPγ
***
*Starved+TGFβ 3w
G
DMSO
MM-102
BCI-121
kDa
H3K4me3
α-tubulin
20
15
75
50
37
Relative Intensity
**
H
CEBPG
Untreated
+TGFβ
Relative Expression
**
I
E-cadherin
F-actin
DMSO
MM-102
BCI-102
Untreated
+TGFβ

Fig.S1

Supplementary Figure 1. TGF-β–expanded H3K4me3 domains identify C/EBPγ as a candidate EMT-inducing transcription factor.

(A) Enrichment analysis of the hallmark EMT gene set among the top 5% of genes with TGF-β–induced H3K4me3 domain expansion. (B) Distribution of H3K4me3 domain expansion (bp) and TGF-β–induced expression changes ($\log_2$ fold change) in transcription factor–encoding genes within the top 5% H3K4me3-expanded group. (C–F) qRT-PCR analysis of CDH1/E-cadherin (C), CDH2/N-cadherin (D), VIM/Vimentin (E), and SNAI2 (F) expression in NCI-H358 cells infected with retroviruses expressing empty vector or C/EBPγ. DDX5 was used as internal controls. For CDH1/E-cadherin (C), cells were cultured under low-serum conditions (0.5% FBS) and treated with 1 ng/ml TGF-β for three weeks prior to analysis, as previously described[16]. ZEB2 expression in NCI-H358 was below the level required for reliable analysis. (G) Immunoblot analysis showing the effect of the H3K4 methyltransferase inhibitors MM-102 (10 μM) and BCI-121 (10 μM) on global H3K4me3 levels in A549 cells. Cells were pretreated with each inhibitor for 48 hours, followed by TGF-β stimulation (1 ng/ml, 24 hours). α-tubulin was used as a loading control. Lower panels show densitometric quantification of H3K4me3 signals normalized to α-tubulin. (H) qRT-PCR analysis showing reduced TGF-β–induced C/EBPγ expression in MM-102– or BCI-121–pretreated cells. GAPDH was used for normalization. (I) Fluorescence images of A549 cells pretreated with H3K4 methyltransferase inhibitors (MM-102, or BCI-121) and stimulated with TGF-β. The images show increased E-cadherin immunostaining and reduced actin stress fiber formation. Scale bar, 50 μm. For (C–F, G), each sample was compared with its corresponding control using Student's t-test, as previously described (***$P < 0.001$ vs. control). For (H), statistical significance was additionally assessed by one-way ANOVA followed by Dunnett's post-hoc test for comparisons against the control (**$P < 0.01$ vs. control).

Fig.S1

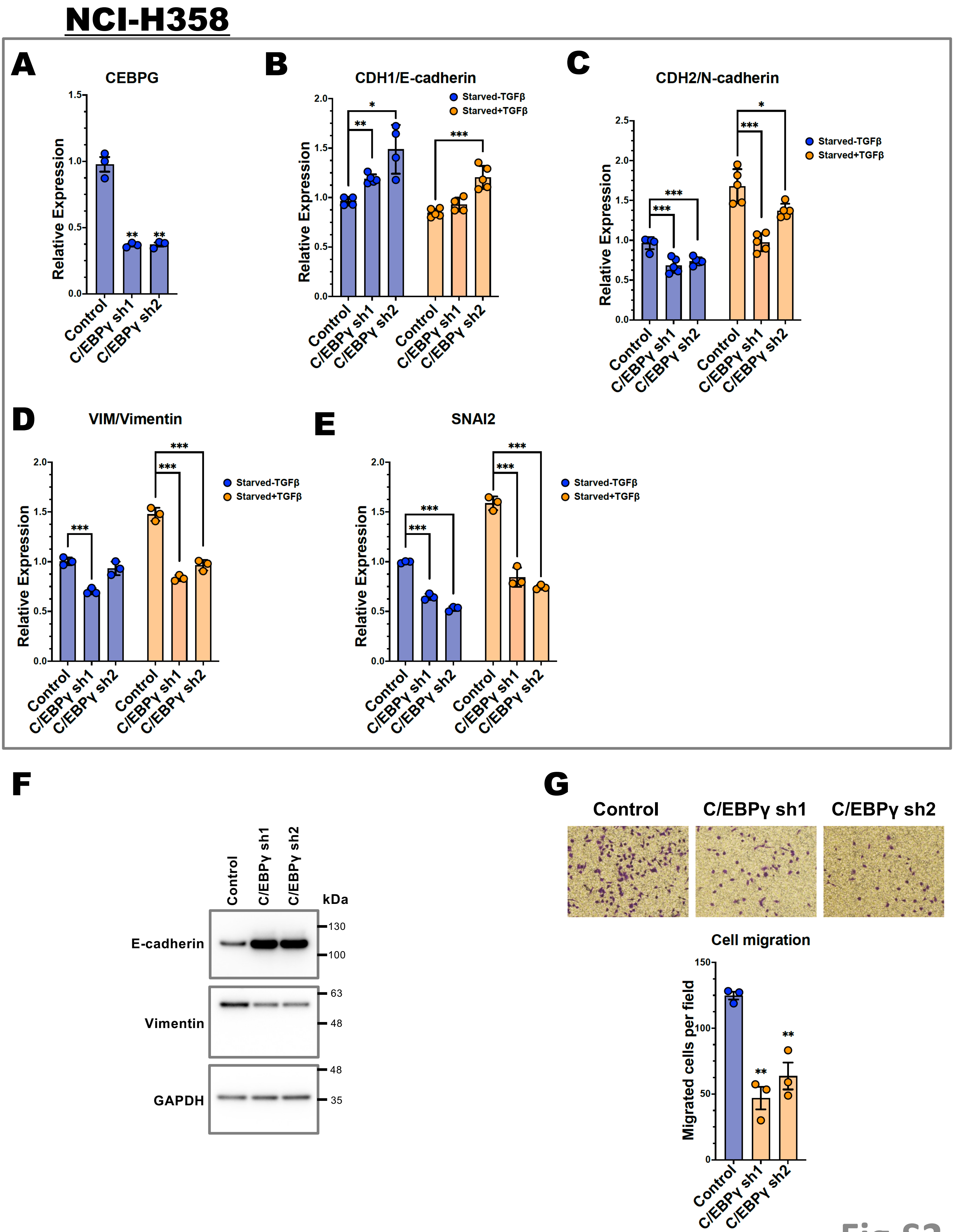
NCI-H358
A
CEBPG
Relative Expression
1.5
1.0
0.5
0.0
**
**
Control
C/EBPγ sh1
C/EBPγ sh2
B
CDH1/E-cadherin
Starved-TGFβ
Starved+TGFβ
Relative Expression
2.0
1.5
1.0
0.5
0.0
*
**
***
Control
C/EBPγ sh1
C/EBPγ sh2
Control
C/EBPγ sh1
C/EBPγ sh2
C
CDH2/N-cadherin
Starved-TGFβ
Starved+TGFβ
Relative Expression
2.5
2.0
1.5
1.0
0.5
0.0
*
***
***
***
Control
C/EBPγ sh1
C/EBPγ sh2
Control
C/EBPγ sh1
C/EBPγ sh2
D
VIM/Vimentin
Starved-TGFβ
Starved+TGFβ
Relative Expression
2.0
1.5
1.0
0.5
0.0
***
***
***
Control
C/EBPγ sh1
C/EBPγ sh2
Control
C/EBPγ sh1
C/EBPγ sh2
E
SNAI2
Starved-TGFβ
Starved+TGFβ
Relative Expression
2.0
1.5
1.0
0.5
0.0
***
***
***
***
Control
C/EBPγ sh1
C/EBPγ sh2
Control
C/EBPγ sh1
C/EBPγ sh2
F
Control
C/EBPγ sh1
C/EBPγ sh2
kDa
E-cadherin
130
100
Vimentin
63
48
GAPDH
48
35
G
Control
C/EBPγ sh1
C/EBPγ sh2
Cell migration
Migrated cells per field
150
100
50
0
**
**
Control
C/EBPγ sh1
C/EBPγ sh2

Fig.S2

Supplementary Figure 2. C/EBPγ knockdown suppresses TGF-β–dependent EMT marker expression in NCI-H358 cells and shifts cells toward an epithelial phenotype in A549 cells. qRT-PCR analysis of C/EBPγ (A), CDH1/E-cadherin (B), CDH2/N-cadherin (C), VIM/Vimentin (D), and SNAI2 (E) in NCI-H358 cells infected with lentiviruses expressing control shRNA or C/EBPγ shRNAs under starved conditions (0.5% FBS), with or without 1 ng/ml TGF-β treatment for three weeks. ZEB2 expression in NCI-H358 was below the level required for reliable analysis. DDX5 was used as internal controls. (F) Immunoblot analysis of CDH1/E-cadherin and VIM/Vimentin protein levels in A549 cells expressing control or C/EBPγ shRNAs. GAPDH was used as a loading control. (G) Transwell migration assay demonstrating reduced migratory capacity of A549 cells following C/EBPγ knockdown using C/EBPγ-targeting shRNAs. 5.0 x 10^4 serum-starved cells were spread into the chambers and fixed at 24 hours after cell seeding. For (A-E, G), each sample was compared with its corresponding control using Student's t-test (*P < 0.05; **P < 0.01; ***P < 0.001 versus control.).

Fig.S2

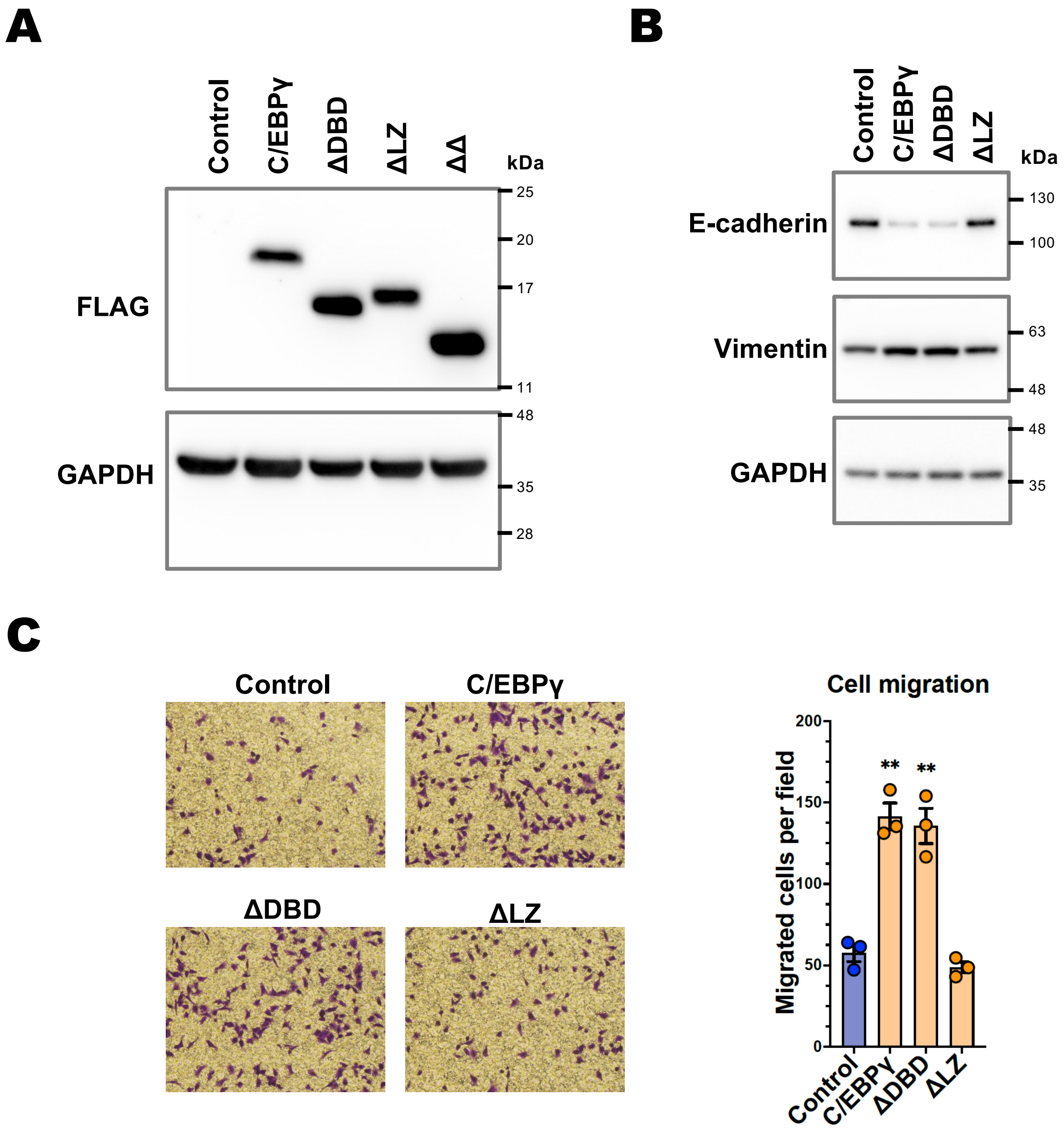
A
Control
C/EBPγ
ΔDBD
ΔLZ
ΔΔ
kDa
FLAG
GAPDH
25
20
17
11
48
35
28
B
Control
C/EBPγ
ΔDBD
ΔLZ
kDa
E-cadherin
Vimentin
GAPDH
130
100
63
48
48
35
C
Control
C/EBPγ
ΔDBD
ΔLZ
Cell migration
Migrated cells per field
200
150
100
50
0
**
**
Control
C/EBPγ
ΔDBD
ΔLZ

Fig.S3

Supplementary Figure 3. Overexpression of the LZ domain eliminates C/EBPγ-driven expressional changes of EMT marker proteins and EMT-associated phenotypes.

(A) Immunoblot analysis of FLAG-tagged wild-type C/EBPγ and the ΔDBD, ΔLZ, and ΔΔ mutants expressed in A549 cells infected with control retrovirus or retroviruses expressing each construct. GAPDH was used as internal controls. (B) Immunoblot analysis of CDH1/E-cadherin and VIM/Vimentin protein levels in A549 cells expressing FLAG-tagged wild-type C/EBPγ or the ΔDBD, ΔLZ. GAPDH was used as a loading control. (C) Transwell migration assay demonstrating enhanced migratory capacity in A549 cells expressing wild-type C/EBPγ or the ΔDBD mutant, with no increase observed in cells expressing the ΔLZ mutant. 2.5×10^4 serum-starved cells were spread into the chambers and fixed at 24 hours after cell seeding. Each sample was compared with its corresponding control using Student's t-test (**$P < 0.01$ versus control.).

Fig.S3

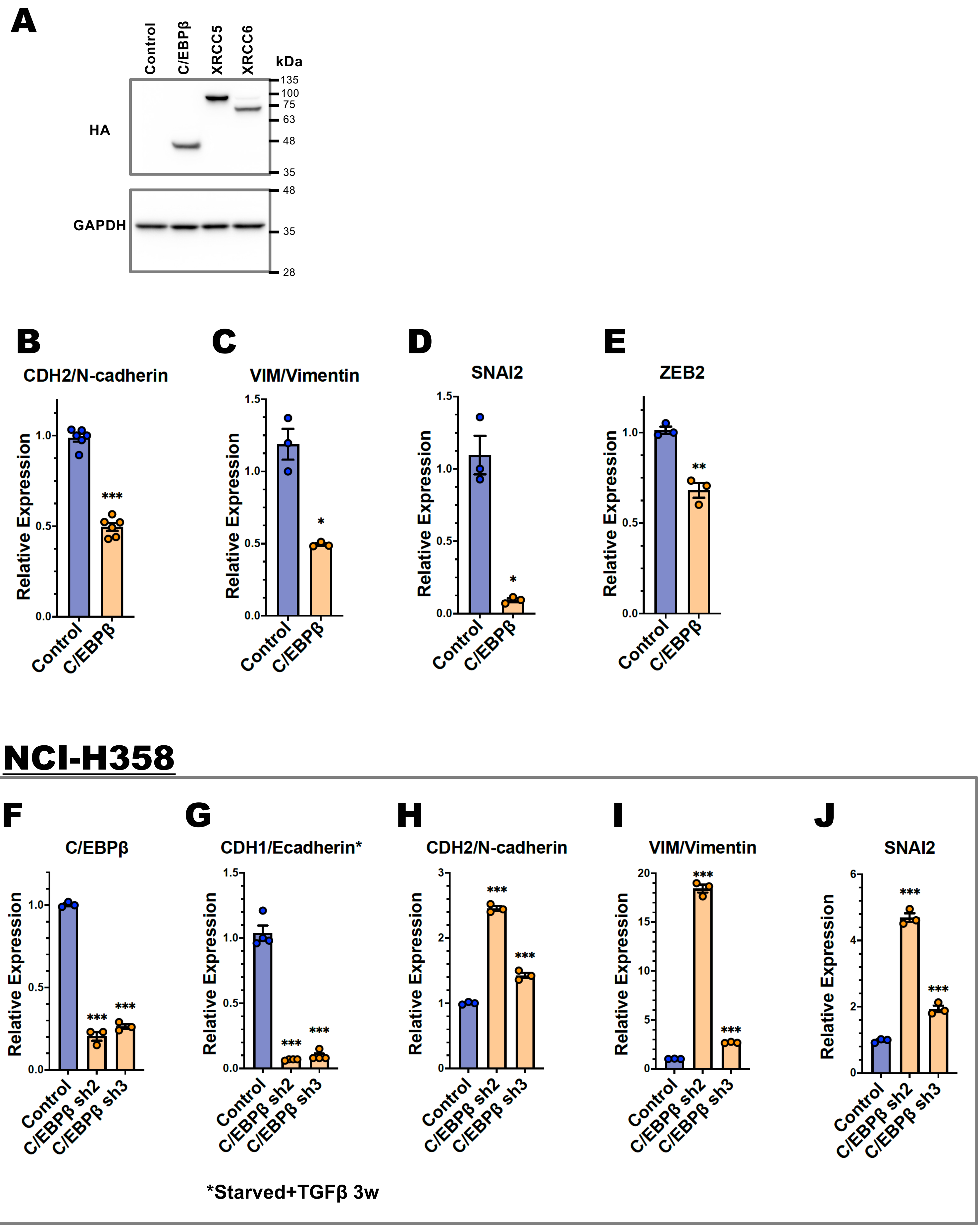
A
Control
C/EBPβ
XRCC5
XRCC6
kDa
HA
GAPDH
135
100
75
63
48
35
48
35
28
B
CDH2/N-cadherin
C
VIM/Vimentin
D
SNAI2
E
ZEB2
Relative Expression
Control
C/EBPβ
***
*
*
**
NCI-H358
F
C/EBPβ
G
CDH1/Ecadherin*
H
CDH2/N-cadherin
I
VIM/Vimentin
J
SNAI2
Control
C/EBPβ sh2
C/EBPβ sh3
*Starved+TGFβ 3w

Fig.S4

Supplementary Figure 4. Altered C/EBPβ levels affect EMT marker expression.

(A) Immunoblot analysis of HA-tagged C/EBPβ, XRCC5, and XRCC6 expressed in A549 cells. The same loading control blot as in Figure 4D was used. (B–E) Expression of CDH2 (N-cadherin), VIM (Vimentin), SNAI2, and ZEB2 in A549 cells overexpressing C/EBPβ. (F) Expression of C/EBPβ in NCI-H358 cells infected with lentiviruses expressing control shRNA or C/EBPβ shRNAs. (G–J) Expression of CDH1 (E-cadherin), CDH2 (N-cadherin), VIM (Vimentin), and SNAI2 in NCI-H358 cells infected with lentiviruses expressing control shRNA or C/EBPβ shRNAs. CDH1 (E-cadherin) expression was assessed under starvation conditions (0.5% FBS) with 1 ng/mL TGF-β treatment for three weeks, as previously described[16]. ZEB2 expression in NCI-H358 was below the level required for reliable analysis. For (B-J), each sample was compared with its corresponding control using Student's t-test (*P < 0.05; **P < 0.01; ***P < 0.001 versus control.).

Fig.S4

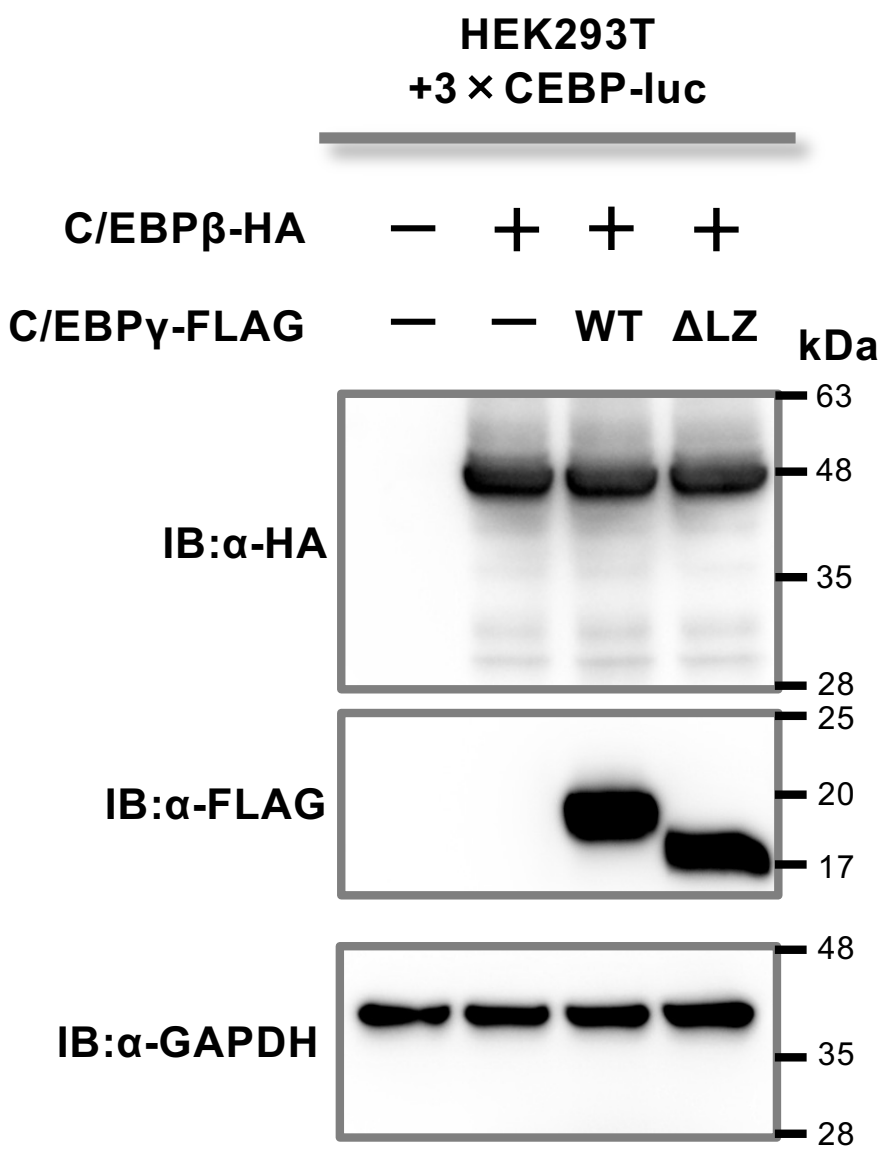

Supplementary Figure 5. Validation of C/EBPγ wild-type, ΔLZ mutant, and C/EBPβ expression in a luciferase reporter assay.

(A) Immunoblot analysis of FLAG- and HA-tagged proteins in HEK293T cells transfected with the 3×C/EBP-luc reporter and the indicated expression plasmids: C/EBPβ-HA alone, C/EBPβ-HA co-transfected with C/EBPγ-FLAG, or C/EBPβ-HA co-transfected with C/EBPγ-ΔLZ-FLAG. GAPDH was used as an internal control.

Fig.S5

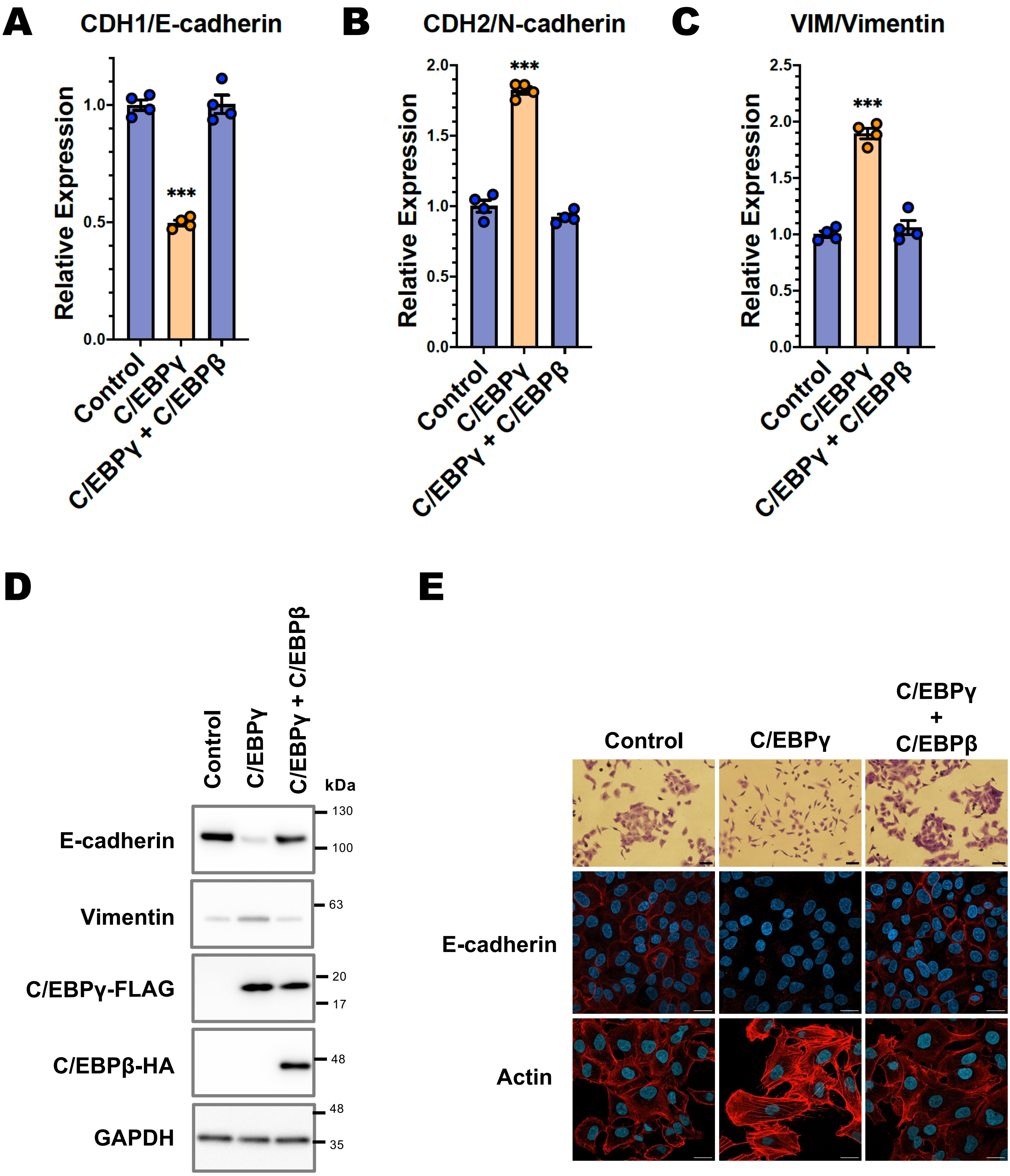
A
CDH1/E-cadherin
Relative Expression
1.0
0.5
0.0
***
Control
C/EBPγ
C/EBPγ + C/EBPβ
B
CDH2/N-cadherin
Relative Expression
2.0
1.5
1.0
0.5
0.0
***
Control
C/EBPγ
C/EBPγ + C/EBPβ
C
VIM/Vimentin
Relative Expression
2.5
2.0
1.5
1.0
0.5
0.0
***
Control
C/EBPγ
C/EBPγ + C/EBPβ
D
Control
C/EBPγ
C/EBPγ + C/EBPβ
kDa
E-cadherin
130
100
Vimentin
63
C/EBPγ-FLAG
20
17
C/EBPβ-HA
48
GAPDH
48
35
E
Control
C/EBPγ
C/EBPγ
+
C/EBPβ
E-cadherin
Actin

Fig.S6

Supplementary Figure 6. Co-expression of C/EBPβ abrogates C/EBPγ-induced EMT phenotypes.

(A–C) qRT-PCR analysis of CDH1 (E-cadherin), CDH2 (N-cadherin), and VIM (Vimentin) expression in A549 cells infected with a control retrovirus, a retrovirus expressing C/EBPγ, or retroviruses co-expressing C/EBPγ and C/EBPβ. DDX5 was used as the internal control. (D) Immunoblot analysis of CDH1/E-cadherin and VIM/Vimentin protein levels in A549 cells expressing FLAG-tagged C/EBPγ alone or co-expressing FLAG-tagged C/EBPγ and HA-tagged C/EBPβ. GAPDH was used as a loading control. (E) Phenotypic characterization of A549 cells expressing C/EBPγ alone or co-expressing C/EBPγ and C/EBPβ. Upper panels, bright-field images showing morphological changes. Scale bars, 200 µm. Middle panels, immunofluorescence images showing E-cadherin localization. Scale bars, 50 µm. Lower panels, actin cytoskeleton visualized by TRITC-phalloidin staining. Scale bars, 50 µm. In (A–C), each sample was compared with its corresponding control using Student's t-test, as previously described (***P < 0.001 versus control.).

Fig.S6

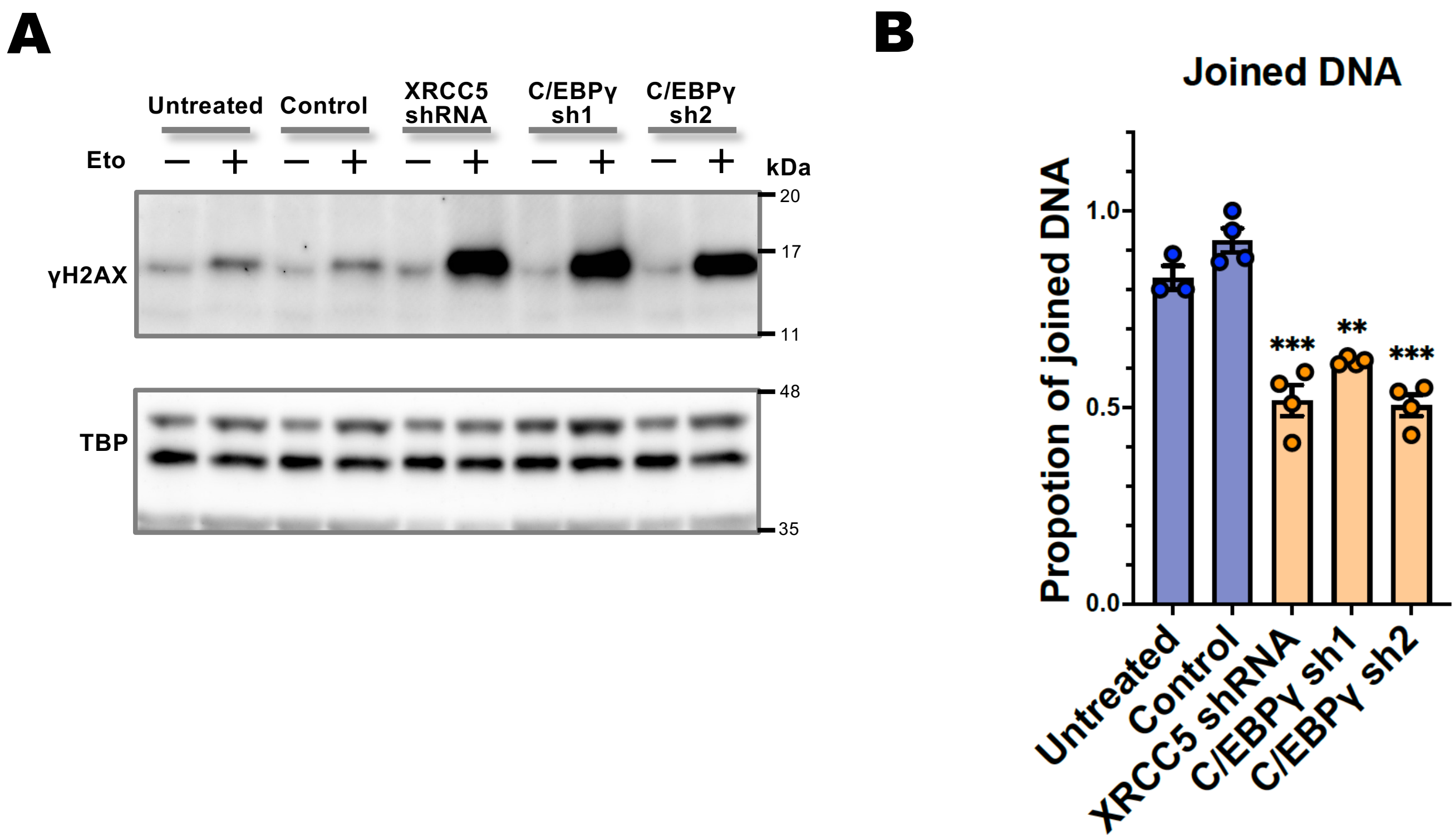

Supplementary Figure 7. Downregulation of C/EBPγ by shRNAs attenuates DSB repair in LUAD cell lines.

(A) A549 cells infected with control lentivirus or lentivirus expressing control shRNA, XRCC5 shRNA, or C/EBPγ shRNAs were treated with 25 μM etoposide. Cells were harvested 48 h after treatment, subjected to SDS–PAGE, and analyzed by immunoblotting with anti–γ-H2AX and anti-TBP antibodies (TBP served as the internal control). (B) qRT-PCR–based end-joining reporter assay in H1299dA3-1 cells infected with lentivirus expressing control shRNA, XRCC5 shRNA, or C/EBPγ shRNAs. The proportion of joined DNA relative to control is shown. GAPDH DNA was quantified as an internal control. Each sample was compared with its corresponding control using Student's t-test (**P < 0.01; ***P < 0.001 versus control.).

Fig.S7

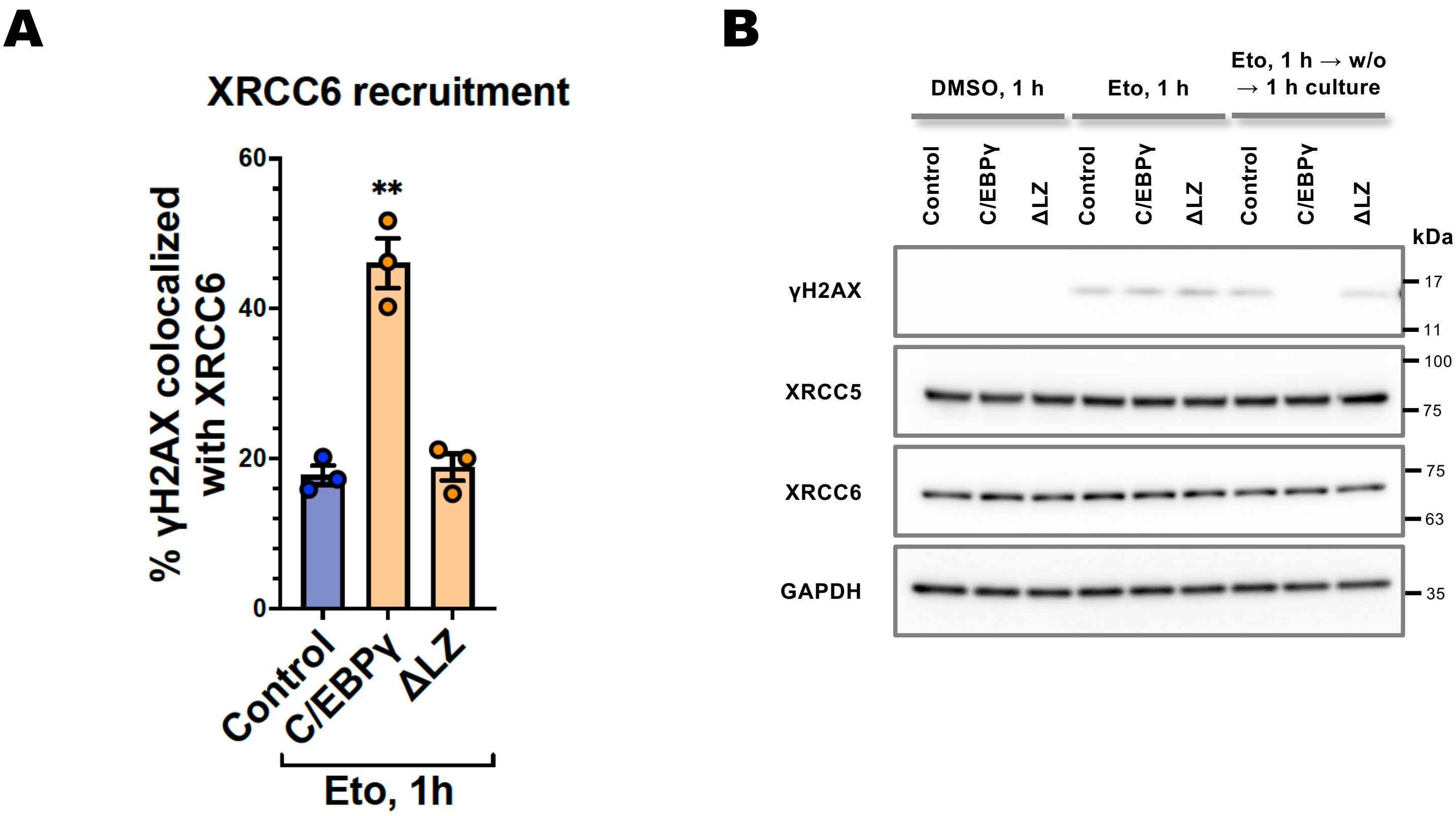

Supplementary Figure 8. C/EBPγ enhances XRCC6 recruitment to DSB sites without altering XRCC5/6 protein levels.

(A) Quantification of XRCC6 recruitment from the immunofluorescence images shown in Fig. 6G. XRCC6 recruitment was assessed as the percentage of γH2AX-positive foci colocalized with XRCC6. At least 40 cells were analyzed per experiment across three independent experiments. (B) Immunoblot analysis of γH2AX, XRCC5 and XRCC6 protein levels in A549 cells expressing FLAG-tagged C/EBPγ or C/EBPγ-ΔLZ. GAPDH was used as a loading control. For (B), each sample was compared with its corresponding control using Student's t-test, as previously described (**P < 0.01 versus control.).

Fig.S8

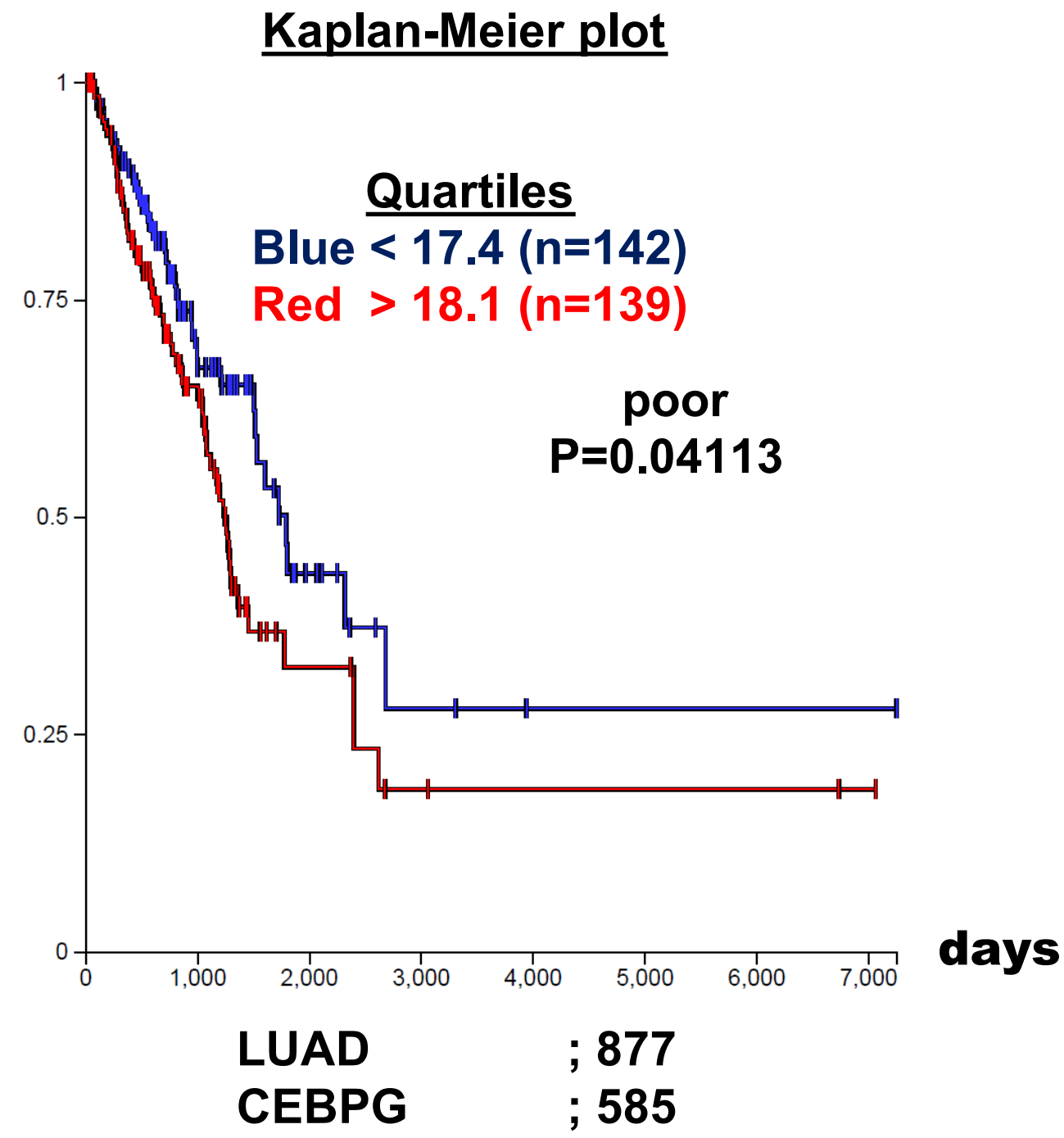

Supplementary Figure 9. C/EBPγ contributes to poor prognosis of LUAD patients.

(A) Kaplan–Meier survival analysis of LUAD patients stratified by C/EBPγ expression.

Fig.S9
