# Supplementary material for: C/EBPγ induces epithelial-mesenchymal transition and facilitates DNA double-strand break repair in lung adenocarcinoma cells
Source: Cell Death Discov. 2026 Jun 2;12:324. doi: 10.1038/s41420-026-03181-0 (PMC13443842; doi:10.1038/s41420-026-03181-0)
Supplement: Supplementary file 2 [file 41420_2026_3181_MOESM2_ESM.pdf]

Supplementary table S1. Top 5% H3K4me3-expanded genes

Gene names and the corresponding lengths (bp) of H3K4me3 domains expanded from the transcription start site toward the gene body in the top 5% of genes following TGF-β–induced EMT, ranked in descending order of domain length.

Gene Name	length (bp)
SERPINE1	6844
FLNA	6602
PDZD8	6228
SMAD7	6167
EFNA1	5304
LAMC2	5258
CTPS1	5062
ANXA5	4788
NDUFB10	4757
BCL2A1	4742
CCDC80	4693
ZFP36	4656
PORCN	4581
KIF3C	4512
LINC-PINT	4481
TGM2	4349
SKIL	4162
SDC4	4119
POU4F2	4020
CMIP	3969
BLOC1S2	3891
COL4A2	3812
PRDX1	3761
FGF2	3734
FRMD8	3725
TSC22D1	3674
PMEPA1	3581
MAP3K8	3548

PLPP6	3531
TRIO	3495
LAMB3	3483
CSRNP1	3434
ETS2	3424
CD44	3372
ANGPTL4	3367
TAGLN	3211
SEMA3C	3162
RPS2	3158
SNORA10	3158
SNORA64	3158
SPATA6L	3058
TUBA4A	3050
COL4A1	3027
SNAI1	2992
C12orf75	2978
MSN	2955
FNDC3B	2934
SH3BP5L	2875
PDGFB	2838
FERMT1	2814
DVL1	2812
DNMBP	2808
ZNF710	2786
CCNL1	2753
HSPA5	2733
COL22A1	2722
RND1	2709
PRPSAP2	2697
VIM	2659
ADAM19	2650
SLC7A5	2649
ELK3	2637
SLC7A1	2629

ASPH	2621
BMPR1B	2589
ITGA3	2570
FLNB	2567
TIPARP	2562
IRAK2	2550
BMP6	2528
MIR193B	2510
MIR193BHG	2510
RBMS2	2473
ITPRIPL2	2458
RFFL	2416
TPD52	2383
PTPRK	2379
TGFBR1	2371
ING2	2368
VGLL3	2359
THBS1	2354
ADGRF4	2352
SLC4A7	2327
SEMA7A	2313
TIAL1	2309
TNFAIP3	2306
ACTR3	2305
RPS18	2301
SMTN	2279
TGFBI	2265
LINC00449	2243
LMCD1	2235
PDXK	2235
F2RL1	2233
NPEPPS	2230
SPRY4	2229
PVR	2226
CAPN2	2197

F3	2181
TRIM8	2181
RDX	2180
PLEK2	2163
FAM83G	2160
LRRC8C	2157
PLAU	2155
ABHD2	2095
MIR100	2092
ZXDC	2072
NKILA	2064
TNS1	2059
EPHA7	2056
P4HA3	2054
FOXB1	2053
NT5E	2042
ARHGAP24	2040
RNF182	2037
PIK3IP1	2025
TNFAIP6	2009
NHS	2001
CALD1	1978
GALNT10	1963
KCTD11	1962
RNU86	1952
RPL3	1952
SNORD43	1952
SNORD83A	1952
PODXL	1934
KIRREL3	1932
GLIPR1	1930
MIR6892	1923
ZYX	1923
C3orf52	1920
MEF2D	1919

CMTM3	1914
ILK	1910
IVNS1ABP	1885
USP31	1883
PID1	1870
SLC20A1	1870
TMEM2	1853
HSPG2	1843
GPAT4	1824
B4GALT1	1812
SLC25A37	1795
MYH9	1788
MYO10	1781
CDC42SE1	1780
PLEC	1775
EEPD1	1775
ADGRE5	1772
TRIM9	1757
SLC7A5P2	1733
CEBPG	1731
ITGA2	1722
KLF6	1718
FAM120C	1705
OGFRL1	1697
MED15	1684
CCND1	1676
STK17B	1674
RPS24	1669
DDB2	1667
MEIOB	1663
AGRN	1661
MIR4657	1653
PURB	1653
SPDL1	1651
CACTIN-AS1	1647

ADAMTSL4-	1643
MCL1	1643
CEP170	1636
SAMD4A	1622
SMS	1615
VPS37C	1589
MIR181A2H(	1589
MRC2	1583
ADAM9	1580
GJA1	1578
GALNT9	1575
RPS15A	1566
DCBLD1	1551
BMP1	1542
FNDC3A	1490
MYOCD	1462
FUT4	1458
ABLIM3	1454
PPP1R9B	1450
KLF10	1446
TBC1D7	1438
TBC1D7-LO(	1438
RGS2	1432
CNPPD1	1423
MORF4L2	1422
PFKP	1421
NDST1	1408
LAMB1	1405
EPC1	1396
TOP1	1384
SOCS2	1376
YWHAG	1373
SSR3	1372
ADAMTS15	1367
GGPS1	1367

RPSA	1366
SNORA6	1366
ANLN	1362
MORC3	1359
TNS4	1357
SIM2	1357
DNAJA1	1353
LPXN	1351
FERMT2	1343
GPRC5A	1343
FZR1	1325
ST3GAL5	1323
TBX3	1323
NLK	1322
CREBZF	1320
STARD4	1316
LINC00862	1314
NAP1L1	1303
DDX3X	1296
JAK1	1281
HMX3	1281
JUNB	1280
FAM43A	1278
FAM200B	1276
PAWR	1275
NRDC	1272
EZR	1270
CHORDC1	1260
PCNX2	1257
PURA	1254
ZNF697	1252
CTGF	1252
ITGA5	1249
TFPI2	1242
GRB10	1240

AAMDC	1231
RHBDF2	1230
PPP2CA	1230
ELL2	1229
PDCD6IP	1226
RPL11	1220
TIMP1	1216
BMP7	1214
SLC2A3	1212
DLST	1209
CASP8	1207
KRT7	1206
BMP2	1205
KANSL3	1202
FN1	1197
PGM2L1	1196
ABTB2	1194
PSD3	1191
IL15	1181
MSANTD3	1179
SLC19A2	1178
CCAT1	1177
FRMD5	1174
HNF1B	1173
HDAC7	1152
SERPINE2	1140
ZNF521	1139
C6orf141	1136
ACTN1	1133
YWHAQ	1130
ODC1	1129
SNORA80B	1129
ULK1	1126
CSRP1	1125
MLXIP	1125

RNF121	1122
IL11	1119
RAB33B	1117
OGT	1115
S1PR5	1114
SRSF8	1106
GPRIN3	1104
SV2C	1103
CDK6	1100
LOC1019271	1097
NUDT4	1095
NUDT4P1	1095
NUDT4P2	1095
PIEZO1	1094
FRMD6-AS1	1093
ATP2A2	1088
HACD3	1087
LBH	1082
BAMBI	1082
RTN4	1080
PXN	1077
PAIP1	1076
MYL6	1076
MICALCL	1073
RFK	1073
EPHB2	1073
IRF2BPL	1072
COL27A1	1071
HNRNPH3	1069
SLC35F2	1068
C7orf49	1066
PYGL	1066
LOC729987	1065
TIMP2	1055
PACS1	1055

COL7A1	1053
RPL24	1052
LRCH1	1051
MTAP	1051
CDK1	1051
IL32	1050
CDH11	1050
SNRPD1	1049
CORO1C	1048
MYL12B	1047
CD59	1046
C15orf48	1045
CRLF1	1044
TM2D2	1042
ZC3H12C	1039
SPACA6P-AS	1038
KLF7	1035
SORCS2	1035
ITGB5	1033
CLTCL1	1031
DZIP1L	1026
FDX1	1026
HNRNPA2B1	1024
SMURF2	1023
FOXD1	1023
TCP11L1	1022
ENO3	1020
SDC1	1019
TRA2B	1017
DDX11	1017
GNG11	1017
FBLN1	1011
SERBP1	1010
LINC01132	1009
IFT57	1006

RAI14	1005
USB1	1003
CITED4	1002
DDI2	1001
OSMR	999
RPS6KA3	989
PTK7	989
AKAP2	986
MASTL	982
PLS3	981
CFLAR	981
PSMD2	980
KDM6B	979
FOXP1	979
AGPS	976
CDCA4	975
DVL2	973
YTHDF1	968
LOC1002881	968
SRSF3	968
EPAS1	967
CSGALNACT	966
SORCS3	965
GPR157	964
SRCAP	964
UBE2R2	964
SPOCK1	963
FAM129B	958
MT1X	957
DOCK4	952
GPC6	952
ENTPD7	951
ATXN2	950
NABP1	949
JUN	946

UXS1	943
SH2B3	943
FANCA	942
HBEGF	942
MIR31HG	942
LRP1	942
AKAP12	936
PPME1	935
HIP1R	934
PHLDA1	933
VEGFA	932
CNN3	931
ASAP2	931
TSPYL1	930
CDC42EP1	930
PPDPF	924
SIAH2	922
TNFRSF1A	922
NPTX1	916
DNAJB2	914
ARF6	910
IPO7	908
MEX3C	906
LRRN2	903
MARS2	903
COX20	903
TFB2M	902
FSD1L	902
SYT13	899
BIRC6	896
RUSC2	896
FHOD3	896
CHST11	893
RAP2B	893
PARN	892

BAZ2B	891
EXT1	890
SETBP1	890
DAPK3	888
WDR59	888
BMF	886
NUAK2	885
YAF2	884
MFSD12	884
SKI	883
UBASH3B	883
LINC01021	881
MAFA	874
RNF168	873
MICAL2	872
PRDM1	872
VCP	870
S100A11	868
CDH4	868
PPRC1	868
VCL	867
HAUS3	865
POLN	865
ZDHHC16	865
SIRPA	864
CCT2	863
MIR3913-2	863
LTBP3	860
MBOAT2	860
CNNM4	860
BZW2	860
NUAK1	859
ZNF143	859
PLCXD2	858
TMEM265	857

VDR	857
CAPRIN2	854
GLS	853
FTSJ1	853
RGMB	852
HSPB8	851
HPS5	850
GABRQ	850
CA8	849
TCEAL1	849
KCNK3	847
MAP7	847
ASPHD2	846
SMIM14	845
PTRF	845
SPOPL	843
TMEM51	840
HNRNPA3	840
LINC01186	839
KLHL36	835
CXCR7	834
UTP18	834
SKA2	829
MAF	829
PRKAB2	828
RPS14	824
GTF2H1	823
CHD8	822
ANO6	820
NARS	819
LIPH	817
C4orf26	816
PADI2	816
TMOD3	815
CPEB4	813

PKNOX2-AS	812
LRP11	811
UACA	811
BPGM	811
GNPNAT1	810
ACOT9	810
LSM14A	810
NGRN	810
SEC61A1	808
KLHL28	806
XYLT1	805
TUFT1	805
ESAM	804
SNHG18	804
GADD45A	804
FBXO5	802
CLTA	802
OSMR-AS1	801
WDR43	800
CCL2	798
PDLIM7	796
LARP6	795
PHTF1	795
ARL6IP6	793
KPNA3	790
YY1AP1	789
OXNAD1	789
RPL30	788
COPB1	787
ACP2	787
FIBCD1	785
FYTTD1	785
LOC100287C	783
TPM3	782
SMAD4	782

PAIP2	782
PPP2R5A	782
BMPR2	782
TMX1	779
SH3GLB1	779
TAPT1	778
SS18	776
DDX17	776
SNHG12	774
SNORA16A	774
SNORA44	774
SNORA61	774
EIF2S3	774
POU6F1	769
HN1L	769
CLIP1-AS1	768
SH2D4A	766
GSTO1	765
CLU	764
FBXO28	764
AZIN1	762
SNORD93	762
MMP2	761
DNMT3B	761
UBAP2	760
EDN1	759
IL1RAP	756
SEPT10	755
LOC344967	755
MITD1	753
PARP6	753
RAD17	750
HSPA4	749
ARFGAP1	749
ZSWIM4	748

DARS2	748
DDX60L	747
SLC25A51	744
HERPUD2	743
C16orf72	743
C18orf25	743
XPO7	742
CTNND1	742
GOPC	741
GARS	740
ITGAV	738
CCDC50	738
ANKLE2	736
YTHDC1	735
CHST7	735
EFNB2	734
TRABD2B	732
MSMO1	732
SNAI2	731
ANKRD50	730
RAET1G	730
NKD1	730
PDE7A	726
INSIG1	725
PHLDB2	723
MXD1	721
ZBTB38	720
JAG1	719
SNF8	718
STK40	718
PELI1	717
CALU	717
EBI3	716
ZNF597	715
AFAP1L2	715

CHAF1A	714
FGD6	713
PHF3	713
ZADH2	711
RPS9	711
TRIM52	708
IGFBP2	708
NMRK1	706
AMFR	704
SNRPF	702
MIR4442	700
TOP2B	700
MAP1LC3B	700
DR1	700
HERPUD1	700
PTEN	700
DMTF1	700
BVES	699
RHPN2	697
MIR1204	697
PVT1	697
BTF3	697
MIR6125	696
USP15	696
SLC6A17	696
ZIC5	694
SLBP	694
HSPA13	692
ITGB8	690
THRAP3	690
ROCK2	689
SMARCA5	689
CDC42EP3	686
IRX6	685
HIST1H2AG	685

RASGRP1	684
SLC16A13	684
ALDH7A1	683
FKBP1A	683
FKBP1A-SD(	683
MIR6869	683
TIMM17A	683
WHAMMP2	683
TRA2A	681
AMOTL2	680
POLG	679
ARHGAP10	679
LAMC1	678
SLC22A4	678
TPM2	676
PRDX3	675
PFKFB3	675
PRELID3A	675
PDS5B	675
PNP	674
EHD1	673
NONO	673
C5AR2	672
KIAA1191	672
OSER1	672
LOC1002894	672
PLXNA4	671
WAPL	670
MARCH7	670
ADRA1B	669
KDM6A	668
ZBED5-AS1	667
SMURF1	664
DOCK10	664
WDR44	664

TMEM158	663
BZW1	663
MED28	663
PPARG	662
YPEL1	661
HMX1	660
OCIAD2	660
NPC1	660
HNRNPH1	660
NIPAL4	660
TERF1	659
FAM208A	658
OSBPL11	658
AKAP10	657
GSK3A	657
PCGF1	657
BIRC2	657
HCG17	656
HCG18	656
SCD5	655
DENND5A	655
CTTNBP2NL	655
CHMP1B	654
LOC1053698	654
POU4F2	653
PARD3	653
DUSP7	652
EGFR	652
PIAS2	649
SCYL2	649
LOC1002681	647
RNF213	645
MKLN1-AS	644
SLIT3	644
RPS4X	643

SYDE2	643
CTIF	642
PPP1R14C	641
MCM3	637
WNT7A	637
MPDU1	637
VASN	637
EXOC8	636
UNC50	636
BRI3	636
ACTR1B	634
SP100	634
SNHG5	633
SNORD50A	633
SNORD50B	633
NFKB1	633
DHX9	633
HNF4A	633
JPH3	631
PDZRN4	630
ITPR3	630
NFKB2	629
FBXL16	628
SOWAHC	628
PLK3	627
TNFAIP8	626
SKP1	625
IDS	625
PACSIN3	623
LPP	623
HOOK3	623
ARAP2	621
UFM1	620
GRK5	619
LOC440700	619

HNRNPU	618
ZC3HAV1	617
TPM1	617
MBP	616
SARS	616
ZIC2	615
PROCR	615
HMGA2	615
GOSR2	615
GTF2B	614
ARF4	613
PLEKHA3	613
FAM98A	612
DRG2	612
FABP5	610
RASGRF2	610
SCAF11	609
SRP19	609
PPP4R1L	607
GABARAPL1	607
MIR4488	606
ZSCAN29	606
