# Supplementary material for: C/EBPγ induces epithelial-mesenchymal transition and facilitates DNA double-strand break repair in lung adenocarcinoma cells
Source: Cell Death Discov. 2026 Jun 2;12:324. doi: 10.1038/s41420-026-03181-0 (PMC13443842; doi:10.1038/s41420-026-03181-0)
Supplement: Supplementary file 3 [file 41420_2026_3181_MOESM3_ESM.pdf]

Supplementary Table S2. The top 5% upregulated and the top 5% H3K4me3-expanded transcription factor genes

List of transcription factor genes ranked within the top 5% based on either transcriptional upregulation or H3K4me3 domain expansion upon TGF-β–induced EMT. “Fold” indicates the fold change in gene expression measured by RNA-seq after TGF-β treatment. “Length (bp)” represents the genomic length of the expanded H3K4me3 domain extending from the transcription start site toward the gene body. “EMT paper” indicates whether the gene has been previously reported as an EMT-related transcription factor in the literature (〇, reported; ×, not reported), based on published EMT studies.

Gene	Fold	EMT paper
SNAI2	27.9329665	〇
HOXD10	13.5231028	〇
SNAI1	10.1344096	〇
HEY1	9.97605947	〇
PRRX2	9.38099627	〇
FOXP1	8.74594203	〇
JUNB	7.80675332	〇
NKX3-1	7.29889698	〇
MAF	7.18276282	×
JUN	6.5837529	〇
EOMES	6.57680958	〇
HIVEP3	6.12206043	×
NTN1	6.08064577	〇
FOXD1	6.03516222	〇
MXD1	5.97555886	×
ETS2	5.86542955	〇
FOXC2	5.67526939	〇
CREB5	5.55529586	〇
NKX6-1	5.18755093	〇
GLI2	4.83975642	〇
FOSL1	4.69118114	〇
RUNX1	4.4726	〇
ZNF697	4.43118066	×
ELK3	4.24985084	〇

Gene	Length (bp)	EMT paper
SKIL	4162	〇
ETS2	3424	〇
SNAI1	2992	〇
ZNF710	2786	×
ELK3	2637	〇
VGLL3	2359	〇
ZXDC	2072	×
MEF2D	1919	〇
CEBPG	1731	×
KLF6	1718	〇
PURB	1653	×
MYOCD	1462	〇
KLF10	1446	〇
MORF4L2	1422	×
SIM2	1357	〇
TBX3	1323	〇
CREBZF	1320	×
HMX3	1281	×
JUNB	1280	〇
PURA	1254	〇
ZNF697	1252	×
HNF1B	1173	〇
ZNF521	1139	×
CSRP1	1125	×

MAFA	4.212114	×
TFAP2E	4.18044397	×
TSHZ3	4.14210657	×
FOSB	4.08543388	○
SIX3	3.99042379	○
KLF7	3.97845594	○
HNF1B	3.57250062	○
SIM2	3.56511216	○
PLAG1	3.45836728	×
ZEB2	3.42511375	○
CSRNP1	3.41560283	×
ETS1	3.29789611	○
LHX8	3.29512268	×
MLXIP	3.26046822	○
SOX8	3.1590855	○
VDR	3.15554789	○
MSC	3.12294036	○
TCF7	3.10234728	○
SOX18	3.05932491	○
ZNF813	3.04032289	×
SKIL	3.0386361	○
TSHZ1	2.9701272	○
PBX3	2.95871166	○
PAX6	2.94031227	○
OSR1	2.85903927	○
DLX1	2.84003135	○
HLX	2.80408158	×
PRDM1	2.75201641	○
BHLHE41	2.74895861	○
HIVEP2	2.72737666	×
RELB	2.6965756	○
MSX1	2.66028253	○
VAX1	2.66028253	×
VGLL3	6.48443866	○
MYOCD	17.2253294	○
CSRP1	4.28125173	×
LBH	11.4875836	○

MLXIP	1125	×
LBH	1082	○
KLF7	1035	○
FOXD1	1023	○
CITED4	1002	×
FOXP1	979	○
EPAS1	967	○
SRCAP	964	×
JUN	946	○
TFB2M	902	×
SKI	883	○
MAFA	874	×
PRDM1	872	○
ZNF143	859	○
PTRF	845	○
MAF	829	×
GTF2H1	823	×
SMAD4	782	○
SS18	776	×
POU6F1	769	×
SNAI2	731	○
MXD1	721	×
ZBTB38	720	×
PHF3	713	×
DR1	700	×
DMTF1	700	×
BTF3	697	○
ZIC5	694	○
THRAP3	690	×
IRX6	685	×
NONO	673	○
PPARG	662	○
HMX1	660	×
POU4F2	653	○
NFKB1	633	○
HNF4A	633	○
NFKB2	629	×

CITED4	6.14949367	×
ARNTL2	4.542	○

LPP	623	○
ZIC2	615	○
HMGA2	615	○
GTF2B	614	×
ZSCAN29	606	×
