# Supplementary material for: C/EBPγ induces epithelial-mesenchymal transition and facilitates DNA double-strand break repair in lung adenocarcinoma cells
Source: Cell Death Discov. 2026 Jun 2;12:324. doi: 10.1038/s41420-026-03181-0 (PMC13443842; doi:10.1038/s41420-026-03181-0)
Supplement: Supplementary file 4 [file 41420_2026_3181_MOESM4_ESM.pdf]

Supplementary Table S3. Filtered list of proteins co-immunoprecipitated with C/EBPγ

Filtered dataset of proteins co-immunoprecipitated with FLAG-tagged C/EBPγ in A549 cells and identified by mass spectrometry. This table presents a broader set of detected proteins, including both candidate interaction partners and known nonspecific binders, and serves as the source dataset from which the proteins shown in Table 1 were subsequently selected using more stringent criteria. Proteins were filtered using criteria that differed from those applied in Table 1 (number of unique detected peptides ≥ 2 and C/EBPγ–FLAG/Control protein quantity ratio ≥ 5). Protein identification confidence was evaluated based on the combined q-value and classified as high (<0.01), medium (0.01–0.05), or low (>0.05). The table includes protein accession numbers, descriptions, peptide-based identification metrics (coverage, total and unique peptide counts), molecular properties, and quantitative abundance information. Columns highlighting the number of unique detected peptides and the C/EBPγ–FLAG/Control protein quantity ratio indicate the parameters used for protein inclusion. Proteins annotated with asterisks (*) represent common keratin contaminants and were excluded from candidate C/EBPγ interaction partners, while proteins marked with double asterisks (**) indicate those with an abundance ratio ≤ 20 that were excluded from Table 1 but are included here for transparency.

Protein Identification Confidence Based on q-value (Column D) high<0.01, 0.01<medium<0.05, 0.05<low			q-value for Protein Identification	Cumulative Score of Each Peptide	Coverage Rate	Number of Detected Peptides	Total Number of Detected Peptides (Including Redundant and Distinct Charge States)	Number of Unique Detected Peptides								Protein Quantity Ratio		Protein Abundance (F1) – Normalized	Protein Abundance (F2) – Normalized	Raw Protein Abundance Data (F1)	Raw Protein Abundance Data (F2)	Protein Detection (F1)	Protein Detection (F2)
Protein FDR Confidence: Combined	Accession	Description	Exp. q-value: Combined	Sum PEP Score	Coverage [%]	# Peptides	# PSMs	# Unique Peptides	# Protein Groups	# AAs	MW [kDa]	calc. pI	Score Sequest HT: Sequest HT	# Peptides (by Search Engine): Sequest HT	# Razor Peptides	Abundance Ratio: (Sample) / (Control)	Abundance Ratio Variability [%]: (Sample) / (Control)	Abundances (Grouped): Control	Abundances (Grouped): Sample	Abundance: F1: Control	Abundance: F2: Sample	Found in Sample: [S1] F1: Control	Found in Sample: [S2] F2: Sample
High	P02538	*Keratin, type II cytoskeletal 6A [OS=Homo sapiens]	0	45.466	27	17	37	4	1	564	60	8	84.04	17	0	100			200		1826749.625	Not Found	High
High	P13010	X-ray repair cross-complementing protein 5 [OS=Homo sapiens]	0	26.328	19	10	10	10	1	732	82.7	5.81	24.17	10	0	100			200		4497288.453	Not Founc	High
High	P53567	CCAAT/enhancer-binding protein gamma [OS=Homo sapiens]	0	42.409	59	10	39	10	1	150	16.4	9.77	101.01	10	0	100	157.55	1.2	198.8	36203324.73	3999528147	Peak Foun	High
High	O75531	Barrier-to-autointegration factor [OS=Homo sapiens]	0	10.124	51	3	3	3	1	89	10.1	6.09	11.91	3	0	100			200		6353837	Not Founc	High
High	P17676	CCAAT/enhancer-binding protein beta [OS=Homo sapiens]	0	6.591	13	3	3	3	1	345	36.1	8.31	6.19	3	0	100			200		2076531.258	Not Founc	High
High	Q7Z6I8-1	UPF0461 protein C5orf24 [OS=Homo sapiens]	0	3.861	16	2	2	2	1	188	20.1	9.85	5.91	2	0	100			200		316527.4375	Not Founc	High
High	P09874	Poly [ADP-ribose] polymerase 1 [OS=Homo sapiens]	0	9.474	3	2	2	2	1	1014	113	8.88	8.21	2	0	100			200		296652.5313	Not Founc	High
High	P12956	X-ray repair cross-complementing protein 6 [OS=Homo sapiens	0	15.577	12	7	7	7	1	609	69.8	6.64	13.57	7	0	21.812	0	0.8	199.2	26017.72656	4380285.625	Peak Foun	High
High	P0C0S8	**histone H2A type 1 [OS=Homo sapiens]	0	32.622	35	6	31	2	1	130	14.1	10.9	98.22	6	0	9.701	64.42	25.7	174.3	14447622.16	66510433.92	High	High
High	P08779	*Keratin, type I cytoskeletal 16 [OS=Homo sapiens]	0	62.896	46	20	36	8	1	473	51.2	5.05	88.5	20	0	9.604	60.56	9.7	190.3	353034.2813	4702200.688	Peak Foun	High
High	P0C0S5	**Histone H2A.Z [OS=Homo sapiens]	0	15.394	31	5	15	3	1	128	13.5	10.58	36.55	5	0	9.431	0	8.7	191.3	897279.375	13290554.5	Peak Foun	High
High	P62805	**histone H4 [OS=Homo sapiens]	0	58.771	59	13	45	13	1	103	11.4	11.36	136.81	13	0	7.555	15	20.2	179.8	112009192	674149678.2	High	High
High	Q8IUE6	**Histone H2A type 2-B [OS=Homo sapiens]	0	14.553	35	4	8	3	1	130	14	10.89	26.45	4	0	5.672	0	21.8	178.2	2030542.5	11243133.88	Peak Foun	High
High	Q5QNW6-	**Isoform 2 of Histone H2B type 2-F [OS=Homo sapiens]	0	24.794	31	4	22	4	1	134	14.8	10.35	72.99	4	0	5.426	20.95	32.2	167.8	193051035.3	681374145.6	High	High
High	P04908	**histone H2A type 1-B/E [OS=Homo sapiens]	0	30.939	35	6	22	2	1	130	14.1	11.05	71.18	6	4	5.391	15.1	33.6	166.4	199394700.7	668234275.8	High	High

*Because these keratins are some of the most common contaminant peptides (Journal of Proteomics, Volume 88, 2 August 2013, Pages 92-103), they were excluded from the candidate interaction partners of CEBPG.

**Peptides with an Abundance Ratio (WT/Control) ≤ 20 in the Protein Quantity Ratio analysis were excluded from Table 1.
