# Supplementary material for: C/EBPγ induces epithelial-mesenchymal transition and facilitates DNA double-strand break repair in lung adenocarcinoma cells
Source: Cell Death Discov. 2026 Jun 2;12:324. doi: 10.1038/s41420-026-03181-0 (PMC13443842; doi:10.1038/s41420-026-03181-0)
Supplement: Supplementary file 5 [file 41420_2026_3181_MOESM6_ESM.pdf]

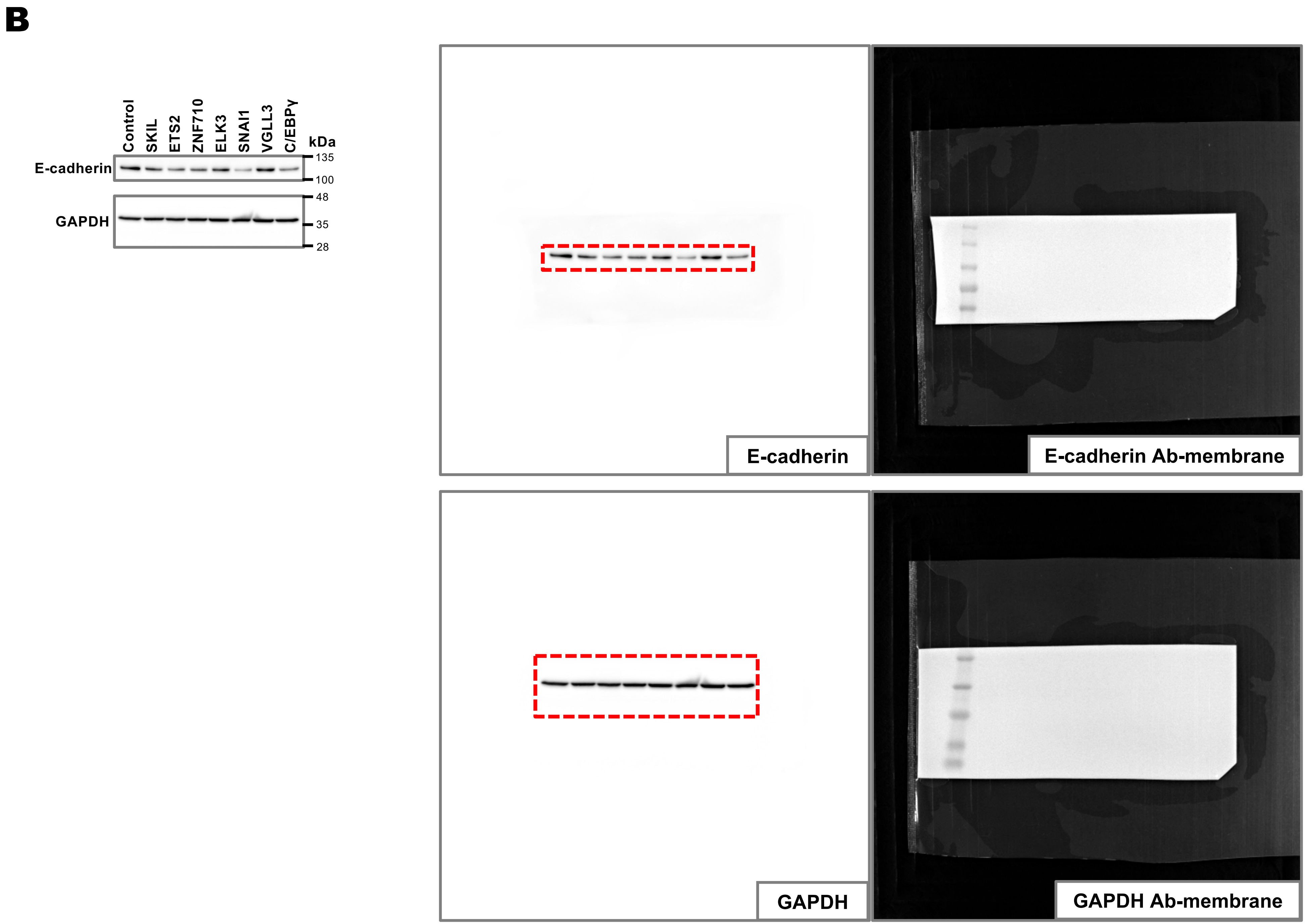
B
Control
SKIL
ETS2
ZNF710
ELK3
SNAI1
VGLL3
C/EBPγ
kDa
E-cadherin
135
100
48
GAPDH
35
28
E-cadherin
E-cadherin Ab-membrane
GAPDH
GAPDH Ab-membrane

Fig.1

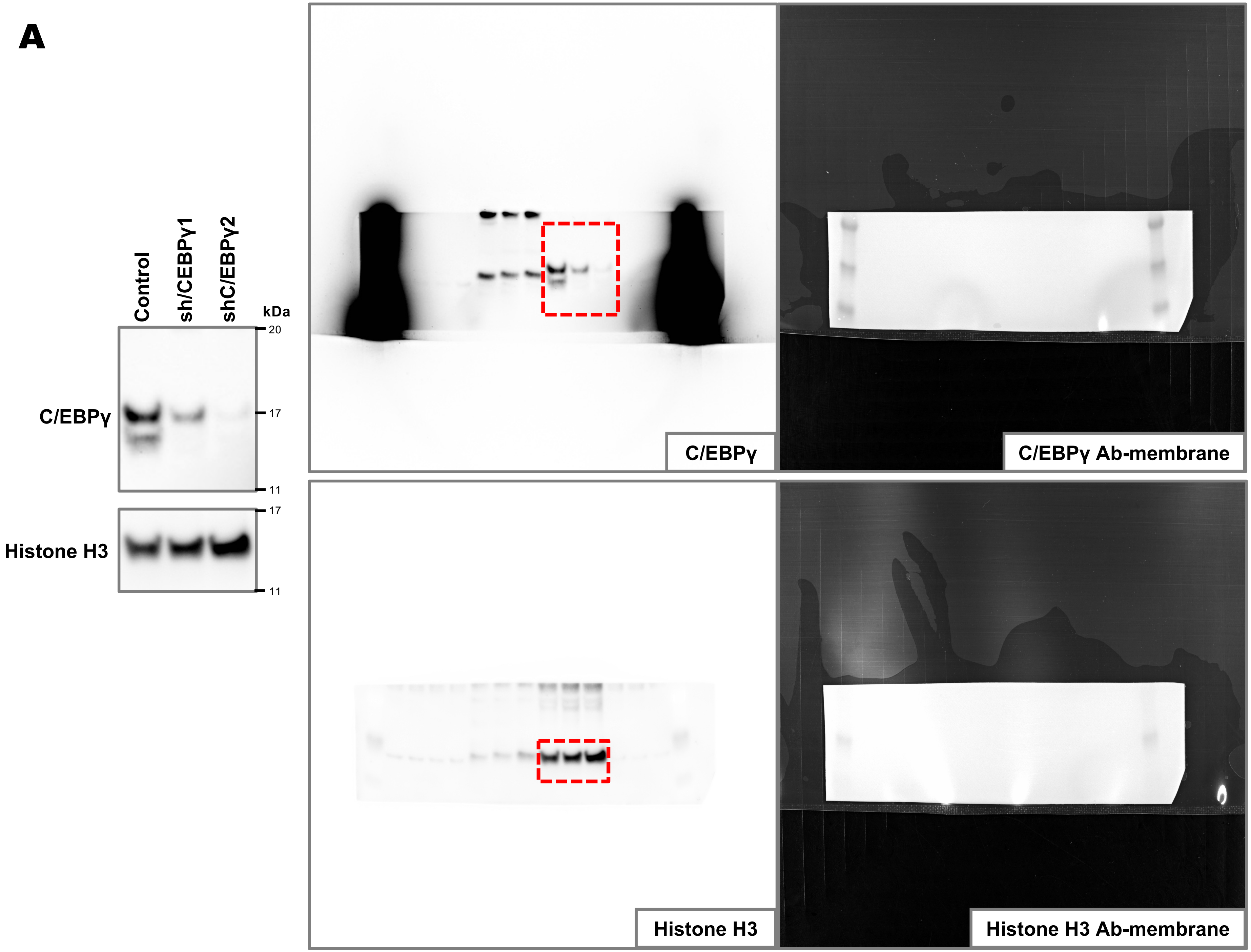
A
Control
sh/CEBPγ1
shC/EBPγ2
kDa
20
17
11
17
11
C/EBPγ
Histone H3
C/EBPγ
C/EBPγ Ab-membrane
Histone H3
Histone H3 Ab-membrane

Fig.2

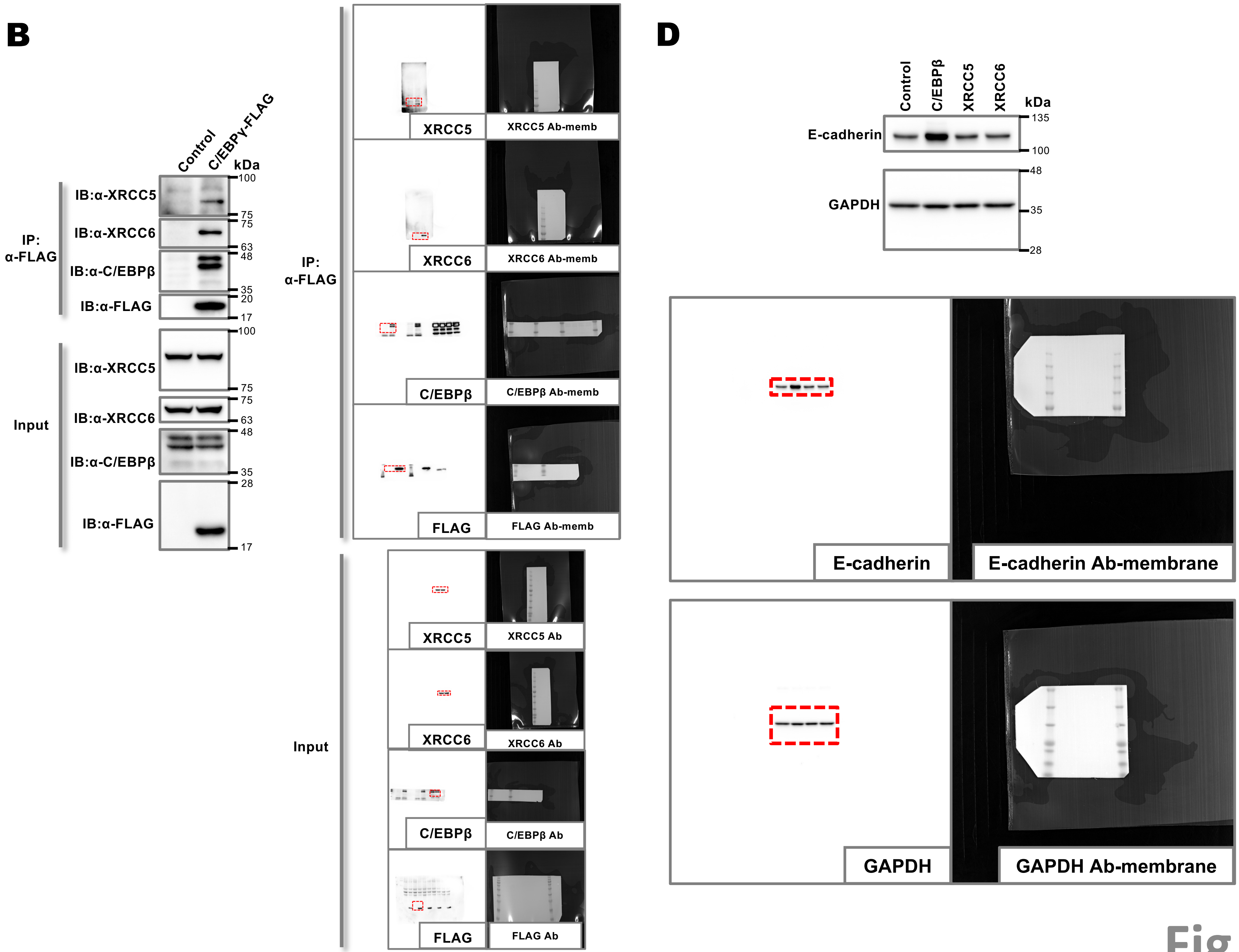

B
Control
C/EBPγ-FLAG
kDa
IP: α-FLAG
IB:α-XRCC5
IB:α-XRCC6
IB:α-C/EBPβ
IB:α-FLAG
Input
IB:α-XRCC5
IB:α-XRCC6
IB:α-C/EBPβ
IB:α-FLAG
100
75
75
63
48
35
20
17
100
75
75
63
48
35
28
17
IP: α-FLAG
XRCC5
XRCC5 Ab-memb
XRCC6
XRCC6 Ab-memb
C/EBPβ
C/EBPβ Ab-memb
FLAG
FLAG Ab-memb
Input
XRCC5
XRCC5 Ab
XRCC6
XRCC6 Ab
C/EBPβ
C/EBPβ Ab
FLAG
FLAG Ab
D
Control
C/EBPβ
XRCC5
XRCC6
kDa
E-cadherin
135
100
GAPDH
48
35
28
E-cadherin
E-cadherin Ab-membrane
GAPDH
GAPDH Ab-membrane

Fig.4

F

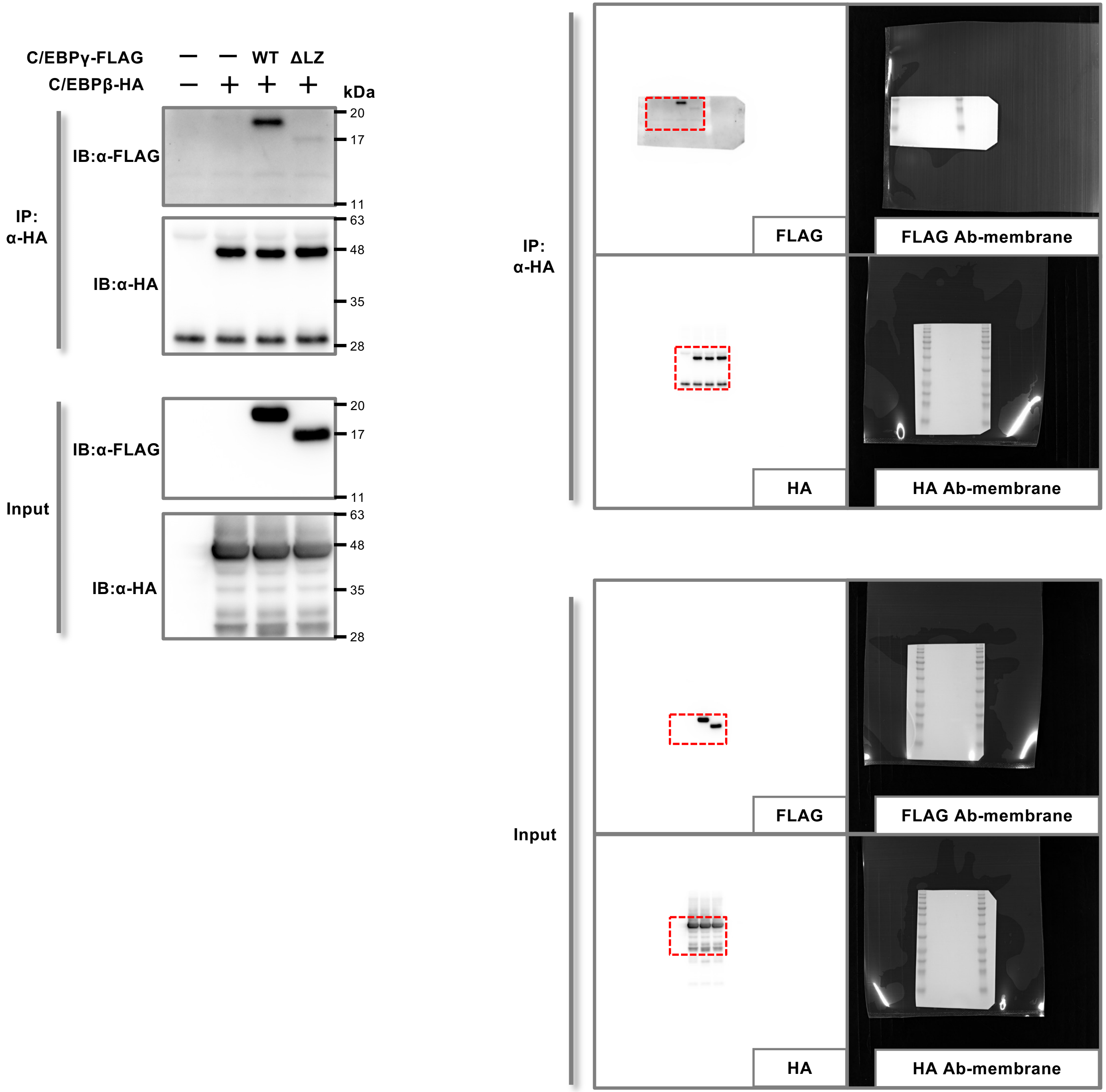
C/EBPγ-FLAG
C/EBPβ-HA
WT
ΔLZ
kDa
IB:α-FLAG
IB:α-HA
IP:
α-HA
Input
20
17
11
63
48
35
28
FLAG
FLAG Ab-membrane
HA
HA Ab-membrane

Fig.5

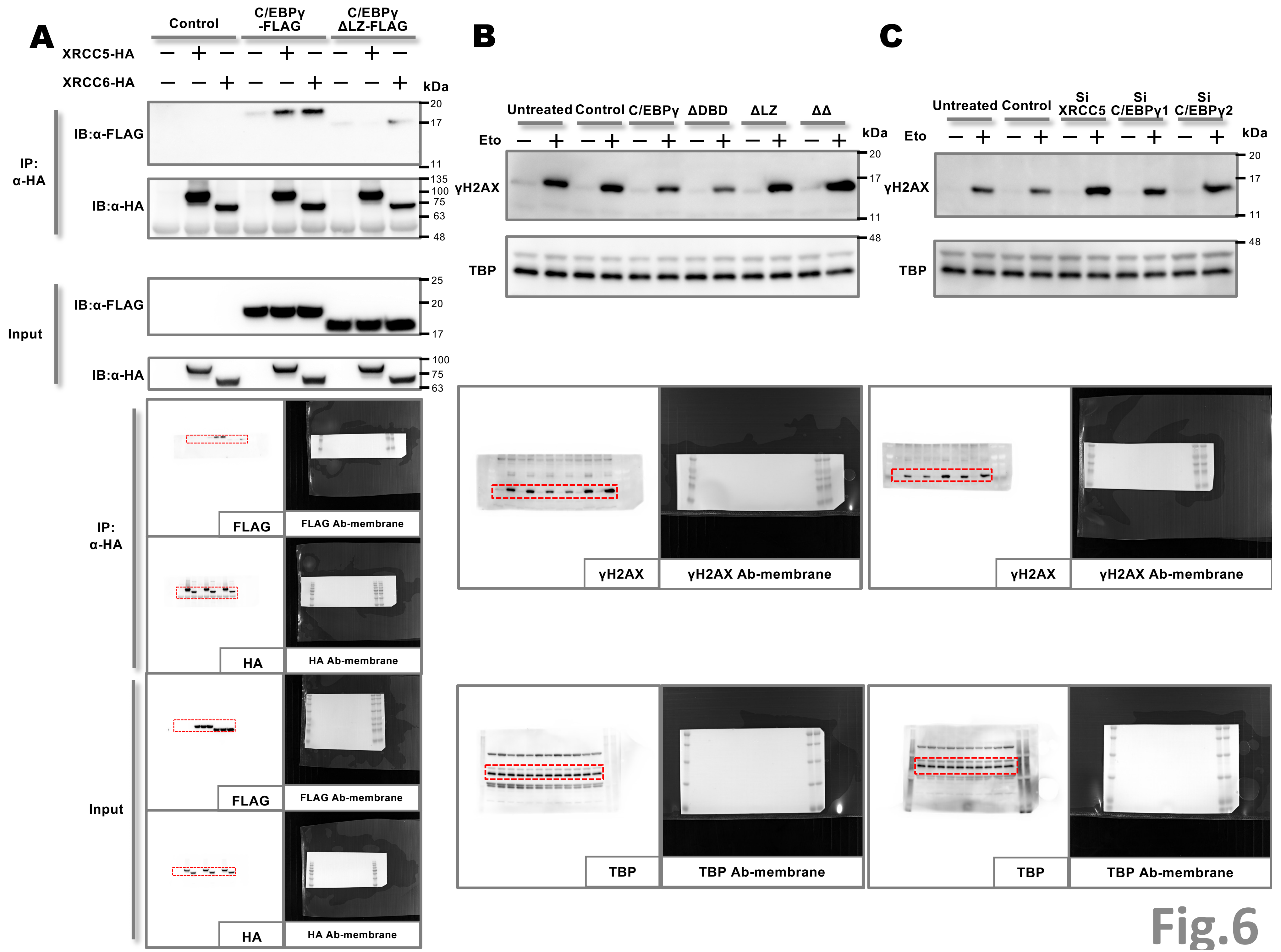
A
Control
C/EBPγ -FLAG
C/EBPγ ΔLZ-FLAG
XRCC5-HA
XRCC6-HA
kDa
IP: α-HA
IB:α-FLAG
IB:α-HA
Input
IB:α-FLAG
IB:α-HA
20
17
11
135
100
75
63
48
25
FLAG
FLAG Ab-membrane
HA
HA Ab-membrane
B
Untreated
Control
C/EBPγ
ΔDBD
ΔLZ
ΔΔ
Eto
γH2AX
TBP
γH2AX Ab-membrane
TBP Ab-membrane
C
Si XRCC5
Si C/EBPγ1
Si C/EBPγ2
Fig.6

G

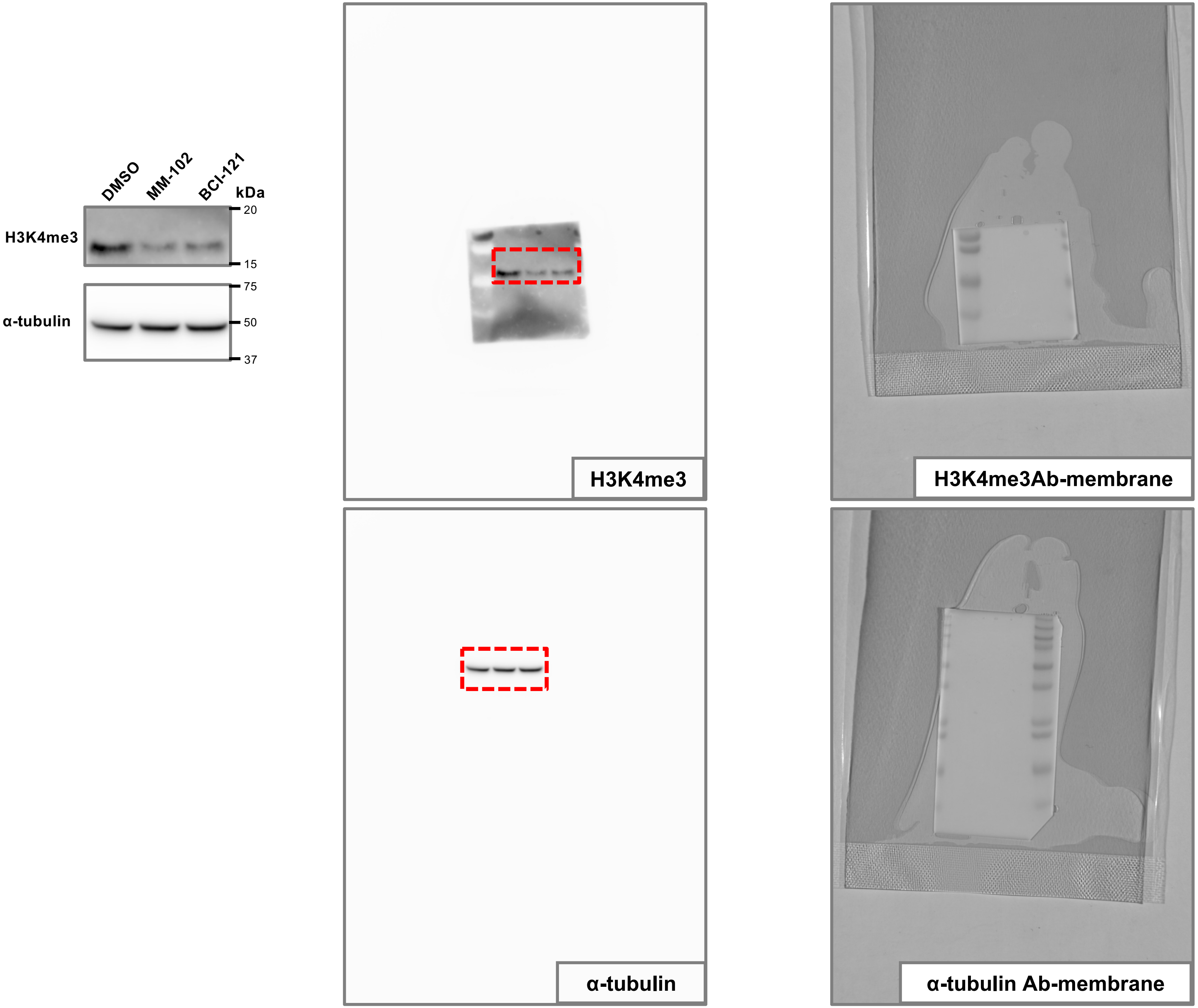
DMSO
MM-102
BCI-121
kDa
H3K4me3
α-tubulin
20
15
75
50
37
H3K4me3
H3K4me3Ab-membrane
α-tubulin
α-tubulin Ab-membrane

Fig.S1

F

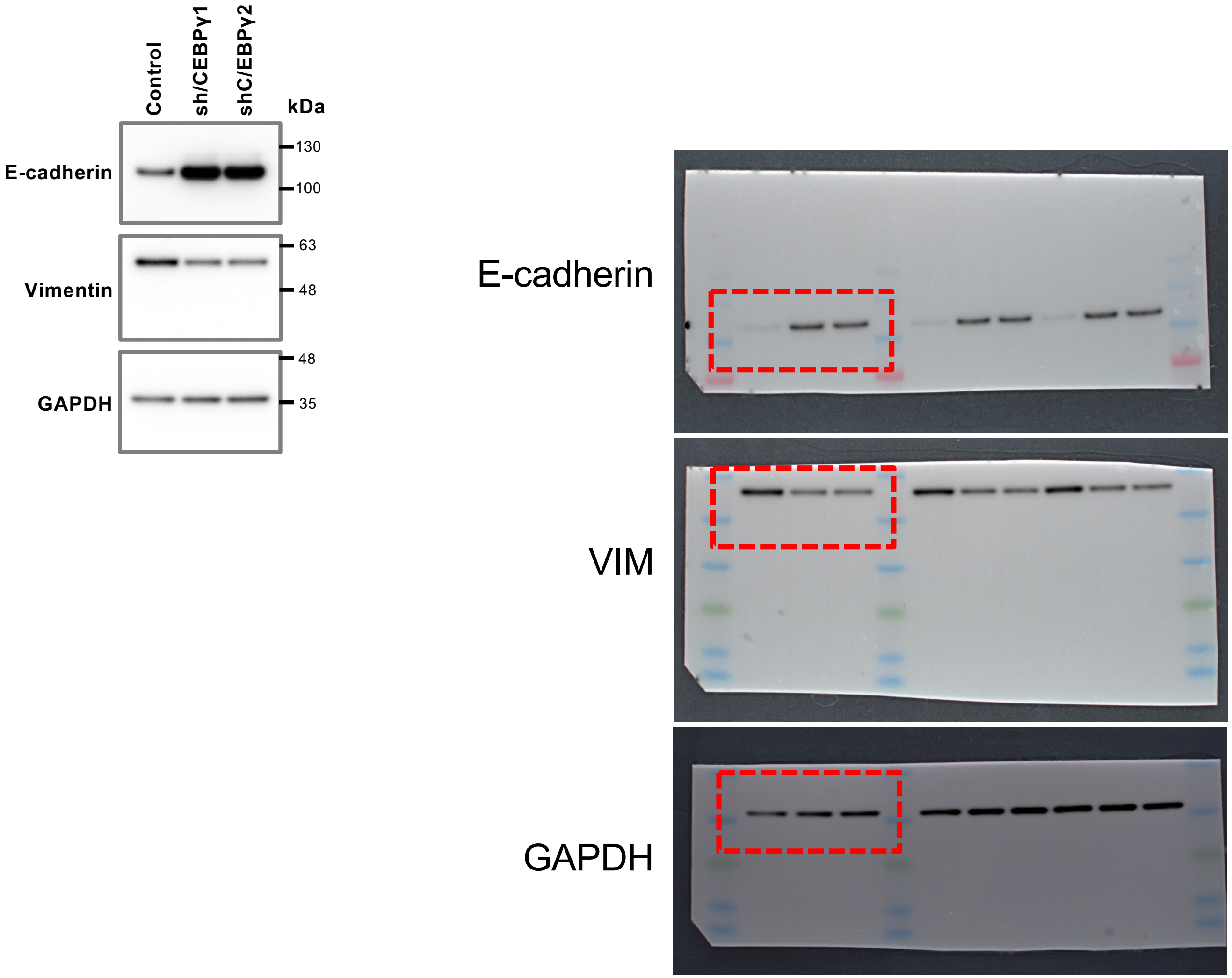
Control
sh/CEBPγ1
shC/EBPγ2
kDa
E-cadherin
130
100
63
Vimentin
48
48
GAPDH
35
E-cadherin
VIM
GAPDH

Fig.S2

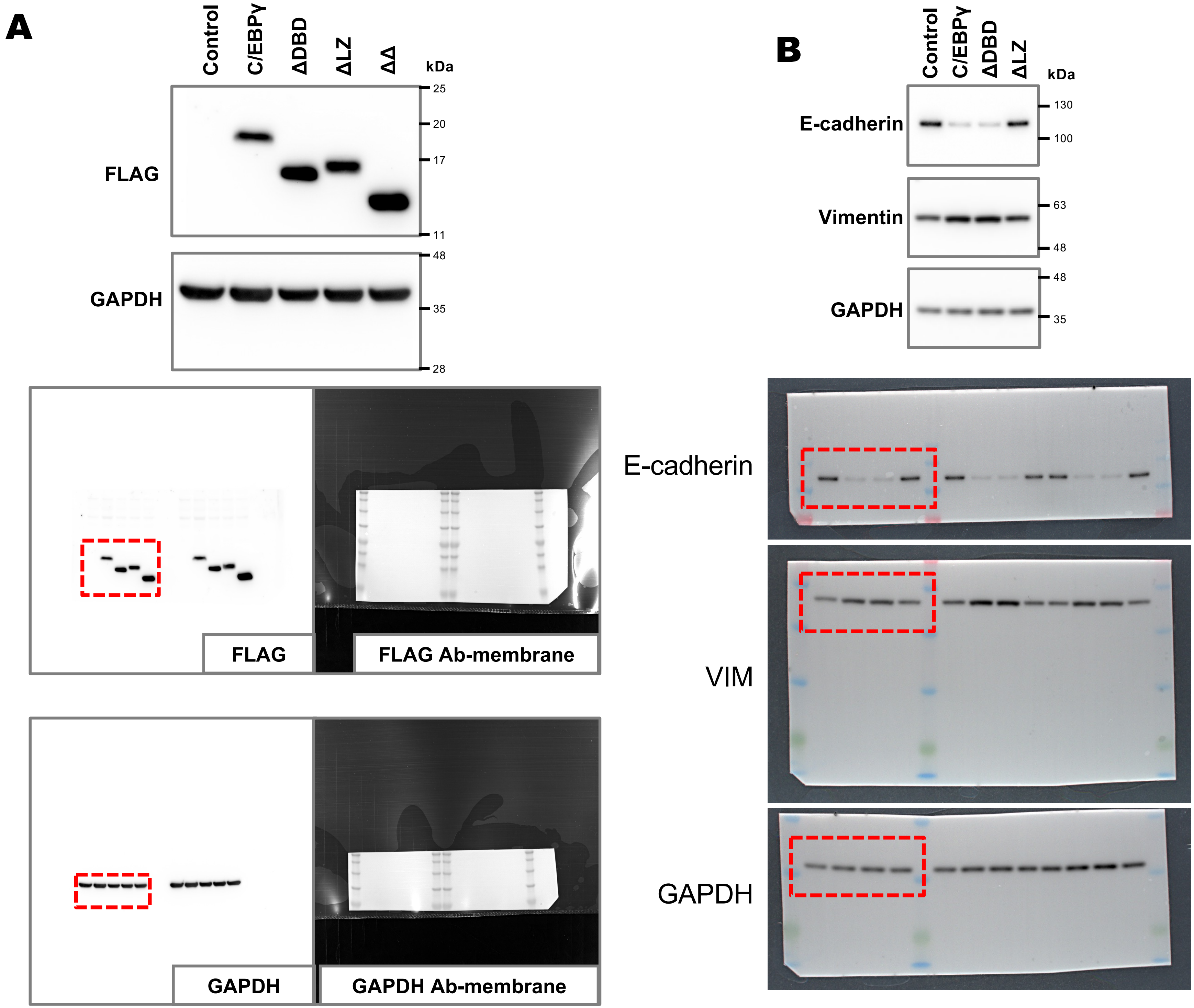
A
Control
C/EBPγ
ΔDBD
ΔLZ
ΔΔ
kDa
25
20
17
11
FLAG
48
35
28
GAPDH
FLAG
FLAG Ab-membrane
GAPDH
GAPDH Ab-membrane
B
Control
C/EBPγ
ΔDBD
ΔLZ
kDa
E-cadherin
130
100
Vimentin
63
48
GAPDH
48
35
E-cadherin
VIM
GAPDH

Fig.S3

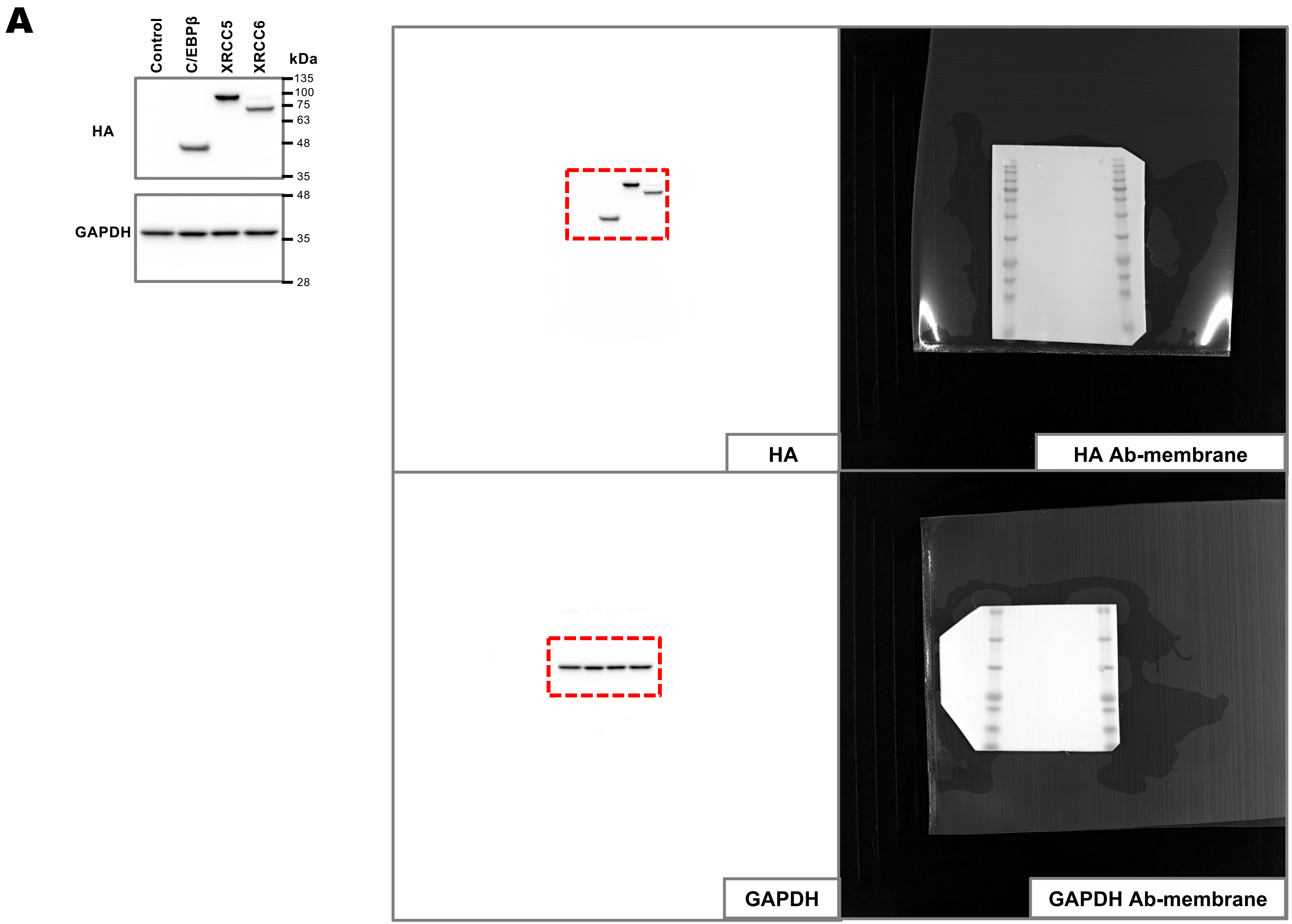

＊The same loading GAPDH blot as in Figure 4D was used.

Fig.S4

A

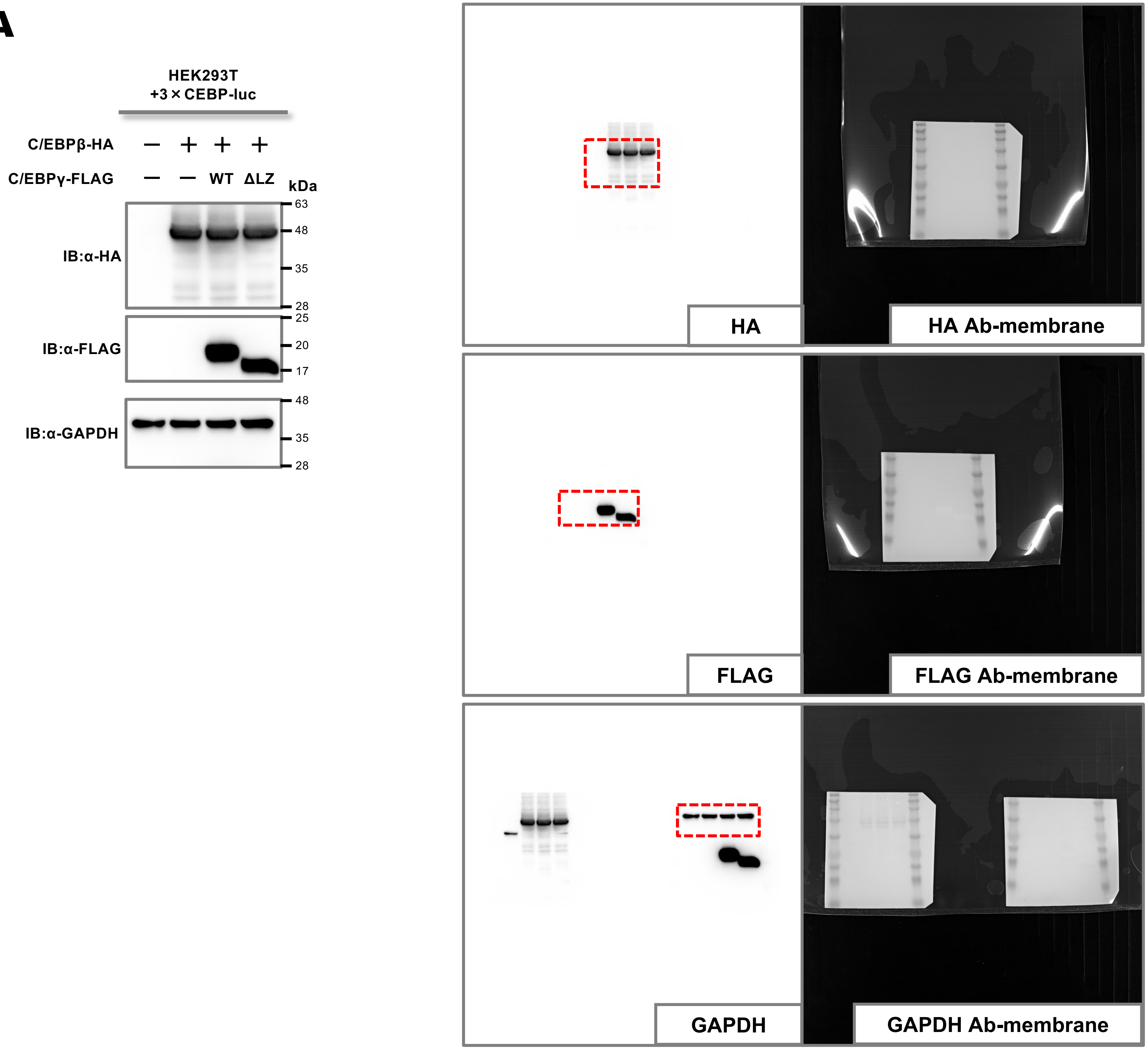
HEK293T
+3×CEBP-luc
C/EBPβ-HA — + + +
C/EBPγ-FLAG — — WT ΔLZ
kDa
IB:α-HA
63
48
35
28
25
IB:α-FLAG
20
17
IB:α-GAPDH
48
35
28
HA
HA Ab-membrane
FLAG
FLAG Ab-membrane
GAPDH
GAPDH Ab-membrane

Fig.S5

D

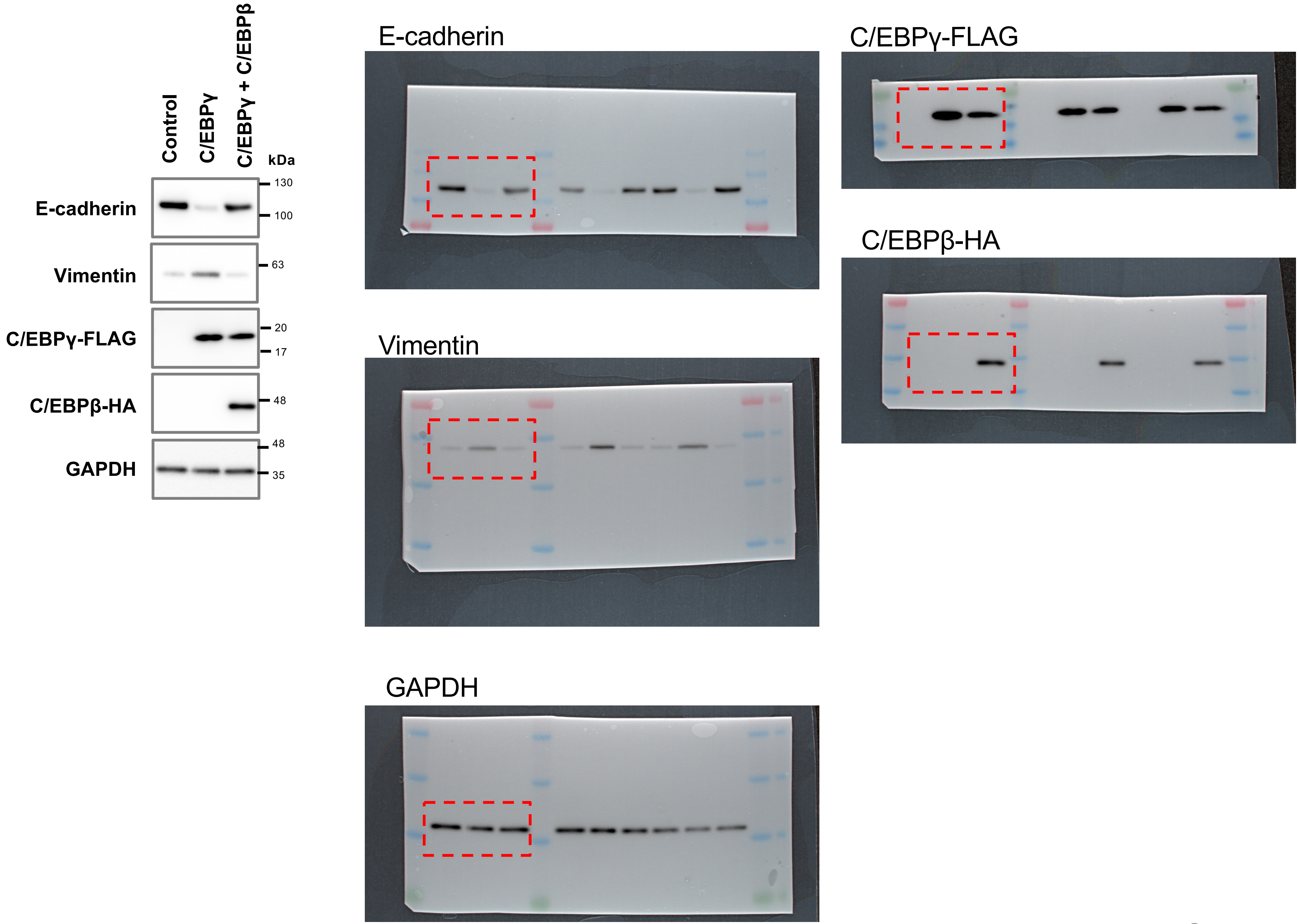
Control
C/EBPγ
C/EBPγ + C/EBPβ
kDa
E-cadherin
130
100
Vimentin
63
C/EBPγ-FLAG
20
17
C/EBPβ-HA
48
GAPDH
48
35
E-cadherin
Vimentin
GAPDH
C/EBPγ-FLAG
C/EBPβ-HA

Fig.S6

A

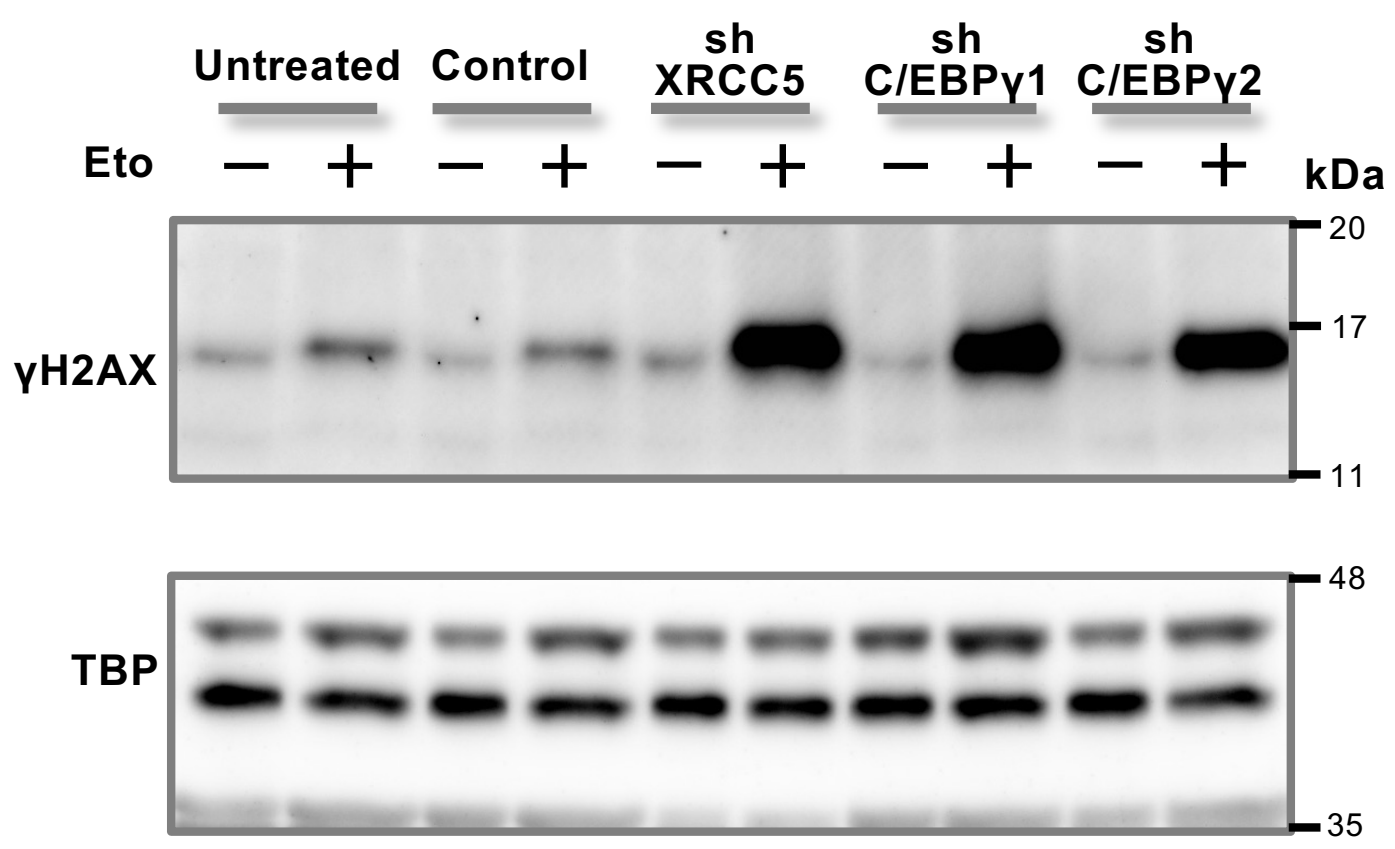

Untreated
Control
sh XRCC5
sh C/EBPγ1
sh C/EBPγ2
Eto
− + − + − + − + − +
kDa
20
17
11
48
35
γH2AX
TBP

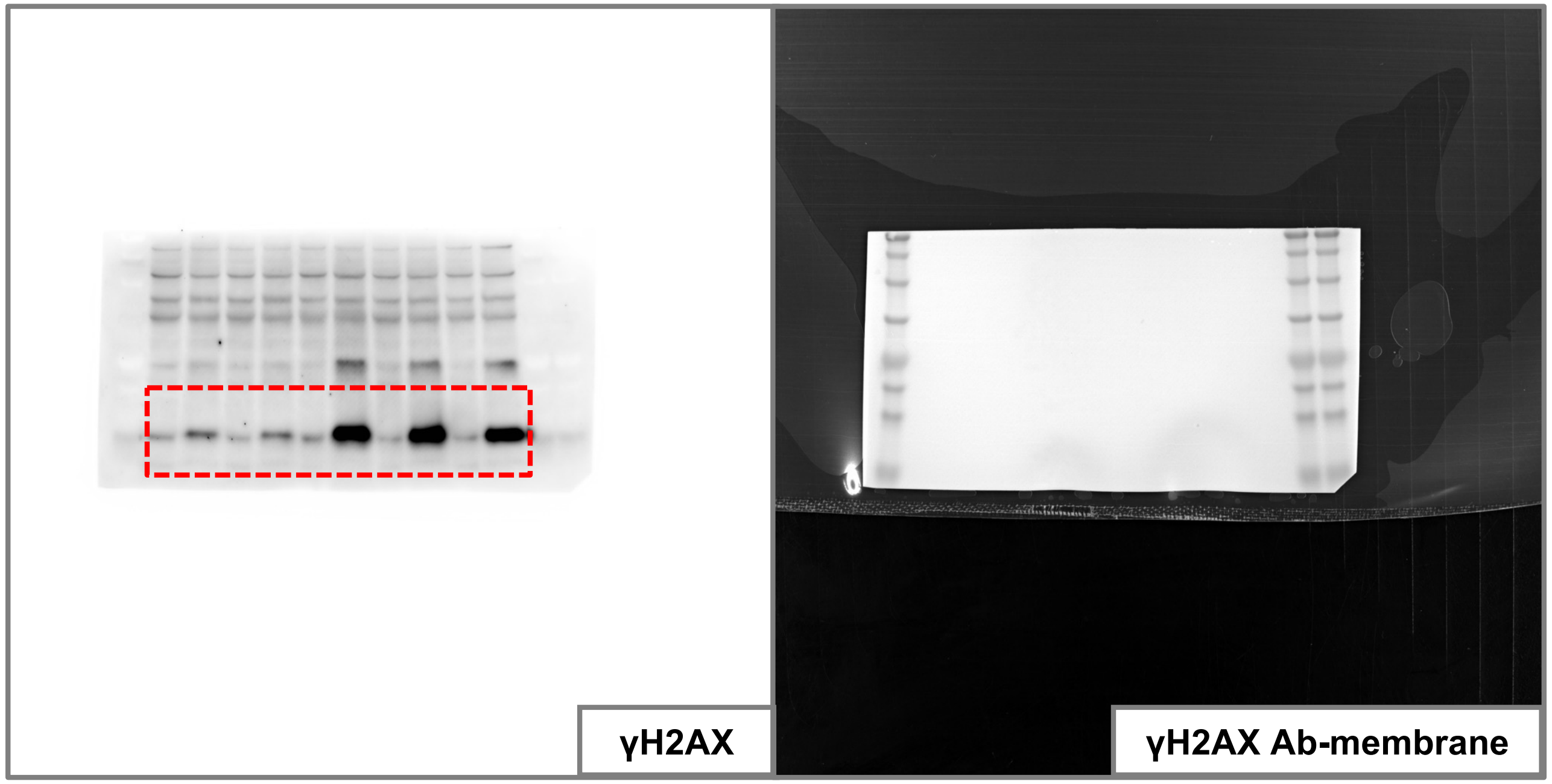

γH2AX
γH2AX Ab-membrane

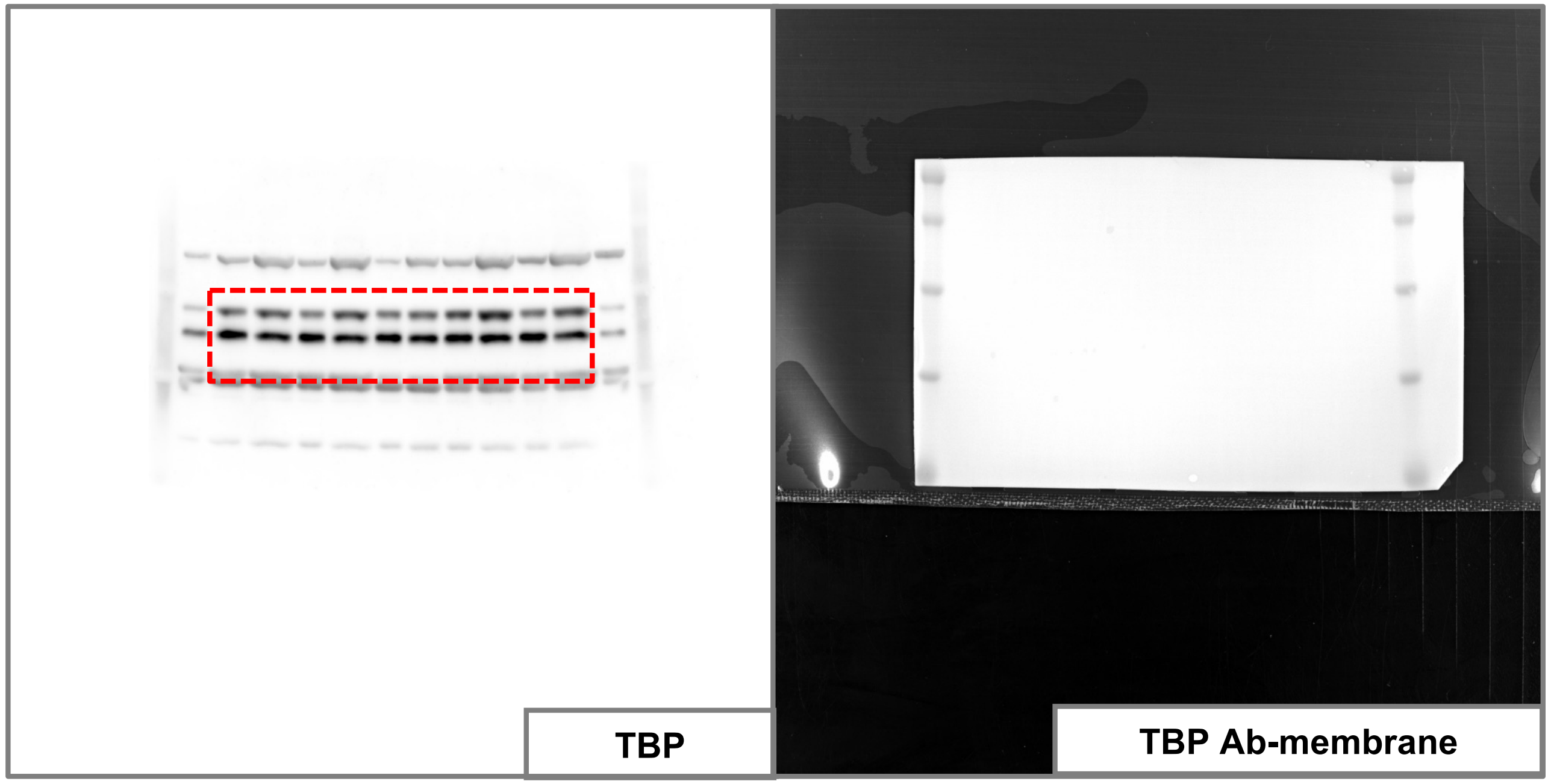

TBP
TBP Ab-membrane

Fig.S7

B

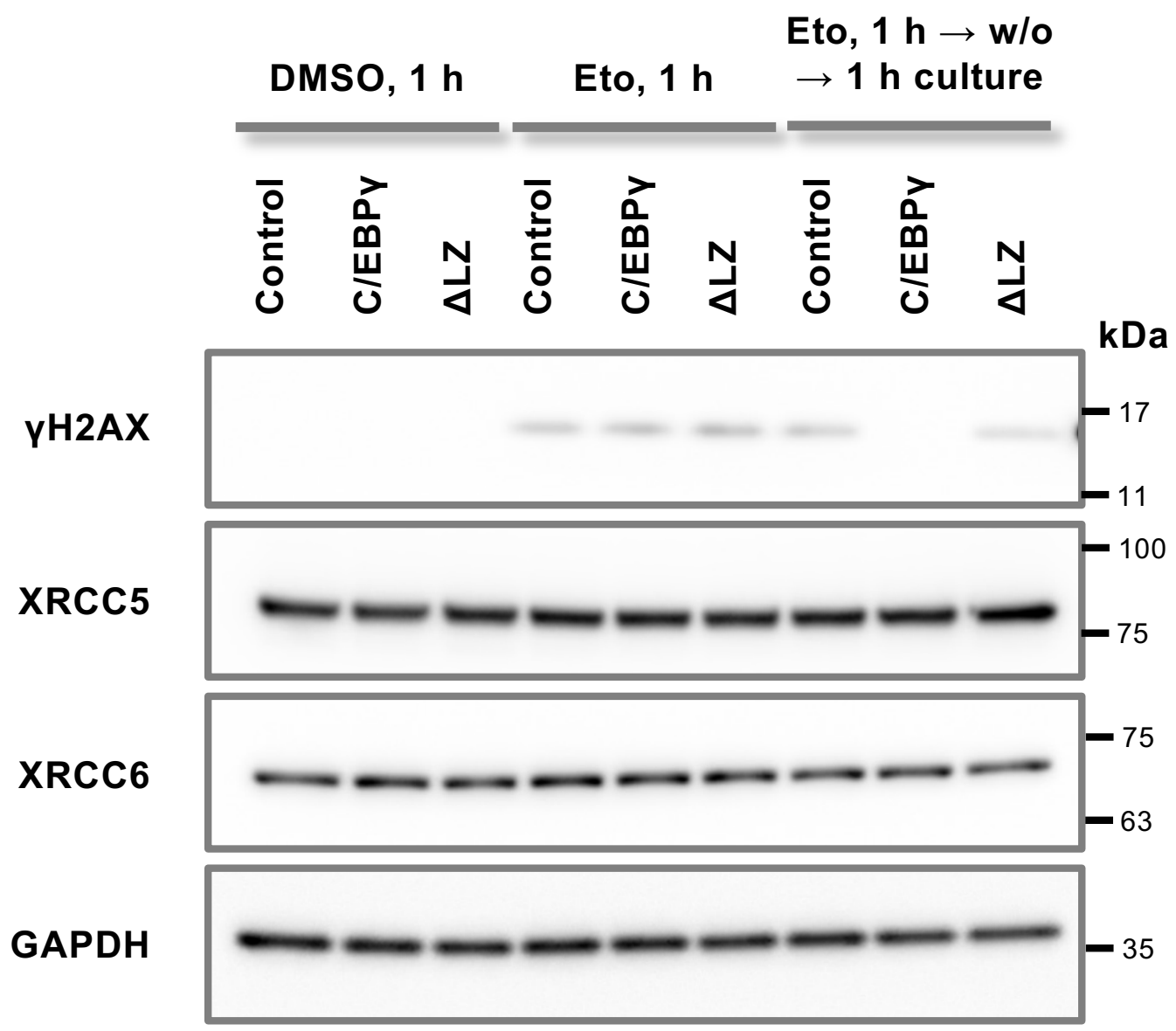
DMSO, 1 h
Eto, 1 h
Eto, 1 h → w/o → 1 h culture
Control
C/EBPγ
ΔLZ
Control
C/EBPγ
ΔLZ
Control
C/EBPγ
ΔLZ
kDa
γH2AX
17
11
XRCC5
100
75
XRCC6
75
63
GAPDH
35

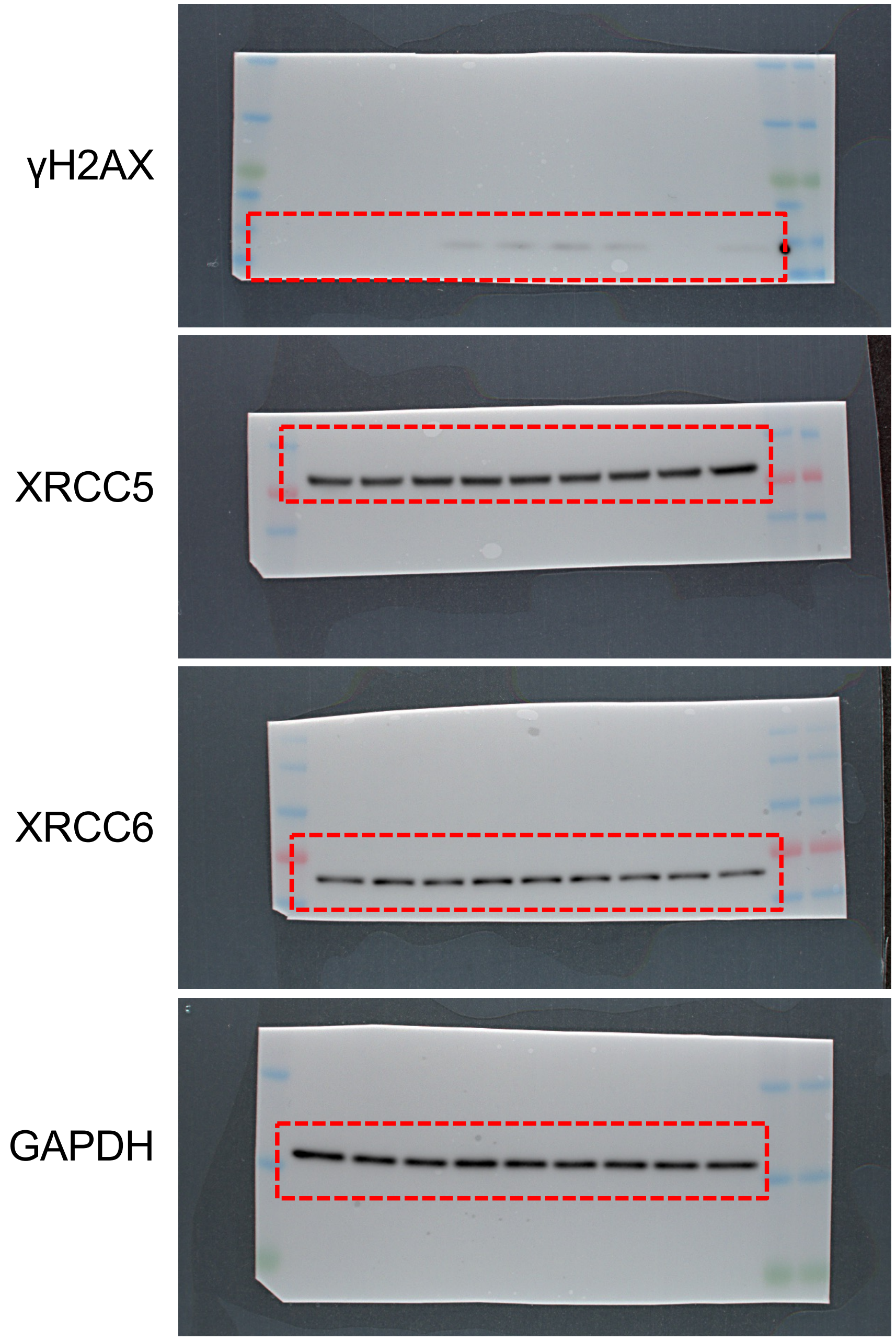
γH2AX
XRCC5
XRCC6
GAPDH

Fig.S8
