# Supplementary material for: C/EBPγ induces epithelial-mesenchymal transition and facilitates DNA double-strand break repair in lung adenocarcinoma cells
Source: Cell Death Discov. 2026 Jun 2;12:324. doi: 10.1038/s41420-026-03181-0 (PMC13443842; doi:10.1038/s41420-026-03181-0)
Supplement: Supplementary file 6 [file 41420_2026_3181_MOESM5_ESM.docx]

**Supplementary Table S4**

The sequences of oligonucleotides for shRNA-expression, cDNA cloning, quantitative RT-PCR, C/EBP-luc reporter and NHEJ assay used in this study

| **Name** | **Primer sequence (5’ to 3’)** |
| --- | --- |
| *C/EBPγ shRNA1* | F: ccgggcaacgccgagagaggaacaactcgagttgttcctctctcggcgttgcttttt  R: aattaaaaagcaacgccgagagaggaacaactcgagttgttcctctctcggcgttgc |
| *C/EBPγ shRNA2* | F: ccggttagccttgtaattcgaatatctcgagatattcgaattacaaggctaatttttg  R: aattcaaaaattagccttgtaattcgaatatctcgagatattcgaattacaaggctaa |
| *C/EBPβ shRNA1* | F: ccggcatcgacttcagcccgtacctctcgagaggtacgggctgaagtcgatgtttttg  R: aattcaaaaacatcgacttcagcccgtacctctcgagaggtacgggctgaagtcgatg |
| *C/EBPβ shRNA2* | F: ccggcgacttcctctccgacctcttctcgagaagaggtcggagaggaagtcgtttttg  R: aattcaaaaacgacttcctctccgacctcttctcgagaagaggtcggagaggaagtcg |
| *C/EBPβ shRNA3* | F: ccggcccgtggtgttatttaaagaactcgagttctttaaataacaccacgggttttt  R: aattaaaaacccgtggtgttatttaaagaactcgagttctttaaataacaccacggg |
| *XRCC5 shRNA* | F: ccggcgtgggctttaccatgagtaactcgagttactcatggtaaagcccacgtttttg  R: aattcaaaaacgtgggctttaccatgagtaactcgagttactcatggtaaagcccacg |
| *SKIL_cDNA* | F: aaagtttgagagtaagttacgataggc  R: accttgtcagtaatacacagatgaatc |
| *ETS2_cDNA* | F: gccaccgtcccgaccaag  R: tcccactcagtccacgagcc |
| *ZNF710_cDNA* | F: tcacgggatggagggcttc  R: tctccatttggtctaaaggtccc |
| *ELK3_cDNA* | F: acgcctgtcagcatggaaa  R: ttgtttcatcagttaatgagtcctta |
| *SNAI1_cDNA* | F: tggttcttctgcgctactgct  R: tatggagaggaagagggagcct |
| *VGLL3_cDNA* | F: ctggagcagcgacagaggc  R: tgcctttcgtgtcctattgctg |
| *C/EBPγ_cDNA* | F: tttcgtaaccgtcgctcctc  R: gaaaatagcctccaatgggtctt |
| *C/EBPγΔDBD_cDNA* | F: gcacaagacacactgcag  R: ttgccgatactcgtcact |
| *C/EBPγΔLZ_cDNA* | F: aaagatttgtttcttgag  R: ctgattgactctctgcag |
| *C/EBPβ_cDNA* | F: ggatccaccatggaagtggccaactt  R: aagtgccccagtgccaaag |
| *XRCC5_cDNA* | F: atggtgcggtcggggaat  R: ctatatcatgtccaataaatcgtccac |
| *XRCC6_cDNA* | F: atgtcagggtgggagtcatattac  R: tcagtcctggaagtgcttggt |
| *GAPDH_QPCR* | F: cgagatccctccaaaatcaa  R: gtcttctgggtggcagtgat |
| *DDX5_QPCR* | F: tatggttggagtggcacaga  R: ccagcaccaaacaaataggc |
| *CDH1/E-cad_QPCR* | F: tgcccagaaaatgaaaaagg  R: gtgtatgtggcaatgcgttc |
| *CDH2/N-cad _QPCR* | F: acagtggccacctacaaagg  R: ccgagatggggttgataatg |
| *VIM/Vimentin_QPCR* | F: tacaggaagctgctggaagg  R: accagagggagtgaatccag |
| *SNAI2_QPCR* | F: tcggacccacacattaccttg  R: aaaaggcttctcccccgtgt |
| *ZEB2_QPCR* | F: caagaggcgcaaacaagc  R: ggttggcaataccgtcatcc |
| *C/EBPγ_QPCR* | F: tggtggaagattgctgacc  R: aatggctttgctgagttgct |
| *C/EBPβ_QPCR* | F: acgacttcctctccgacctc  R: caggctcacgtagccgtact |
| *3×CEBP-luc* | F: ctgcagattgcgcaatctgcatgcagattgcgcaatctgcatgcagattgcgcaatctgcaa  R: gatcttgcagattgcgcaatctgcatgcagattgcgcaatctgcatgcagattgcgcaatctgcaggtac |
| *Joined-DNA_NHEJ* | F: cgtacgtctccggattcgaa  R: gtgatgcggcactcgatctt |
| *GAPDH-RT_NHEJ* | F: atgctgagtgtacaagcgttttct  R: cactatgccaccccaggaat |

F: Forward, R: Reverse
